# Synthesis of fused tricyclic amines unsubstituted at the ring-junction positions by a cascade condensation, cyclization, cycloaddition then decarbonylation strategy

**DOI:** 10.3762/bjoc.8.11

**Published:** 2012-01-18

**Authors:** Iain Coldham, Adam J M Burrell, Hélène D S Guerrand, Luke Watson, Nathaniel G Martin, Niall Oram

**Affiliations:** 1 Department of Chemistry, University of Sheffield, Brook Hill, Sheffield S3 7HF, United Kingdom; 2 AstraZeneca, Mereside, Alderley Park, Macclesfield, Cheshire SK10 4TG, United Kingdom; 3 Eli Lilly and Company, Erl Wood Manor, Windlesham, Surrey GU20 6PH, United Kingdom

**Keywords:** alkaloid, azomethine ylide, dipolar cycloaddition, heterocycle, tricyclic

## Abstract

Heating aldehydes that contain a protected hydroxymethyl group, a tethered alkyl chloride and a tethered alkenyl group at the α-position of the aldehyde with an amine sets up a cascade (tandem) reaction sequence involving condensation to an intermediate imine, then cyclization and formation of an intermediate azomethine ylide and then intramolecular dipolar cycloaddition. The fused tricyclic products are formed with complete or very high stereochemical control. The hydroxymethyl group was converted into an aldehyde – which could be removed to give the tricyclic amine products that are unsubstituted at the ring junction positions – or was converted into an alkene, which allowed the formation of the core ring system of the alkaloids scandine and meloscine.

## Introduction

**Scheme 1:**
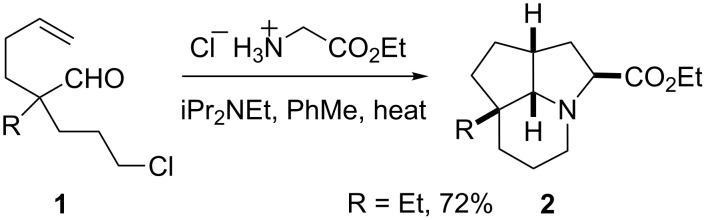
Cascade chemistry for 2-ethyl-aldehydes.

Cascade reaction sequences [1] provide a rapid and efficient means to build complexity in organic chemistry. One such sequence involves a cyclization followed by in situ intramolecular cycloaddition to give three new rings in a single transformation [2–8]. We have been studying the intramolecular dipolar cycloaddition of azomethine ylides in synthesis [9–16] and were able to show that the azomethine ylide could be prepared in situ by a cyclization step [17–18]; for example, by heating the aldehyde **1** (R = Et) with glycine ethyl ester to give the tricyclic product **2** [19–20] (Scheme 1). In this chemistry, the intermediate imine undergoes cyclization onto the tethered alkyl chloride to give an iminium ion which deprotonates to give an azomethine ylide that undergoes dipolar cycloaddition with the alkene. A single stereoisomer of product **2** was formed. Alternatively, glycine could be used and led to a tricyclic product via an intermediate azomethine ylide formed by decarboxylation. Unfortunately, when the substrate **1** (R = H) was used, heating with glycine or glycine ethyl ester did not yield the desired tricyclic products due to competing enamine formation [21].

For this cascade cyclization–cycloaddition chemistry to be widely applicable (for example for the preparation of alkaloids), we need to be able to access tricyclic products with no substituents (only hydrogen atoms) at the ring junction positions (such as compound **2**, R = H, or other ring sizes) [22]. This paper outlines an approach to such targets using a substituent (to block side reactions) which can then be removed by decarbonylation [23].

## Results and Discussion

### Preparation of the aldehyde substrates

**Scheme 2:**
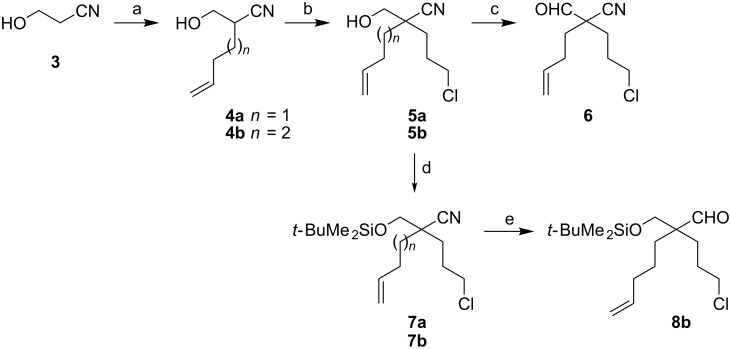
Preparation of aldehydes **6** and **8b**. Step a: LDA, THF, 0 °C, CH_2_=CHCH_2_(CH_2_)*_n_*Br (*n* = 1, 56%; *n* = 2, 76%). Step b: LDA, THF, −78 °C, ClCH_2_CH_2_CH_2_Br (*n* = 1, 80%; *n* = 2, 89%). Step c: Using **5a**, (COCl)_2_, DMSO, Et_3_N, CH_2_Cl_2_, −78 °C to rt, 92%. Step d: TBSCl, imidazole, CH_2_Cl_2_, rt, 16 h (*n* = 1, 92%; *n* = 2, 94%). Step e: Using **7b**, DIBAL-H, CH_2_Cl_2_, −78 °C, 2 h then HCl_(aq)_ (2 M), 62%.

**Scheme 3:**
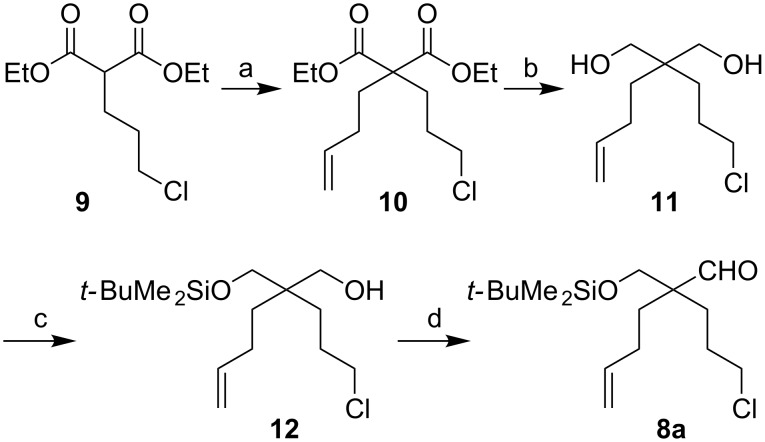
Preparation of aldehyde **8a**. Step a: NaH, THF, 0 to 70 °C, CH_2_=CHCH_2_CH_2_Br. Step b: DIBAL-H, CH_2_Cl_2_, 0 °C, 2 h then HCl_(aq)_ (2 M), 24% over two steps. Step c: TBSCl, imidazole, CH_2_Cl_2_, rt, 16 h, 86%. Step d: (COCl)_2_, DMSO, Et_3_N, CH_2_Cl_2_, −78 °C to rt, 96%.

We decided to incorporate a one-carbon substituent, alpha to the aldehyde group, that could potentially be removed after the cascade cyclization–cycloaddition chemistry. Starting from nitrile **3**, we prepared three different aldehydes, as shown in Scheme 2 and Scheme 3. Double deprotonation (of the alcohol and alpha to the nitrile) of substrate **3** and C-alkylation occurred smoothly to give **4a** or **4b** (*n* = 1 or 2, respectively). A second double deprotonation and then an alkylation with bromochloropropane gave **5a** and **5b**. Oxidation of the alcohol **5a** (*n* = 1) gave the aldehyde **6**. Alternatively, protection of **5a** and **5b** as their *tert*-butyldimethylsilyl ethers gave **7a** and **7b**. DIBAL-H reduction of nitrile **7b** (*n* = 2) gave aldehyde **8b**. The same reduction of nitrile **7a** gave a low yield of the desired aldehyde **8a**; so, an alternative strategy to prepare the aldehyde with one less methylene group in the side-chain was studied (Scheme 3). Alkylation of the commercially available substituted malonate **9** gave product **10** together with diethyl cyclobutanedicarboxylate (formed by cyclization of the malonate anion onto the internal alkyl chloride). This by-product was removed by column chromatography after reduction of both esters to give diol **11**. Mono-protection gave the alcohol **12** which was oxidized to aldehyde **8a**.

### Cascade condensation, cyclization, cycloaddition

**Scheme 4:**
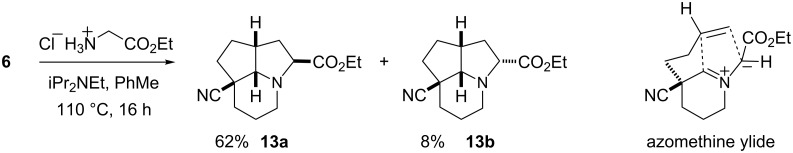
Cascade chemistry with aldehyde **6** and preferred conformation of intermediate azomethine ylide during cycloaddition.

The cascade tricyclic ring formation was then investigated with the aldehydes **6**, **8a** and **8b**. Heating aldehyde **6** with glycine gave a mixture of unidentifiable products. However, heating with glycine ethyl ester in toluene gave the desired tricyclic product **13a** as the major stereoisomer, together with a small amount of the separable stereosiomer **13b** (Scheme 4). The major isomer **13a** was assumed to have the all-*cis* configuration based on related chemistry (compare with product **2** [19–20], Scheme 1). The minor isomer was assigned by NOESY studies, in which there was a strong enhancement between the ring junction protons and a strong enhancement between the proton alpha to the ester and the ring junction proton alpha to the nitrogen. The reaction is supposed to proceed by initial imine formation, then cyclization with displacement of chloride to give an iminium ion. Deprotonation of this iminium ion would give the azomethine ylide that undergoes intramolecular cycloaddition. The preference for the all-*cis* isomer **13a** arises from a preference for cycloaddition from the conformation shown in Scheme 4. Unfortunately, we were not able to remove the nitrile substituent from the product **13a** using lithium in ammonia [24], which instead converted the ethyl ester to its primary carboxylic amide, or using DIBAL-H, which gave a complex mixture of products.

**Scheme 5:**
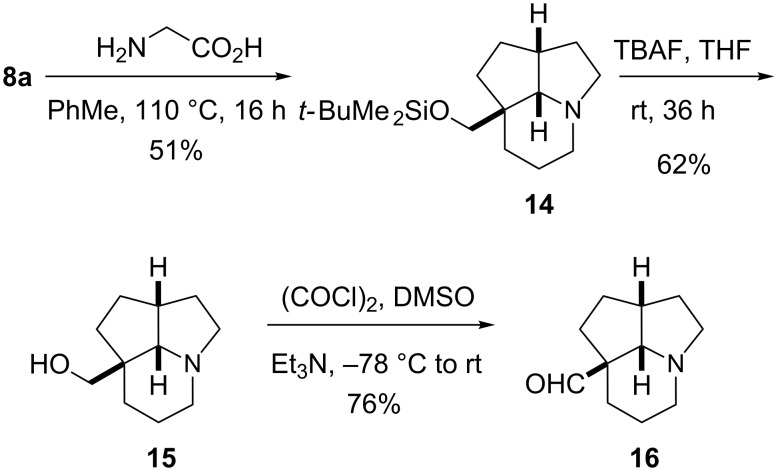
Cascade chemistry with aldehyde **8a** and glycine.

Heating aldehyde **8a** with glycine gave cycloadduct **14** as a single diastereoisomer (Scheme 5). Deprotection of the silyl ether with tetra-*n*-butylammonium fluoride (TBAF) gave the alcohol **15**, then Swern oxidation yielded aldehyde **16**. The stereochemistry of alcohol **15** was confirmed by single crystal X-ray analysis. This stereochemistry was expected for these cascade reactions, in which the substituent alpha to the aldehyde (in this case the TBSOCH_2_ group) is located in the transition state *cis* to the developing ring junction protons. The next step, to remove the one-carbon unit to provide the desired tricyclic product with all ring-junction protons, was a decarbonylation and this type of reaction is known using Wilkinson’s catalyst [25]. We were disappointed to find that attempts to conduct this decarbonylation using aldehyde **16** and [Rh(PPh_3_)_3_Cl] resulted only in decomposition.

**Scheme 6:**
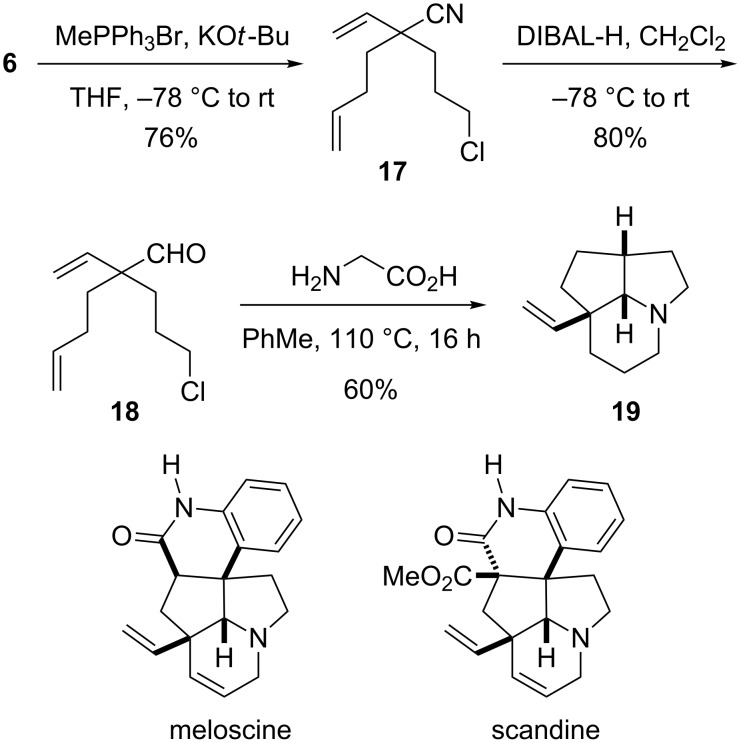
Synthesis of the core tricyclic ring system of meloscine and scandine.

As an aside, we were interested in using the products of this chemistry for the construction of the core ring system found in alkaloid natural products. Wittig reaction with aldehyde **16** should be possible to provide the core ring system found in the alkaloids meloscine and scandine [26–30]. However, we chose to investigate a shorter reaction sequence without the need for protecting groups and to carry out the Wittig reaction at an earlier stage, as shown in Scheme 6. Olefination of aldehyde **6** with the anion formed from methyltriphenylphosphonium bromide gave alkene **17**, which was reduced with DIBAL-H to give aldehyde **18**. Heating this aldehyde with glycine in toluene gave the desired tricyclic product **19** as a single stereoisomer. NOESY studies verified the stereochemistry as shown. Only one of the two alkene groups acts as the dienophile, as expected for conformational reasons. This synthesis (six steps from nitrile **3**) represents an efficient entry to the core of the alkaloids meloscine and scandine, which contains the same fused tricyclic ring system.

The use of glycine to form a ‘non-stabilized’ ylide had led to the desired cycloadduct **14**, although subsequent decarbonylation had not been successful using aldehyde **16** (Scheme 5). Despite this, we chose to investigate the cascade chemistry using glycine ethyl ester (to form a ‘stabilized’ ylide), followed by possible later decarbonylation.

**Scheme 7:**
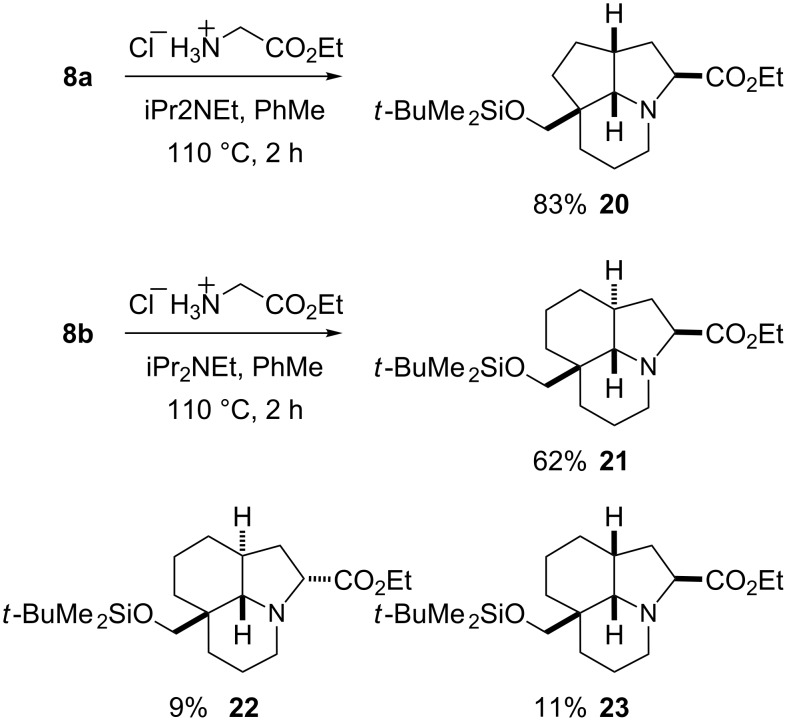
Cascade chemistry with aldehydes **8a** and **8b** and glycine ethyl ester.

Heating aldehydes **8a** or **8b** with glycine ethyl ester in toluene gave the expected tricyclic products (Scheme 7). Using aldehyde **8a**, a single stereoisomer was formed (assumed to have the all-*cis* configuration shown and confirmed by NOESY studies). Using aldehyde **8b**, the major product was the stereoisomer **21**, which was expected based on related chemistry [19] and verified by the large coupling constant in the ^1^H NMR spectrum for the ring junction proton alpha to the nitrogen atom (2.20 ppm, doublet, *J* = 11.5 Hz). However, this transformation was not entirely stereoselective and a (separable) mixture of two other stereoisomers **22** and **23** was also formed (NOESY studies were used to ascertain the relative stereochemistry).

**Scheme 8:**
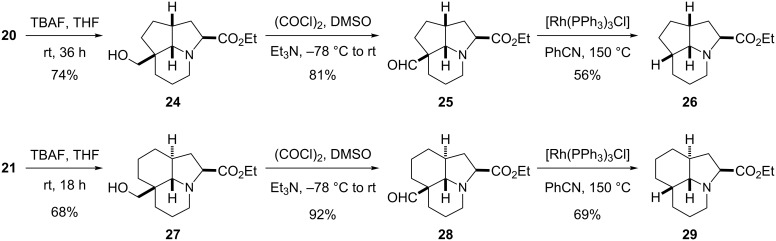
Decarbonylation reactions to give the products **26** and **29**.

To complete the desired aim to provide a method to prepare fused tricyclic products with all ring-junction protons, we carried out the transformations shown in Scheme 8. Desilylation of **20** and **21** gave the alcohols **24** and **27**, respectively. Swern oxidation then gave the aldehydes **25** and **28**. Finally, using Wilkinson’s catalyst, we were pleased to find that both these aldehyde substrates were amenable to decarbonylation. The optimum conditions were found to be a temperature of about 150 °C for about 2 h. The decarbonylation occurred with overall retention of stereochemistry, as verified by NOESY studies. The presence of the ethyl ester substituent must be sufficient to allow a successful decarbonylation (in comparison with substrate **16**), possibly due to the reduced ability of the amine nitrogen atom to coordinate to the rhodium complex.

## Conclusion

The chemistry described here provides a method to prepare fused tricyclic products in which the ring junction positions are unsubstituted. Problems with conducting the cascade condensation, cyclisation, cycloaddition chemistry using azomethine ylides derived from enolisable aldehyde substrates [21] has been circumvented by incorporating a one-carbon unit at the alpha-position of the aldehyde. This one-carbon unit can be removed in certain cases using a decarbonylation reaction.

## Supporting Information

File 1 Experimental procedures and spectroscopic data.
