# Supplementary material for: Synthesis of fused tricyclic amines unsubstituted at the ring-junction positions by a cascade condensation, cyclization, cycloaddition then decarbonylation strategy
Source: Beilstein J Org Chem. 2012 Jan 18;8:107–11. doi: 10.3762/bjoc.8.11 (PMC3302069; doi:10.3762/bjoc.8.11)
Supplement: File 1 — Experimental procedures and spectroscopic data. [file Beilstein_J_Org_Chem-08-107-s001.pdf]

Supporting Information

for

Synthesis of fused tricyclic amines unsubstituted at the ring-junction positions by a cascade condensation, cyclization, cycloaddition then decarbonylation strategy

Iain Coldham*[1], Adam J. M. Burrell[1], Hélène D. S. Guerrand[1], Luke Watson[1], Nathaniel G. Martin[2] and Niall Oram[3]

Address: [1]Department of Chemistry, University of Sheffield, Brook Hill, Sheffield S3 7HF, United Kingdom; [2]AstraZeneca, Mereside, Alderley Park, Macclesfield, Cheshire SK10 4TG, United Kingdom and [3]Eli Lilly and Company, Erl Wood Manor, Windlesham, Surrey GU20 6PH, United Kingdom

Email: Iain Coldham - i.coldham@sheffield.ac.uk

* Corresponding author

Experimental procedures and spectroscopic data

Table of contents

General experimental details S-2

Experimental procedures and characterization data S-2

NMR spectra S-17

General experimental details

All reagents were obtained from commercial suppliers and were used without further purification unless otherwise specified. Solvents were purified using a Grubbs dry solvent system (model SPS-200-6). Petrol refers to petroleum ether (bp 40–60 °C). Thin layer chromatography was performed on silica plates and visualized by UV irradiation at 254 nm or by staining with an alkaline $KMnO_4$ dip. Column chromatography was performed using silica gel (40–63 micron mesh). Infrared spectra were recorded on Perkin Elmer Spectrum RX Fourier Transform IR System. ^{1}H NMR spectra were recorded on a Bruker AC400 (400 MHz) instrument. Chemical shifts are reported in ppm with respect to the residual solvent peaks, with multiplicities given as s = singlet, d = doublet, t = triplet, q = quartet, m = multiplet. Coupling constants, *J*, are quoted to the nearest 0.5 Hz. ^{13}C NMR were recorded on the above mentioned instrument at 100 MHz. Low and high resolution mass spectra (accurate mass) were recorded on a Walters LCT instrument for Electro–Spray (ES).

Experimental procedures and characterization data

2-(Hydroxymethyl)hex-5-enenitrile (4a): *n*-Butyllithium (5.41 mL, 13.5 mmol, 2.5 M in hexanes) was added to diisopropylamine (2.02 mL, 14.2 mmol) in THF (30 mL) at 0 °C. After 10 min, 3-hydroxypropionitrile (0.45 mL, 6.64 mmol) was added and the mixture was allowed to warm to room temperature. After 10 min, 4-bromo-1-butene (1.01 mL, 9.9 mmol) was added quickly. After 2 h, saturated aqueous ammonium chloride solution (20 mL) was added and the mixture was extracted with EtOAc (3 × 60 mL). The organic layers were combined, dried ($MgSO_4$) and evaporated. Purification by column chromatography, eluting with petrol–EtOAc (8:2), gave nitrile **4a** (0.46 g, 56%) as an oil; R_f 0.20 [petrol–EtOAc (8:2)]; ν_{max}/cm^{-1} 3340, 2920, 2880,

2360, 1455; ^{1}H NMR (400 MHz, $CDCl_3$) δ = 5.79 (1H, ddt, *J* = 17, 10, 6.5 Hz), 5.18–5.06 (2H, m), 3.81 (2H, t, *J* = 5.5 Hz), 2.81 (1H, dq, *J* = 9.5, 5.5 Hz), 2.45–2.19 (2H, m), 1.90–1.64 (2H, m); ^{13}C NMR (100 MHz, $CDCl_3$) δ = 136.9, 120.6, 116.6, 62.6, 34.3, 30.9, 27.6; HRMS (EI) Found: M^+, 125.0841. $C_7H_{11}NO$ requires M^+, 125.0843.

2-(Hydroxymethyl)hept-6-enenitrile (4b): In the same way as nitrile **4a**, *n*-butyllithium (18.8 mL, 47 mmol), diisopropylamine (6.96 mL, 49.3 mmol), 3-hydroxypropionitrile (1.58 mL, 22.9 mmol) and 5-bromo-1-pentene (3.0 mL, 24 mmol) gave, after purification by column chromatography, eluting with petrol–EtOAc (4:1), nitrile **4b** (2.33 g, 76%) as an oil; R_f 0.26 [petrol–EtOAc (3:1)]; $ν_{max}/cm^{-1}$ 3430, 3075, 2930, 2860, 2245, 1640, 1460; ^{1}H NMR (400 MHz, $CDCl_3$) δ = 5.76 (1H, ddt, *J* = 17, 10.5, 6.5 Hz), 5.05–4.95 (2H, m), 3.74 (2H, t, *J* = 6 Hz), 2.77–2.69 (1H, m), 2.48 (1H, t, *J* = 6 Hz), 2.15–2.04 (2H, m), 1.70–1.49 (4H, m); ^{13}C NMR (100 MHz, $CDCl_3$) δ = 137.5, 120.9, 115.5, 62.6, 35.0, 33.1, 27.8, 26.2; HRMS (EI) Found: M^+, 139.0997. $C_8H_{13}NO$ requires M^+, 139.0997.

2-(3-Chloropropyl)-2-(hydroxymethyl)hex-5-enenitrile (5a): *n*-Butyllithium (14.88 mL, 37.2 mmol, 2.5 M in hexanes) was added to diisopropylamine (5.61 mL, 39.7 mmol) in THF (40 mL) at –78 °C. After 10 min, nitrile **4a** (1.55 g, 12.4 mmol) was added. After 10 min, 1-bromo-3-chloropropane (3.10 mL, 31.0 mmol) was added and the mixture was allowed to warm to 0 °C. After 1 h, saturated aqueous ammonium chloride solution (20 mL) was added and the mixture was extracted with EtOAc (3 × 60 mL). The organic layers were combined, dried ($MgSO_4$) and evaporated. Purification by column chromatography, eluting with petrol–EtOAc (8:2), gave nitrile **5a** (1.99 g, 80%) as an oil; R_f 0.30 [petrol–EtOAc (8:2)]; $ν_{max}/cm^{-1}$ 3410, 2950, 2236, 1450; ^{1}H NMR (400 MHz, $CDCl_3$) δ = 5.84 (1H, ddt, *J* = 17, 10, 6.5 Hz), 5.16–5.03

(2H, m), 3.79–3.69 (2H, m), 3.60 (2H, t, *J* = 6 Hz), 2.30–2.22 (2H, m), 2.04–1.68 (6H, m); ^{13}C NMR (100 MHz, $CDCl_3$) δ = 136.7, 121.9, 115.9, 65.3, 44.5, 43.0, 32.8, 31.1, 28.7, 27.7; HRMS (ES) Found: MH^+, 202.0999. $C_{10}H_{17}NO^{35}Cl$ requires MH^+, 202.0992.

2-(3-Chloropropyl)-2-(hydroxymethyl)hept-6-enenitrile (5b): In the same way as nitrile **5a**, *n*-butyllithium (5.81 mL, 14.2 mmol), diisopropylamine (2.05 mL, 14.5 mmol), nitrile **4b** (660 mg, 4.74 mmol) and 1-bromo-3-chloropropane (0.94 mL, 9.49 mmol) gave, after purification by column chromatography, eluting with petrol–EtOAc (5:1), nitrile **5b** (906 mg, 89%) as an oil; R_f 0.55 [petrol–EtOAc (7:3)]; ν_{max}/cm^{-1} 3450, 3075, 2940, 2860, 2235, 1640, 1460; ^{1}H NMR (250 MHz, $CDCl_3$) δ = 5.77 (1H, ddt, *J* = 17, 10, 6.5 Hz), 5.08–4.94 (2H, m), 3.67 (1H, d, *J* = 4.5 Hz), 3.65 (1H, d, *J* = 4.5 Hz), 3.57 (2H, t, *J* = 6 Hz), 2.44–1.45 (10H, m); ^{13}C NMR (63 MHz, $CDCl_3$) δ = 137.5, 122.2, 115.5, 65.4, 44.5, 43.2, 33.5, 33.0, 31.1, 27.7, 23.6; HRMS (EI) Found: MH^+, 216.1154. $C_{11}H_{19}NO^{35}Cl$ requires MH^+, 216.1155.

2-(3-Chloropropyl)-2-formylhex-5-enenitrile (6): Oxalyl chloride (0.53 mL, 5.28 mmol) in CH_2Cl_2 (55 mL) was cooled to −78 °C. DMSO (0.93 mL, 13.1 mmol) in CH_2Cl_2 (6 mL) was added dropwise and after 5 min, alcohol **5a** (1.10 g, 5.46 mmol) in CH_2Cl_2 (6 mL) was added dropwise. After 5 min, Et_3N (3.91 mL, 28.2 mmol) was added dropwise. After 15 min, the mixture was allowed to warm to room temperature. After 30 min, the mixture was diluted with CH_2Cl_2 and washed with water (50 mL) and brine (50 mL). The organic layer was dried ($MgSO_4$) and evaporated. Purification by column chromatography, eluting with petrol–EtOAc (17:3), gave aldehyde **6** (0.99 g, 92%) as an oil; R_f 0.20 [petrol–EtOAc (17:3)]; ν_{max}/cm^{-1} 2960, 2360, 1735, 1450; ^{1}H NMR (400 MHz, $CDCl_3$) δ = 9.44 (1H, s), 5.76 (1H, ddt, *J* = 17, 10, 6.5 Hz), 5.19–

5.03 (2H, m), 3.60 (2H, t, *J* = 6 Hz), 2.41–2.28 (1H, m), 2.25–2.11 (1H, m), 2.11–1.79 (6H, m); ^{13}C NMR (100 MHz, $CDCl_3$) δ = 193.8, 135.6, 117.8, 117.1, 53.3, 43.8, 33.3, 31.0, 29.1, 27.9; HRMS (ES) Found: M^+, 199.0764. $C_{10}H_{14}NO^{35}Cl$ requires M^+, 199.0768.

2-(*tert*-Butyldimethylsilyloxymethyl)-2-(3-chloropropyl)hex-5-enenitrile (7a): Nitrile **5a** (1.99 g, 9.9 mmol) and imidazole (1.00 g, 14.0 mmol) were dissolved in CH_2Cl_2 (20 mL) and cooled to 0 °C. After 10 min, TBSCl (1.94 g, 12.9 mmol) was added and the mixture was allowed to warm to room temperature over 16 h. The mixture was diluted with CH_2Cl_2 and washed with water (2 × 30 mL) and brine (30 mL). The organic layer was dried ($MgSO_4$) and evaporated. Purification by column chromatography, eluting with petrol–EtOAc (49:1), gave nitrile **7a** (2.87 g, 92%) as an oil; R_f 0.30 [petrol–EtOAc (49:1)]; ν_{max}/cm^{-1} 2930, 2860, 2365, 1470, 1265, 1100; ^{1}H NMR (400 MHz, $CDCl_3$) δ = 5.84 (1H, ddt, *J* = 17, 10, 6.5 Hz), 5.14–5.01 (2H, m), 3.69–3.67 (2H, m), 3.60 (2H, t, *J* = 6.5 Hz), 2.28–2.19 (2H, m), 2.03–1.64 (6H, m), 0.93 (9H, s), 0.11 (6H, s); ^{13}C NMR (100 MHz, $CDCl_3$) δ = 137.0, 122.0, 115.6, 65.0, 44.6, 42.7, 33.1, 31.4, 28.8, 27.7, 25.7, 18.2, −5.6; HRMS (ES) Found: MH^+, 316.1863. $C_{16}H_{31}NOSi^{35}Cl$ requires MH^+, 316.1861.

2-(*tert*-Butyldimethylsilanyloxymethyl)-2-(3-chloropropyl)hept-6-enenitrile (7b): In the same way as nitrile **7a**, nitrile **5b** (1.178 g, 5.46 mmol), imidazole (563 mg, 8.19 mmol) and TBSCl (1.10 g, 7.10 mmol) gave, after purification by column chromatography, eluting with petrol–EtOAc (97:3), nitrile **7b** (1.69 g, 94%) as an oil; R_f 0.48 [petrol–EtOAc (19:1)]; ν_{max}/cm^{-1} 2955, 2930, 2860, 2235, 1640, 1115, 775; ^{1}H NMR (400 MHz, $CDCl_3$) δ = 5.77 (1H, ddt, *J* = 17, 10, 6.5 Hz), 5.06–4.95 (2H, m), 3.62 (1H, d, *J* = 10 Hz), 3.60 (1H, d, *J* = 10 Hz), 3.56 (2H, t, *J* = 6 Hz), 2.08 (2H, q, *J*

= 6.5 Hz), 1.96–1.87 (2H, m), 1.83–1.47 (6H, m), 0.89 (9H, s), 0.07 (6H, s); ^{13}C NMR (100 MHz, $CDCl_3$) δ = 137.7, 122.3, 115.4, 65.0, 44.7, 42.9, 33.6, 33.2, 31.4, 27.6, 25.7, 23.7, 18.2, −5.58; HRMS (ES) Found: MH^+, 330.2007. $C_{17}H_{33}NOSi^{35}Cl$ requires MH^+, 330.2020.

2-(tert-Butyldimethylsilyloxymethyl)-2-(3-chloropropyl)hex-5-enal (8a): In the same way as aldehyde **6**, oxalyl chloride (0.48 mL, 5.66 mmol), DMSO (0.77 mL, 10.9 mmol) and alcohol **12** (1.45 g, 4.53 mmol) gave, after purification by column chromatography, eluting with petrol–EtOAc (19:1), aldehyde **8a** (1.39 g, 96%) as an oil; R_f 0.20 [petrol–EtOAc (19:1); ν_{max}/cm^{-1} 2950, 2930, 1725, 1470, 1250; ^1H NMR (400 MHz, $CDCl_3$) δ = 9.55 (1H, s), 5.79 (1H, ddt, *J* = 17, 10, 6.5 Hz), 5.08–4.96 (2H, m), 3.72 (1H, d, *J* = 10 Hz), 3.67 (1H, d, *J* = 10 Hz) 3.58–3.51 (2H, m), 2.04–1.93 (2H, m), 1.74–1.68 (4H, m), 1.67–1.61 (2H, m), 0.89 (9 H, s), 0.06 (6H, s); ^{13}C NMR (100 MHz, $CDCl_3$) δ = 205.7, 138.0, 115.0, 63.8, 53.7, 45.3, 28.9, 27.7, 26.9, 26.7, 25.7, 18.1, −5.7; HRMS (ES) Found: MH^+, 319.1852, $C_{16}H_{32}O_2Si^{35}Cl$ requires MH^+, 319.1860.

2-(*tert*-Butyldimethylsilanyloxymethyl)-2-(3-chloropropyl)hept-6-enal (8b): To the nitrile **7b** (1.59 g, 4.82 mmol) in CH_2Cl_2 (20 mL) at −78 °C was added dropwise DIBAL-H (12.5 mL, 12.5 mmol, 1.0 M in hexanes). After 1.5 h, aqueous HCl (2 M, 15 mL) was added. After 30 min, the mixture was allowed to warm to room temperature. After 30 min, the mixture was extracted with Et_2O (40 mL). The organic layer was washed with aqueous HCl (2 M, 20 mL) and the aqueous portions were then extracted with Et_2O (5 × 75 mL). The organic layers were dried ($MgSO_4$) and evaporated. Purification by column chromatography, eluting with petrol–EtOAc (97:3), gave the aldehyde **8b** (998 mg, 62%) as an oil; R_f 0.63 [petrol–EtOAc (19:1)];

ν_{max}/cm^{-1} 2930, 2855, 1725, 1640, 775; ^{1}H NMR (400 MHz, $CDCl_3$) δ = 9.50 (1H, s), 5.75 (1H, ddt, *J* = 17.0, 10.5, 6.5 Hz), 5.03–4.93 (2H, m), 3.67 (1H, d, *J* = 10.5 Hz), 3.61 (1H, d, *J* = 10.5 Hz), 3.53–3.48 (2H, m), 2.03 (2H, qt, *J* = 6.5, 1.5 Hz), 1.68–1.64 (4H, m), 1.53–1.46 (2H, m), 1.32–1.23 (2H, m), 0.85 (9H, s), 0.02 (6H, s); ^{13}C NMR (100 MHz, $CDCl_3$) δ = 205.9, 138.1, 115.1, 63.9, 53.8, 45.3, 34.2, 29.0, 26.8 (2 × CH_2), 25.7, 22.6, 18.1, −5.7; HRMS (ES) Found: MH^+, 333.2029. $C_{17}H_{34}O_2Si^{35}Cl$ requires MH^+, 333.2017.

Diethyl 2-(but-3-enyl)-2-(3-chloropropyl)propanedioate (10): To a stirred solution of diethyl 3-(3-chloropropyl)malonate (10.0 mL, 46.6 mmol) and 1-bromobutene (18.9 mL, 186 mmol) in THF (250 mL) at 0 °C was added NaH (9.0 g, 230 mmol) portion-wise over 1 h. After a further 1 h, the mixture was heated under reflux. After 16 h, saturated aqueous ammonium chloride solution (100 mL) was added carefully and the mixture was extracted with Et_2O (3 × 300 mL). The organic layers were combined, dried ($MgSO_4$) and evaporated. Purification by column chromatography, eluting with petrol–EtOAc (49:1), gave malonates **10** and diethyl cyclobutane-1,1-dicarboxylate (6.68 g) as an inseparable mixture (ratio 1:1) as an oil, which was taken on to the next stage.

2-(But-3-enyl)-2-(3-chloropropyl)propane-1,3-diol (11): DIBAL-H (62.5 mL, 62.5 mmol, 1.0 M in hexanes) was added dropwise to the mixture of malonates above (3.34 g) in CH_2Cl_2 (63 mL) at 0 °C and the mixture was allowed to warm to room temperature over 2 h. Aqueous sodium hydroxide (2 M, 50 mL) was added and the mixture was extracted with Et_2O (6 × 50 mL). The organic layers were combined, dried ($MgSO_4$), and evaporated. Purification by column chromatography, eluting with petrol–EtOAc (7:3 to 1:1), gave diol **11** (1.21 g, 24% over 2 steps) as an oil; R_f 0.30

[petrol–EtOAc (1:1)]; ν_{max}/cm^{-1} 3350, 2930, 1450; ^{1}H NMR (400 MHz, $CDCl_3$) δ = 5.83 (1H, ddt, *J* = 17, 10, 6.5 Hz), 5.10–4.96 (2H, m), 3.67–3.54 (6H, m), 2.30 (2H, bs), 2.07–1.99 (2H, m), 1.82–1.72 (2H, m), 1.52–1.46 (2H, m), 1.40–1.43 (2H, m); ^{13}C NMR (100 MHz, $CDCl_3$) δ = 138.7, 114.6, 68.8, 45.7, 41.0, 30.2, 28.0, 27.3, 26.4; HRMS (ES) Found: MH^+, 207.1143. $C_{10}H_{20}O_2{}^{35}Cl$ requires MH^+, 207.1152.

2-(*tert*-Butyldimethylsilyloxymethyl)-2-(3-chloropropyl)hex-5-en-1-ol (12): Diol **11** (1.10 g, 5.82 mmol) was added to 4-dimethylaminopyridine (0.13 g, 1.06 mmol) and imidazole (0.43 g, 6.34 mmol) in CH_2Cl_2 (60 mL) and cooled to 0 °C. After 10 min, TBSCl (0.88 g, 5.80 mmol) was added and the mixture was allowed to warm to room temperature over 16 h. The mixture was diluted with CH_2Cl_2 and washed with water (3 × 50 mL). The organic layers were combined, dried ($MgSO_4$), and evaporated. Purification by column chromatography, eluting with petrol–EtOAc (9:1), gave alcohol **12** (1.45 g, 86%) as an oil; R_f 0.30 [petrol–EtOAc (9:1)]; ν_{max}/cm^{-1} 3350, 2930, 1450; ^{1}H NMR (250 MHz, $CDCl_3$) δ = 5.82 (1H, ddt, *J* = 17, 10, 6.5 Hz), 5.10–4.91 (2H, m), 3.62–3.47 (6H, m), 2.79 (1H, t, *J* = 5.5 Hz), 2.14–1.89 (2H, m), 1.86–1.62 (2H, m), 1.50–1.13 (4H, m), 0.91 (9H, s), 0.09 (6H, s); ^{13}C NMR (63 MHz, $CDCl_3$) δ = 138.8, 114.4, 69.4, 68.6, 45.7, 41.0, 30.3, 28.3, 27.3, 26.6, 25.8, 18.1, −5.7; HRMS (ES) Found: MH^+, 321.2017. $C_{16}H_{34}O_2Si^{35}Cl$ requires MH^+, 321.2017.

6a-Cyanodecahydrocyclopenta[*hi*]indolizine-2-carboxylic acid ethyl ester (13): Aldehyde **6** (445 mg, 2.23 mmol), glycine ethyl ester hydrochloride (467 mg, 3.34 mmol) and *N,N*-diisopropylethylamine (0.75 mL, 6.70 mmol) in PhMe (23 mL) were heated under reflux. After 14 h, the solvent was evaporated. Purification by column chromatography, eluting with petrol–EtOAc (9:1), gave cycloadduct **13a** (344 mg, 62%) as an oil and cycloadduct **13b** (44 mg, 8%) as an oil; data for **13a**:

R_f 0.49 [petrol–EtOAc (8:2)]; v_{max}/cm^{-1} 2925, 2230, 1735; ^1H NMR (400 MHz, $CDCl_3$) δ = 4.24–4.13 (2H, m), 3.77 (1H, t, *J* = 7 Hz), 3.73 (1H, d, *J* = 6 Hz), 3.00–2.81 (3H, m), 2.34–2.12 (3H, m), 2.08–1.98 (1H, m), 1.93–1.81 (2H, m), 1.70–1.58 (3H, m), 1.48–1.43 (1H, m), 1.29 (3H, t, *J* = 7 Hz); ^{13}C NMR (100 MHz, $CDCl_3$) δ = 171.5, 124.6, 69.1, 64.0, 60.6, 45.0, 42.8, 40.5, 37.8, 32.8, 32.7, 29.3, 17.7, 14.4; HRMS (ES) Found: 249.1594. $C_{14}H_{21}N_2O_2$ requires MH^+, 249.1603; data for **13b**: R_f 0.67 [petrol–EtOAc (8:2)]; v_{max}/cm^{-1} 2925, 2230, 1735; ^1H NMR (500 MHz, $CDCl_3$) δ = 4.19–4.09 (2H, m), 3.15 (1H, t, *J* = 8 Hz), 3.04–3.01 (1H, m), 2.76 (1H, d, *J* = 5.5 Hz), 2.68–2.62 (1H, m), 2.46–2.34 (2H, m), 2.24–2.18 (2H, m), 2.05 (1H, dt, *J* = 13.5, 4 Hz), 1.88–1.72 (3H, m), 1.61–1.54 (3H, m), 1.24 (3H, t, *J* = 7 Hz); ^{13}C NMR (125 MHz, $CDCl_3$) δ = 172.8, 124.5, 72.9, 64.5, 60.5, 46.8, 43.1, 38.9, 36.9, 32.4, 31.6, 28.6, 21.1, 14.2; 1D NOESY NMR (500 MHz, $CDCl_3$) Irradiation of CH (δ = 3.15 ppm) led to enhancement of CH (2.76 ppm, 1.2%); irradiation of CH (δ = 2.76 ppm) led to enhancement of CH (3.15 ppm, 1.3%) and CH (2.65 ppm, 4.9%); HRMS (ES) Found: 249.1594. $C_{14}H_{21}N_2O_2$ requires MH^+, 249.1603.

6a-(*tert*-Butyldimethylsilyloxymethyl)decahydrocyclopenta[*hi*]indolizidine (14): Aldehyde **8a** (1.37 g, 4.3 mmol) and glycine (1.29 g, 17.2 mmol) were dissolved in PhMe (45 mL) and heated under reflux. After 16 h, the solvent was evaporated and the residue was adsorbed onto silica. Purification by column chromatography, eluting with CH_2Cl_2–MeOH/NH_3 (49:1) gave cycloadduct **14** (0.65 g, 51%) as an oil; R_f 0.3 [CH_2Cl_2–MeOH/NH_3 (49:1)]; v_{max}/cm^{-1} 2955, 1470, 1265, 1100; ^1H NMR (400 MHz, $CDCl_3$) δ = 3.24 (2H, s) 3.05–2.93 (2H, m), 2.64–2.35 (4H, m), 2.10–1.97 (2H, m), 1.82–1.32 (8H, m) 0.91 (9H, s), 0.04 (6H, s); ^{13}C NMR (100 MHz, $CDCl_3$) δ = 70.0, 69.3, 52.7, 48.6, 47.2, 41.3, 33.5, 33.2, 31.0, 28.3, 25.9, 19.2, 18.3, −5.5; HRMS (ES) Found: MH^+, 296.2410. $C_{17}H_{34}NOSi$ requires MH^+, 296.2415.

(Decahydrocyclopenta[*hi*]indolizin-6a-yl)methanol (15): TBAF (3.40 mL, 3.4 mmol, 1.0 M solution in THF) was added dropwise to amine **14** (0.41 g, 1.35 mmol) in THF (14 mL) with 4 Å molecular sieves at room temperature. After 36 h, the mixture was filtered and the solvent was evaporated. Purification by Isolute® SCX solid-phase cartridge gave alcohol **15** (0.15 g, 62%) as an oil which crystallized on standing as needles; mp 80–83 °C; R_f 0.05 [CH_2Cl_2–MeOH/NH_3 (9:1)]; ν_{max}/cm^{-1} 3335, 2930, 2860, 1455; ^1H NMR (500 MHz, $CDCl_3$) δ = 3.11 (2H, s), 2.87–2.79 (2H, m), 2.39–2.32 (1H, m), 2.26–2.17 (3H, m), 2.01–1.83 (3H, m), 1.75–1.65 (1H, m), 1.61–1.54 (1H, m), 1.50–1.29 (5H, m); ^{13}C NMR (100MHz, $CDCl_3$) δ = 69.4, 69.1, 52.9, 48.7, 47.2, 41.8, 33.9, 33.3, 31.5, 28.6, 18.8; HRMS (ES) Found: MH^+, 182.1545. $C_{11}H_{20}NO$ requires MH^+, 182.1539. For single crystal X-ray diffraction, see Cambridge Crystallographic Data Centre, CCDC 846335.

Decahydrocyclopenta[*hi*]indolizidine-6a-carbaldehyde (16): In the same way as aldehyde **6**, oxalyl chloride (0.06 mL, 0.76 mmol), DMSO (0.11 mL, 1.6 mmol) and alcohol **15** (0.12 g, 0.66 mmol) gave, after purification by column chromatography, eluting with CH_2Cl_2–MeOH/NH_3 (95:5), aldehyde **16** (0.09 g, 76%) as an oil; R_f 0.2 [CH_2Cl_2–MeOH/NH_3 (95:5)]; ν_{max}/cm^{-1} 2930, 1725, 1455; ^1H NMR (400 MHz, $CDCl_3$) δ = 9.46 (1H, s), 3.12–3.04 (2H, m), 3.01–2.93 (1H, m), 2.67–2.50 (3H, m), 2.14–2.02 (1H, m), 1.98–1.44 (9H, m); (100MHz, $CDCl_3$) δ = 204.9, 66.8, 57.1, 52.3, 47.7, 41.4, 33.5, 33.1, 29.4, 25.1, 17.7; HRMS (ES) Found: MH^+, 180.1388. $C_{11}H_{18}NO$ requires MH^+, 180.1388.

2-(3-Chloropropyl)-2-vinylhex-5-enenitrile (17): KO*t*-Bu (0.67 g, 5.98 mmol) in THF (6 mL) was added to methyltriphenylphosphonium bromide (2.14 g, 5.98 mmol) in

THF (30 mL) at −78 °C. After 1 h, aldehyde **6** (0.99 g, 4.96 mmol) in THF (5 mL) was added dropwise. After 1 h, the mixture was allowed to warm to room temperature. After a further 1 h, the mixture was diluted with hexane and was filtered over Celite®. The filtrate was evaporated and the mixture was purified by column chromatography, eluting with petrol–EtOAc (99:1), to give nitrile **17** (0.75 g, 76%) as an oil; R_f 0.20 [petrol–EtOAc (99:1)]; v_{max}/cm^{-1} 2925, 2260, 1640, 1450; ^{1}H NMR (400 MHz, $CDCl_3$) δ = 5.79 (1H, ddt, *J* = 17, 10, 6.5 Hz), 5.58–5.43 (2H, m), 5.37 (1H dd *J* = 9.5, 1.5 Hz), 5.12–5.00 (2H, m), 3.66–3.54 (2H, m), 2.31–2.12 (2H, m), 2.05–1.57 (6H, m); ^{13}C NMR (100 MHz, $CDCl_3$) δ = 136.7, 136.2, 120.7, 117.9, 115.7, 45.6, 44.4, 38.1, 36.2, 29.2, 28.1; HRMS (ES) Found: M$^+$, 197.0971. $C_{11}H_{16}N^{35}Cl$ requires M$^+$, 197.0962.

2-(3-Chloropropyl)-2-vinylhex-5-enal (18): In the same way as aldehyde **8b**, nitrile **17** (0.37 g, 1.87 mmol) and DIBAL-H (2.43 mL, 2.43 mmol, 1.0 M in hexanes) gave, after purification by column chromatography, eluting with petrol–EtOAc (99:1), aldehyde **18** (0.30 g, 80%) as an oil; R_f 0.50 [petrol–EtOAc (19:1)]; v_{max}/cm^{-1} 2935, 1725, 1640, 1460; ^{1}H NMR (400 MHz, $CDCl_3$) δ = ^{1}H NMR (400 MHz, $CDCl_3$) δ = 9.42 (1H, s), 5.87–5.71 (1H, m), 5.75 (1H, dd, *J* = 18, 11 Hz), 5.40 (1H, dd, *J* = 11, 0.5 Hz), 5.19 (1H, dd, *J* = 18, 0.5 Hz) 5.09–4.97 (2H, m), 3.56 (2H, t, *J* = 6 Hz), 2.08–1.91 (2H, m), 1.86–1.66 (6H, m); ^{13}C NMR (100 MHz, $CDCl_3$) δ = 202.4, 137.8, 137.2, 118.1, 115.2, 55.3, 45.2, 32.0, 29.7, 28.0, 27.0; HRMS (ES) Found: MH$^+$, 201.1046, $C_{11}H_{18}O^{35}Cl$ requires MH$^+$, 201.1041.

6a-Vinyldecahydrocyclopenta[*hi*]indolizine (19): Aldehyde **18** (0.30 g, 1.49 mmol) and glycine (0.45 g, 6.00 mmol) in toluene (15 mL) were heated under reflux. After 16 h, the solvent was evaporated and the mixture adsorbed onto silica. Purification

by column chromatography, eluting with CH_2Cl_2–MeOH/NH_3 (99:1), gave alkene **19** (0.16 g, 60%) as an oil; R_f 0.10 [CH_2Cl_2–MeOH/NH_3 (99:1)]; ν_{max}/cm^{-1} 2935, 2775, 1635, 1460; ^{1}H NMR (500 MHz, $CDCl_3$) δ =5.70 (1H, dd, *J* = 17.5, 11 Hz), 5.00–4.94 (2H, m), 3.07–3.02 (1H, m), 3.01–2.95 (1H, m), 2.79 (1H, brs), 2.69–2.49 (3H, m), 2.16–1.97 (2H, m), 1.96–1.86 (1H, m), 1.83–1.62 (2H, m), 1.63–1.54 (1H, m), 1.53–1.34 (4H, m); (100 MHz, $CDCl_3$) δ =146.6, 110.7, 72.9, 53.0, 48.7, 47.8, 40.8, 33.3, 33.2, 32.3, 31.3, 20.0; HRMS (ES) Found: MH$^+$, 178.1596. $C_{12}H_{20}N$ requires MH$^+$ 178.1604.

6a-(*tert*-Butyldimethylsilyloxymethyl)decahydrocyclopenta[*hi*]indolizine-2-carboxylic acid ethyl ester (20): Aldehyde **8a** (1.39 g, 4.36 mmol), glycine ethyl ester hydrochloride (0.91 g, 6.55 mmol) and *N*,*N*-diisopropylethylamine (2.28 mL, 13.1 mmol) in PhMe (45 mL) were heated under reflux. After 2 h, the solvent was evaporated and the residue was adsorbed onto silica. Purification by column chromatography, eluting with petrol–EtOAc (19:1) gave cycloadduct **20** (1.32 g, 83%) as an oil; R_f 0.3 [petrol–EtOAc (19:1)]; ν_{max}/cm^{-1} 2935, 2855, 1730, 1460, 1250; ^{1}H NMR (500 MHz, $CDCl_3$) δ = 4.17 (1H, dq, *J* = 8.5, 3.5 Hz), 4.14 (1H, dq, *J* = 8.5, 3.5 Hz), 3.73 (1H, dd, *J* = 8, 6.5 Hz), 3.25 (1H, d, *J* = 3 Hz), 3.18 (2H, s), 2.95–2.88 (1H, m), 2.75–2.65 (2H, m), 2.12–2.04 (1H, m), 2.04–1.96 (1H, m), 1.85 (1H, dd, *J* = 11.5, 6.5 Hz), 1.69–1.58 (3H, m), 1.54–1.47 (1H, m), 1.40–1.33 (2H, m), 1.30–1.23 (4H, m), 0.87 (9H, s), 0.00 (6H, s); ^{13}C NMR (125 MHz, $CDCl_3$) δ = 174.1, 69.4, 67.5, 63.4, 60.3, 47.0, 45.9, 41.3, 39.2, 32.8, 31.2, 28.9, 25.9, 18.3, 17.5, 14.4, −5.5; HRMS (ES) Found: MH$^+$, 368.2624. $C_{20}H_{38}NO_3Si$ requires MH$^+$, 368.2621.

6a-(*tert*-butyldimethylsilanyloxymethyl)decahydropyrrolo[3,2,1-*ij*]quinoline-2-carboxylic acid ethyl ester (21): In the same way as cycloadduct **20**, aldehyde **8b**

(593 mg, 1.78 mmol), glycine ethyl ester hydrochloride (314 mg, 2.23 mmol) and *N*,*N*-diisopropylethylamine (0.78 mL, 4.45 mmol) gave, after heating for 2 h and purification by column chromatography, eluting with petrol–EtOAc (19:1), cycloadduct **23** (78 mg, 11%) as an oil. Further elution with petrol–EtOAc–EtOH (75:22:3) gave cycloadduct **22** (61 mg, 9%) as an oil and cycloadduct **21** (422 mg, 62%) as an oil. Data for **21**: R_f 0.26 [petrol–EtOAc–EtOH (75:22:3)]; ν_{max}/cm^{-1} 2930, 2855, 1735, 1185, 1090; ^{1}H NMR (500 MHz, $CDCl_3$) δ = 4.17–4.07 (2H, m), 4.05 (1H, d, *J* = 9.5 Hz), 3.12 (1H, d, *J* = 9.5 Hz), 3.26 (1H, dd, *J* = 9, 6.5 Hz), 2.73–2.68 (1H, m), 2.42–2.29 (2H, m), 2.20 (1H, d, *J* = 11.5 Hz), 2.07–1.97 (1H, m), 1.95–1.82 (2H, m), 1.69–1.63 (1H, m), 1.57 (1H, dquin, *J* = 13, 4 Hz), 1.51–1.25 (5H, m), 1.23 (3H, t, *J* = 7 Hz), 1.16 (1H, td, *J* = 14, 4 Hz), 0.91 (1H, qd, *J* = 12, 3 Hz), 0.87 (9H, s), 0.02 (6H, s); ^{13}C NMR (125 MHz, $CDCl_3$) δ = 174.8, 69.4, 67.4, 66.0, 60.4, 50.1, 38.2, 34.5 (2 × CH_2), 34.3, 30.0, 25.9, 22.5, 22.4, 21.9, 18.2, 14.2, −5.4, −5.5; HRMS (ES) Found: MH^+, 382.2772. $C_{21}H_{40}NO_3Si$ requires MH^+, 382.2777.

6a-Hydroxymethyldecahydrocyclopenta[*hi*]indolizine-2-carboxylic acid ethyl ester (24): TBAF (9.0 mL, 9.0 mmol, 1.0 M solution in THF) was added to silyl ether **20** (1.32 g, 3.60 mmol) in THF (36 mL) with 4 Å molecular sieves. After 48 h, the mixture was filtered and the solvent was evaporated. The mixture was adsorbed onto silica. Purification by column chromatography, eluting with CH_2Cl_2–MeOH (99:1), gave alcohol **24** (1.32 g, 74%) as an oil; R_f 0.3 [CH_2Cl_2–MeOH (99:1)]; ν_{max}/cm^{-1} 3410, 2935, 2860, 1730, 1460; ^{1}H NMR (500 MHz, $CDCl_3$) δ = 4.20–4.10 (2H, m), 3.75 (1H, t, *J* = 7 Hz), 3.29–3.27 (2H, m), 3.15 (1H, d, *J* = 6.5 Hz), 2.96–2.90 (1H, m), 2.74–2.65 (2H, m), 2.13–1.98 (2H, m), 1.86 (1H, ddd, *J* = 12.5, 7, 1.5 Hz), 1.76–1.60 (3H, m), 1.57–1.31 (4H, m), 1.25 (3H, t, *J* = 7 Hz); ^{13}C NMR (100 MHz, $CDCl_3$)

δ = 174.0, 69.2, 67.6, 63.7, 60.3, 47.0, 45.8, 41.0, 38.7, 32.5, 30.4, 28.4, 18.2, 14.4; HRMS (ES) Found: MH^+, 254.1750. $C_{14}H_{24}NO_3$ requires MH^+, 254.1756.

6a-Formyldecahydrocyclopenta[*hi*]indolizine-2-carboxylic acid ethyl ester (25): In the same way as aldehyde **6**, oxalyl chloride (0.09 mL, 1.02 mmol), DMSO (0.15 mL, 2.14 mmol) and alcohol **24** (0.23 g, 0.89 mmol) gave, after purification by column chromatography, eluting with CH_2Cl_2–MeOH (99.5:0.5), aldehyde **25** (0.18 g, 81%) as an oil; R_f 0.20 [CH_2Cl_2–MeOH (99.5:0.5)]; ν_{max}/cm^{-1} 2940, 2860, 1720, 1450; ^1H NMR (500 MHz, $CDCl_3$) δ = 9.41 (1H, s), 4.20–4.10 (2H, m), 3.76–3.69 (2H, m), 3.02–2.94 (1H, m), 2.83–2.73 (1H, m), 2.72–2.65 (1H, m), 2.14 (1H, dt, *J* = 12, 8.5 Hz), 1.90–1.63 (7H, m), 1.49–1.36 (2H, m) 1.25 (3H, t, *J* = 7 Hz); ^{13}C NMR (125 MHz, $CDCl_3$) δ = 204.5, 173.6, 65.2, 63.4, 60.5, 57.1, 45.3, 41.0, 38.7, 32.8, 29.3, 25.3, 16.8, 14.3; HRMS (ES) Found: MH^+, 252.1593. $C_{14}H_{22}NO_3$ requires MH^+, 252.1600.

Decahydro-cyclopenta[*hi*]indolizine-2-carboxylic acid ethyl ester (26): Aldehyde **25** (0.10 g, 0.40 mmol) and [$RhCl(PPh_3)_3$] (0.55 g, 0.60 mmol) in PhCN (8 mL) were heated to 150 °C. After 2 h, the mixture was allowed to cool and was purified by column chromatography, eluting with petrol–EtOAc (3:1). Further purification by Isolute® SCX solid-phase cartridge gave amine **26** (0.05 g, 56%) as an oil; R_f 0.20 [petrol–EtOAc (3:1)]; ν_{max}/cm^{-1} 2935, 2865, 1735, 1445; ^1H NMR (500 MHz, $CDCl_3$) δ = 4.21–4.10 (2H, m), 3.73 (1H, dd, *J* = 9, 6.5 Hz), 3.61 (1H, t, *J* = 6.5 Hz), 2.98–2.91 (1H, m), 2.88–2.80 (1H, m), 2.72–2.66 (1H, m), 2.19–2.10 (1H, m), 1.83 (1H, ddd, *J* = 12.5, 6.5, 1.5 Hz), 1.80–1.60 (5H, m), 1.54–1.43 (3H, m), 1.25 (3H, t, *J* = 7 Hz), 1.19–1.13 (1H, m); ^{13}C NMR (100 MHz, $CDCl_3$) δ = 174.0, 65.3, 64.0, 60.5,

46.3, 41.4, 39.8, 39.5, 34.5, 30.3, 25.3, 16.2, 14.4; HRMS (ES) Found: MH^+, 224.1658. $C_{13}H_{22}NO_2$ requires MH^+, 224.1651.

6a-Hydroxymethyldecahydropyrrolo[3,2,1-*ij*]quinoline-2-carboxylic acid ethyl ester (27): Ester **21** (410 mg, 1.07 mmol), TBAF (2.7 mL, 2.69 mmol, 1.0 M in THF) in THF (10 mL) and 4 Å molecular sieves were heated to 50 °C. After 2 h, the mixture was allowed to cool to room temperature. After 18 h, the mixture was evaporated. Purification by column chromatography, eluting with CH_2Cl_2–MeOH–NH_3 (97:3:0.1), gave alcohol **27** (195 mg, 68%) as an oil; mp 47–50 °C; R_f 0.46 [CH_2Cl_2–MeOH (19:1)]; ν_{max}/cm^{-1} 3400, 2925, 2860, 1730, 1455; ^1H NMR (500 MHz, $CDCl_3$) δ = 4.12 (2H, q, *J* = 7 Hz), 3.67 (1H, d, *J* = 11 Hz), 3.60 (1H, d, *J* = 11 Hz), 3.31 (1H, dd, *J* = 9, 6 Hz), 3.17 (1H, br s), 2.71 (1H, br d, *J* = 11, 3.5 Hz), 2.42–2.35 (1H, m), 2.34 (1H, d, *J* = 12.0 Hz), 2.35–2.30 (1H, m), 2.14 (1H, qt, *J* = 13.5, 4.5 Hz), 2.07–1.98 (1H, m), 1.98–1.92 (1H, m), 1.65–1.56 (2H, m), 1.53–1.46 (1H, m), 1.41–1.17 (5H, m), 1.24 (3H, t, *J* = 7 Hz), 0.95 (1H, qd, *J* = 12, 3 Hz); ^{13}C NMR (125 MHz, $CDCl_3$) δ = 174.5, 73.2, 67.6, 66.0, 60.6, 49.0, 37.4, 34.1, 33.9, 33.7, 29.7, 25.3, 22.4, 22.2, 14.2; HRMS (ES) Found: MH^+, 268.1916. $C_{15}H_{26}NO_3$ requires MH^+, 268.1913.

6a-Formyldecahydropyrrolo[3,2,1-*ij*]quinoline-2-carboxylic acid ethyl ester (28): In the same way as aldehyde **6**, oxalyl chloride (0.05 mL, 0.63 mmol), DMSO (0.09 mL, 1.20 mmol) and alcohol **27** (134 mg, 0.50 mmol) gave, after purification by column chromatography, eluting with CH_2Cl_2–MeOH (49:1), aldehyde **28** (133 mg, 92%) as an oil; R_f 0.35 [CH_2Cl_2–MeOH (98:2)]; ν_{max}/cm^{-1} 2930, 2860, 1725, 1455; ^1H NMR (500 MHz, $CDCl_3$) δ = 9.52 (1H, d, *J* = 2 Hz), 4.13 (2H, q, *J* = 7 Hz), 3.33 (1H, dd, *J* = 9, 6.5 Hz), 2.79 (1H, d, *J* = 11.5 Hz), 2.74–2.69 (1H, m), 2.39 (1H, ddd, *J* = 12.5, 9, 7.5 Hz), 2.34 (1H, ddd, *J* = 13, 10.5, 3 Hz), 2.05–1.96 (2H, m), 1.85–1.80

(1H, m), 1.74–1.68 (1H, m), 1.68–1.59 (1H, m), 1.55–1.42 (3H, m), 1.39–1.27 (3H, m), 1.25 (3H, t, *J* = 7 Hz), 1.05 (1H, qd, *J* = 12.5, 3 Hz); ^{13}C NMR (125 MHz, $CDCl_3$) δ = 205.3, 174.4, 66.0, 65.7, 60.7, 49.6, 49.2, 34.0, 33.8, 29.9, 29.5, 22.9, 22.6, 22.1, 14.2; HRMS (ES) Found: MH^+, 266.1760. $C_{15}H_{24}NO_3$ requires MH^+, 266.1756.

Decahydropyrrolo[3,2,1-*ij*]quinoline-2-carboxylic acid ethyl ester (29): In the same way as ester **26**, aldehyde **28** (60 mg, 0.23 mmol) and [RhCl(PPh_3)$_3$] (314 mg, 0.34 mmol) gave, after heating for 1.75 h and purification by column chromatography, eluting with petrol–EtOAc–EtOH (5:15:1) then purification using an SCX-2 cartridge, ester **29** (37 mg, 69%) as an oil; R_f 0.21 [petrol–EtOAc–EtOH (5:13:2)]; ν_{max}/cm^{-1} 2925, 2860, 1735; ^{1}H NMR (500 MHz, $CDCl_3$) δ = 4.19–4.09 (2H, m), 3.30 (1H, dd, *J* = 9, 7 Hz), 2.81 (1H, br dt, *J* = 10.5, 3 Hz), 2.60 (1H, dd, *J* = 11.5, 5 Hz), 2.37–2.29 (2H, m), 2.29–2.22 (1H, m), 1.96–1.84 (2H, m), 1.74–1.59 (2H, m), 1.58–1.44 (3H, m), 1.43–1.24 (4H, m), 1.24 (3H, t, *J* = 7 Hz), 0.97 (1H, qd, *J* = 12, 3 Hz); ^{13}C NMR (125 MHz, $CDCl_3$) δ = 174.8, 66.5, 66.4, 60.6, 50.8, 34.9, 33.7, 33.6, 30.3, 30.2, 25.9, 23.7, 21.8, 14.2; 1D NOESY NMR (500 MHz, $CDCl_3$) Irradiation of ring junction NCH (2.60 ppm) led to enhancement (1.8%) of the new ring junction CH formed after decarbonylation (2.25 ppm); HRMS (ES) Found: MH^+, 238.1812. $C_{14}H_{24}NO_2$ requires MH^+, 238.1807.

NMR spectra

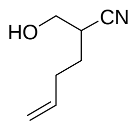

4a

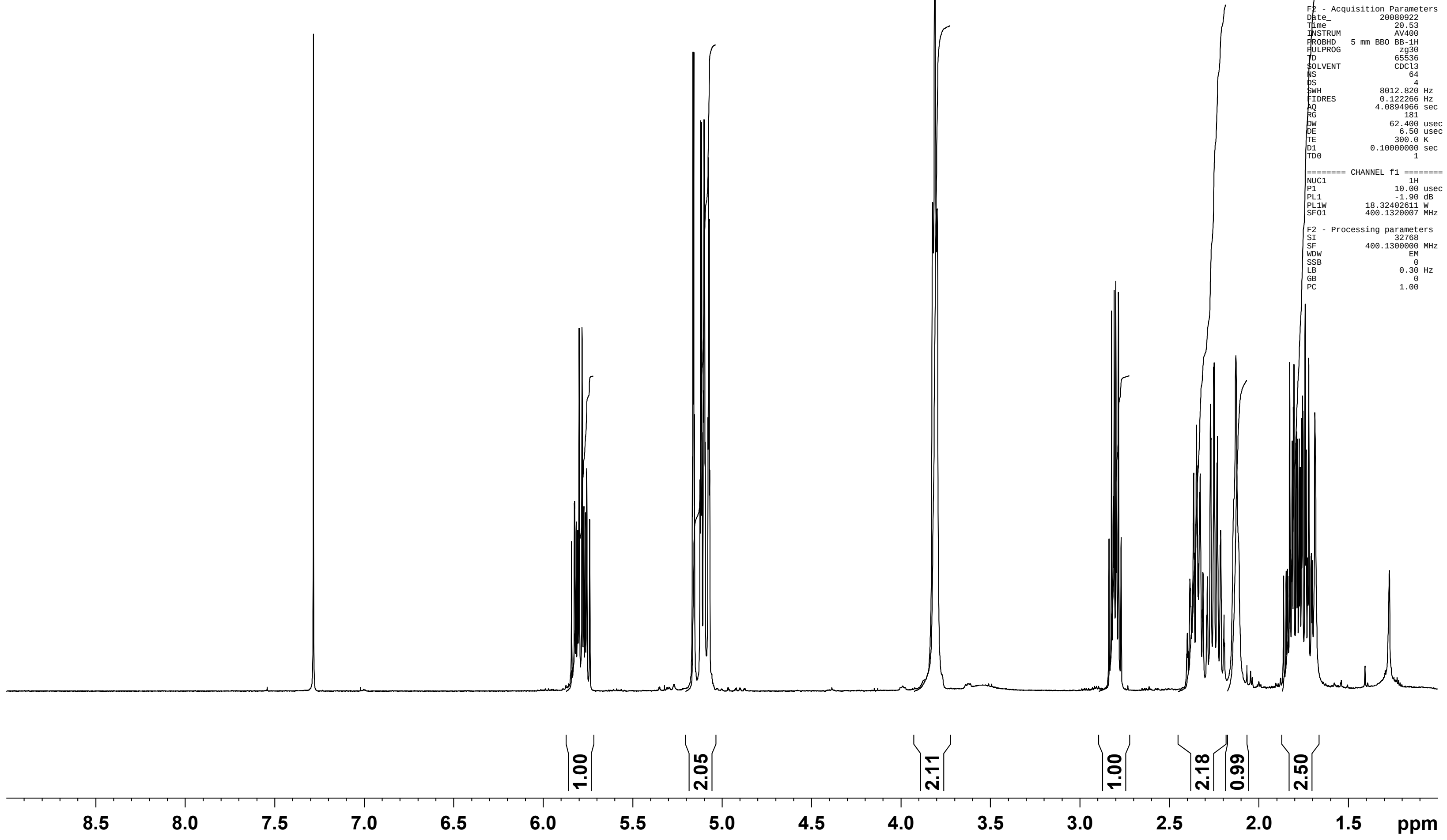

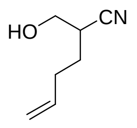

4a

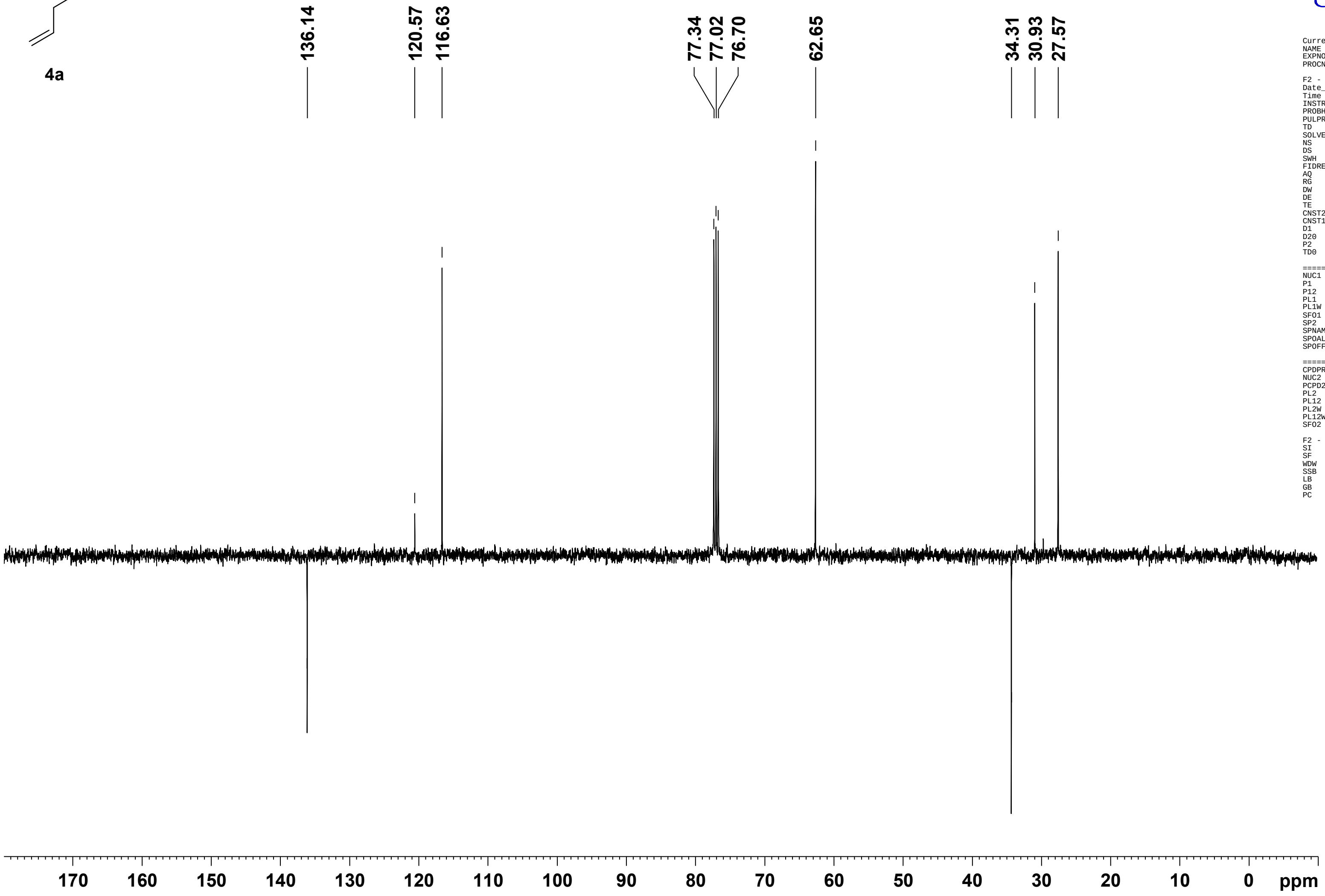


```
Current Data Parameters
NAME                  4a
EXPNO                  9
PROCNO                 1

F2 - Acquisition Parameters
Date_           20080922
Time               21.40
INSTRUM            AV400
PROBHD   5 mm BBO BB-1H
PULPROG           jmodsp
TD                 65536
SOLVENT            CDCl3
NS                   512
DS                     4
SWH            25125.629 Hz
FIDRES          0.383387 Hz
AQ             1.3042164 sec
RG                 16384
DW                19.900 usec
DE                  6.50 usec
TE                 300.0 K
CNST2        145.0000000
CNST11         1.0000000
D1            4.00000000 sec
D20           0.00689655 sec
P2                 16.00 usec
TD0                    1

======== CHANNEL f1 ========
NUC1                 13C
P1                  8.00 usec
P12              2000.00 usec
PL1                -3.10 dB
PL1W         58.97905731 W
SFO1         100.6243395 MHz
SP2                 7.00 dB
SPNAM2       Crp60comp.4
SPOAL2             0.500
SPOFFS2             0.00 Hz

======== CHANNEL f2 ========
CPDPRG2          waltz16
NUC2                  1H
PCPD2              88.00 usec
PL2                -1.90 dB
PL12               16.99 dB
PL2W         18.32402611 W
PL12W         0.23660338 W
SFO2         400.1316005 MHz

F2 - Processing parameters
SI                 32768
SF           100.6127690 MHz
WDW                   EM
SSB                    0
LB                  2.00 Hz
GB                     0
PC                  1.40
```

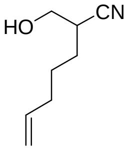

4b

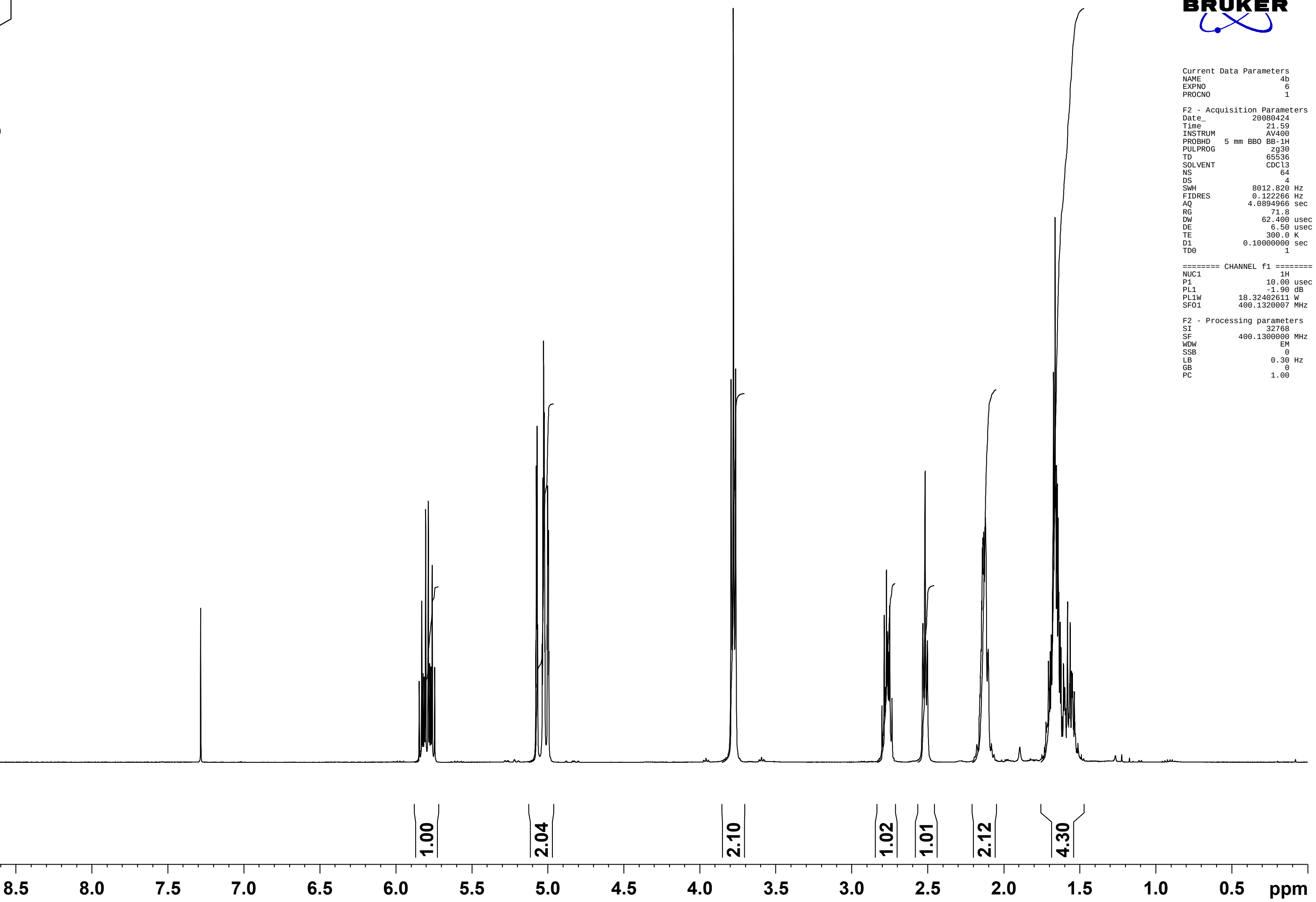


```
Current Data Parameters
NAME                4b
EXPNO                6
PROCNO               1

F2 - Acquisition Parameters
Date_          20080424
Time              21.59
INSTRUM           AV400
PROBHD   5 mm BBO BB-1H
PULPROG            zg30
TD                65536
SOLVENT           CDCl3
NS                   64
DS                    4
SWH            8012.820 Hz
FIDRES         0.122266 Hz
AQ            4.0894966 sec
RG                 71.8
DW               62.400 usec
DE                 6.50 usec
TE                300.0 K
D1           0.10000000 sec
TD0                   1

======== CHANNEL f1 ========
NUC1                 1H
P1                10.00 usec
PL1               -1.90 dB
PL1W        18.32402611 W
SFO1        400.1320007 MHz

F2 - Processing parameters
SI                32768
SF          400.1300000 MHz
WDW                  EM
SSB                   0
LB                 0.30 Hz
GB                    0
PC                 1.00
```

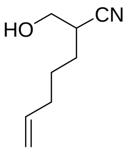

4b

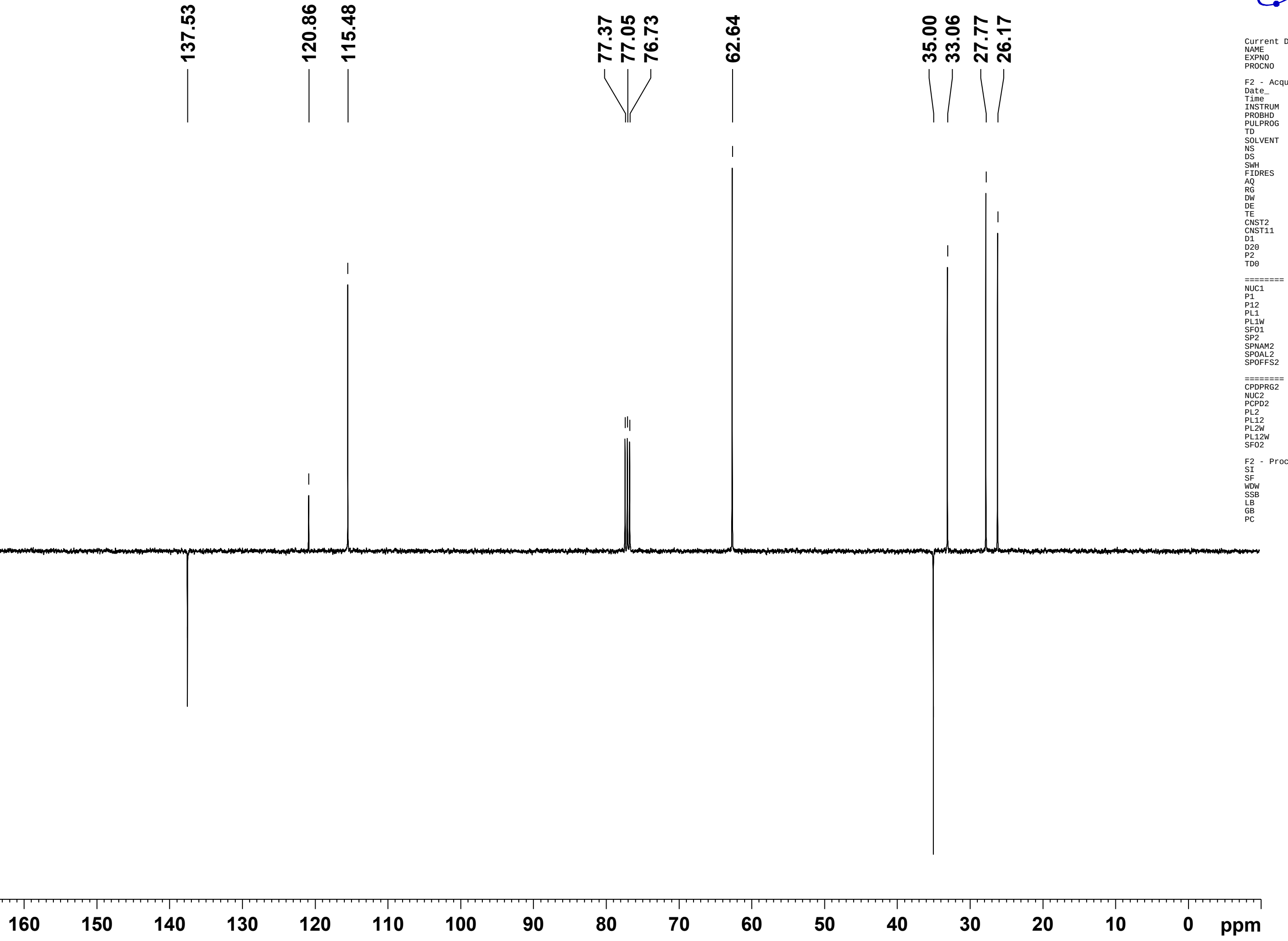


```
Current Data Parameters
NAME                4b
EXPNO                7
PROCNO               1

F2 - Acquisition Parameters
Date_         20080424
Time             22.47
INSTRUM          AV400
PROBHD   5 mm BBO BB-1H
PULPROG         jmodsp
TD               65536
SOLVENT          CDCl3
NS                 512
DS                   4
SWH          25125.629 Hz
FIDRES        0.383387 Hz
AQ           1.3042164 sec
RG               16384
DW              19.900 usec
DE                6.50 usec
TE               300.0 K
CNST2      145.0000000
CNST11       1.0000000
D1          4.00000000 sec
D20         0.00689655 sec
P2               16.00 usec
TD0                  1

======== CHANNEL f1 ========
NUC1               13C
P1                8.00 usec
P12            2000.00 usec
PL1              -3.10 dB
PL1W       58.97905731 W
SFO1       100.6243395 MHz
SP2               7.00 dB
SPNAM2     Crp60comp.4
SPOAL2           0.500
SPOFFS2           0.00 Hz

======== CHANNEL f2 ========
CPDPRG2        waltz16
NUC2                1H
PCPD2            88.00 usec
PL2              -1.90 dB
PL12             16.99 dB
PL2W       18.32402611 W
PL12W       0.23660338 W
SFO2       400.1316005 MHz

F2 - Processing parameters
SI               32768
SF         100.6127690 MHz
WDW                 EM
SSB                  0
LB                2.00 Hz
GB                   0
PC                1.40
```

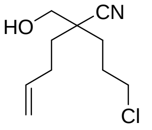

5a

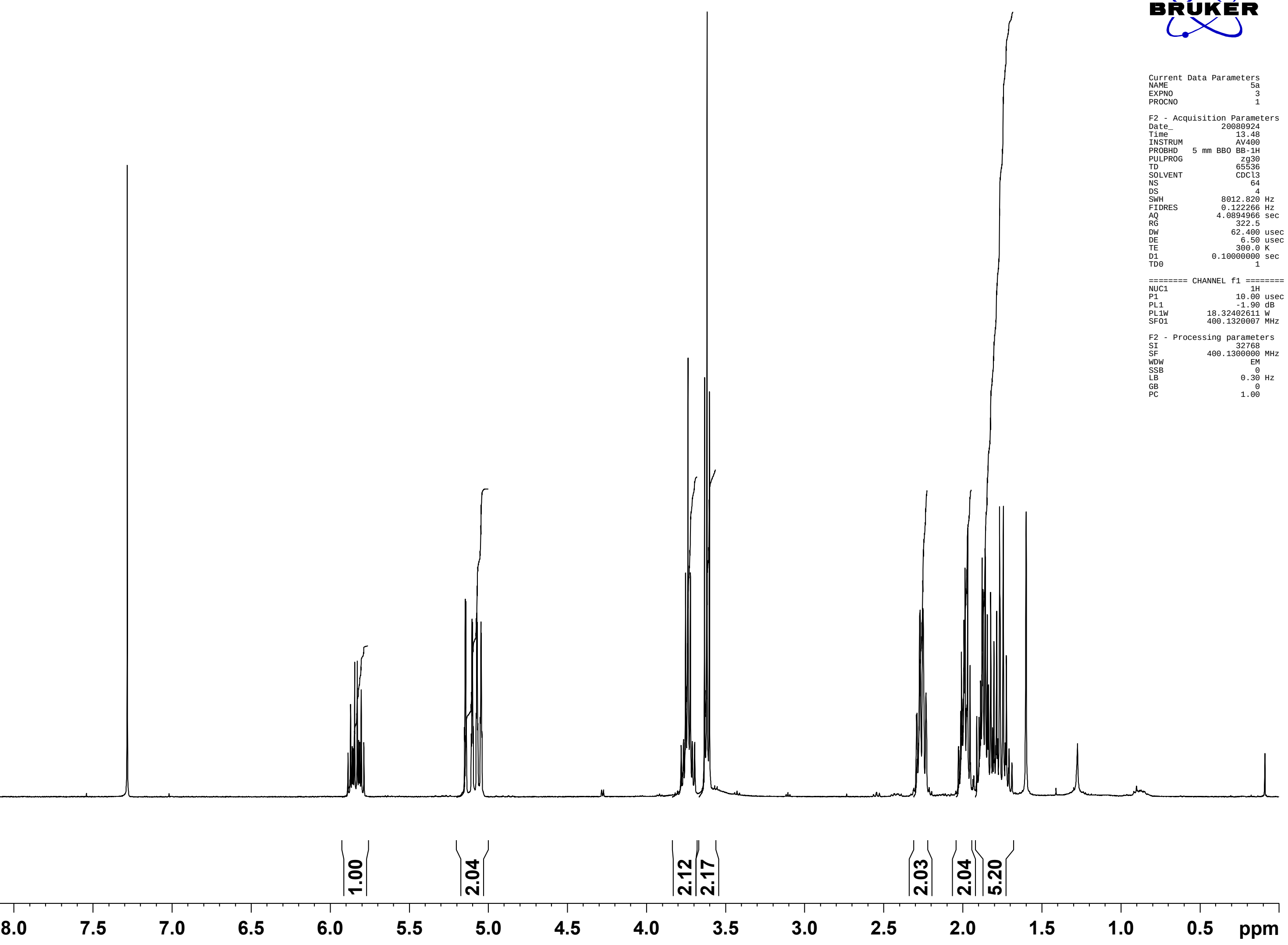

1.00
2.04
2.12
2.17
2.03
2.04
5.20
8.5 8.0 7.5 7.0 6.5 6.0 5.5 5.0 4.5 4.0 3.5 3.0 2.5 2.0 1.5 1.0 0.5 ppm
Current Data Parameters
NAME 5a
EXPNO 3
PROCNO 1
F2 - Acquisition Parameters
Date_ 20080924
Time 13.48
INSTRUM AV400
PROBHD 5 mm BBO BB-1H
PULPROG zg30
TD 65536
SOLVENT CDCl3
NS 64
DS 4
SWH 8012.820 Hz
FIDRES 0.122266 Hz
AQ 4.0894966 sec
RG 322.5
DW 62.400 usec
DE 6.50 usec
TE 300.0 K
D1 0.10000000 sec
TD0 1
======== CHANNEL f1 ========
NUC1 1H
P1 10.00 usec
PL1 -1.90 dB
PL1W 18.32402611 W
SFO1 400.1320007 MHz
F2 - Processing parameters
SI 32768
SF 400.1300000 MHz
WDW EM
SSB 0
LB 0.30 Hz
GB 0
PC 1.00

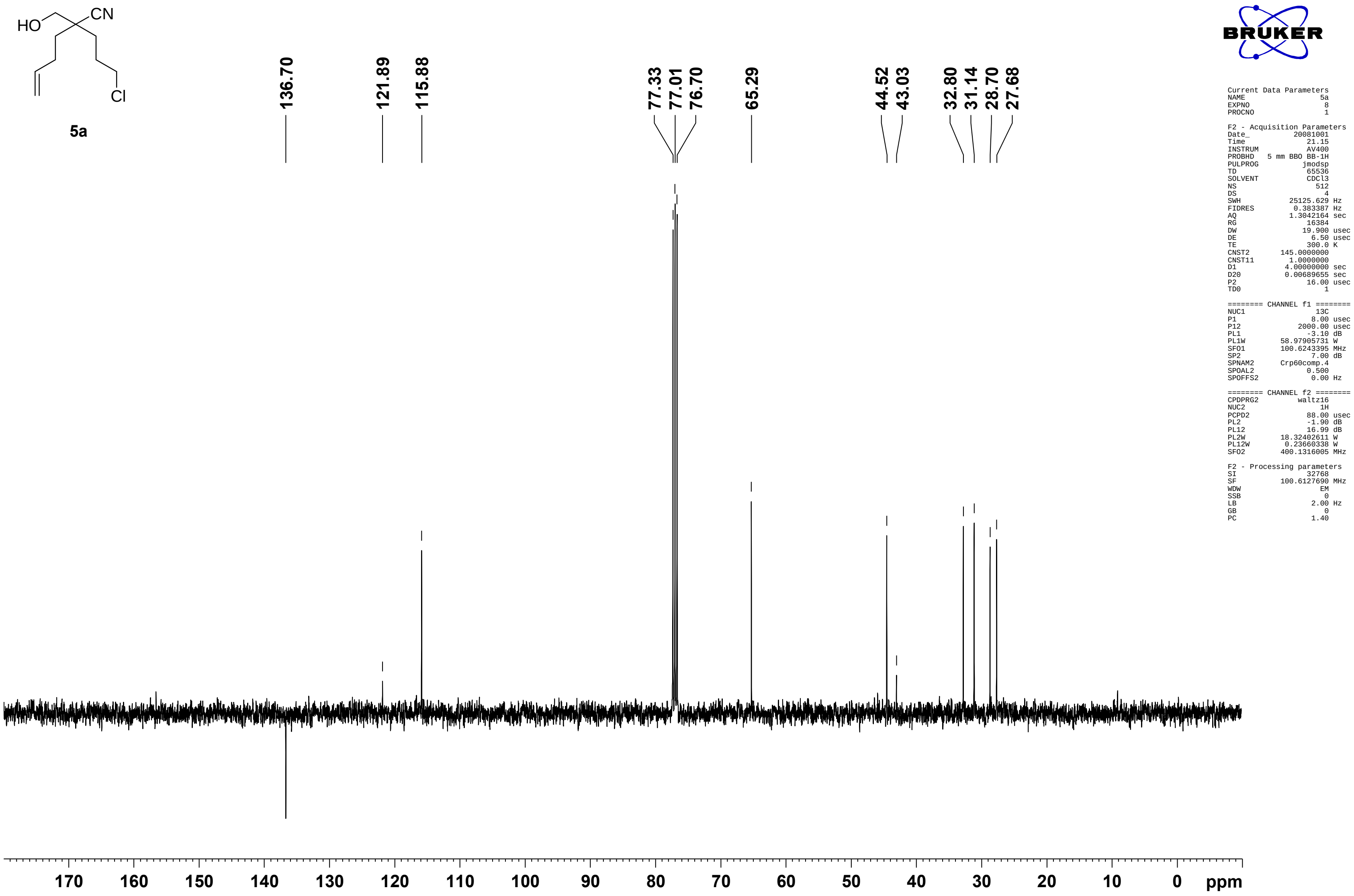
HO
CN
Cl
136.70
121.89
115.88
77.33
77.01
76.70
65.29
44.52
43.03
32.80
31.14
28.70
27.68
170 160 150 140 130 120 110 100 90 80 70 60 50 40 30 20 10 0 ppm
BRUKER
Current Data Parameters
NAME 5a
EXPNO 8
PROCNO 1
F2 - Acquisition Parameters
Date_ 20081001
Time 21.15
INSTRUM AV400
PROBHD 5 mm BBO BB-1H
PULPROG jmodsp
TD 65536
SOLVENT CDCl3
NS 512
DS 4
SWH 25125.629 Hz
FIDRES 0.383387 Hz
AQ 1.3042164 sec
RG 16384
DW 19.900 usec
DE 6.50 usec
TE 300.0 K
CNST2 145.0000000
CNST11 1.0000000
D1 4.00000000 sec
D20 0.00689655 sec
P2 16.00 usec
TD0 1
======== CHANNEL f1 ========
NUC1 13C
P1 8.00 usec
P12 2000.00 usec
PL1 -3.10 dB
PL1W 58.97905731 W
SFO1 100.6243395 MHz
SP2 7.00 dB
SPNAM2 Crp60comp.4
SPOAL2 0.500
SPOFFS2 0.00 Hz
======== CHANNEL f2 ========
CPDPRG2 waltz16
NUC2 1H
PCPD2 88.00 usec
PL2 -1.90 dB
PL12 16.99 dB
PL2W 18.32402611 W
PL12W 0.23660338 W
SFO2 400.1316005 MHz
F2 - Processing parameters
SI 32768
SF 100.6127690 MHz
WDW EM
SSB 0
LB 2.00 Hz
GB 0
PC 1.40

5a

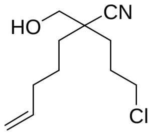
HO
CN
Cl

5b

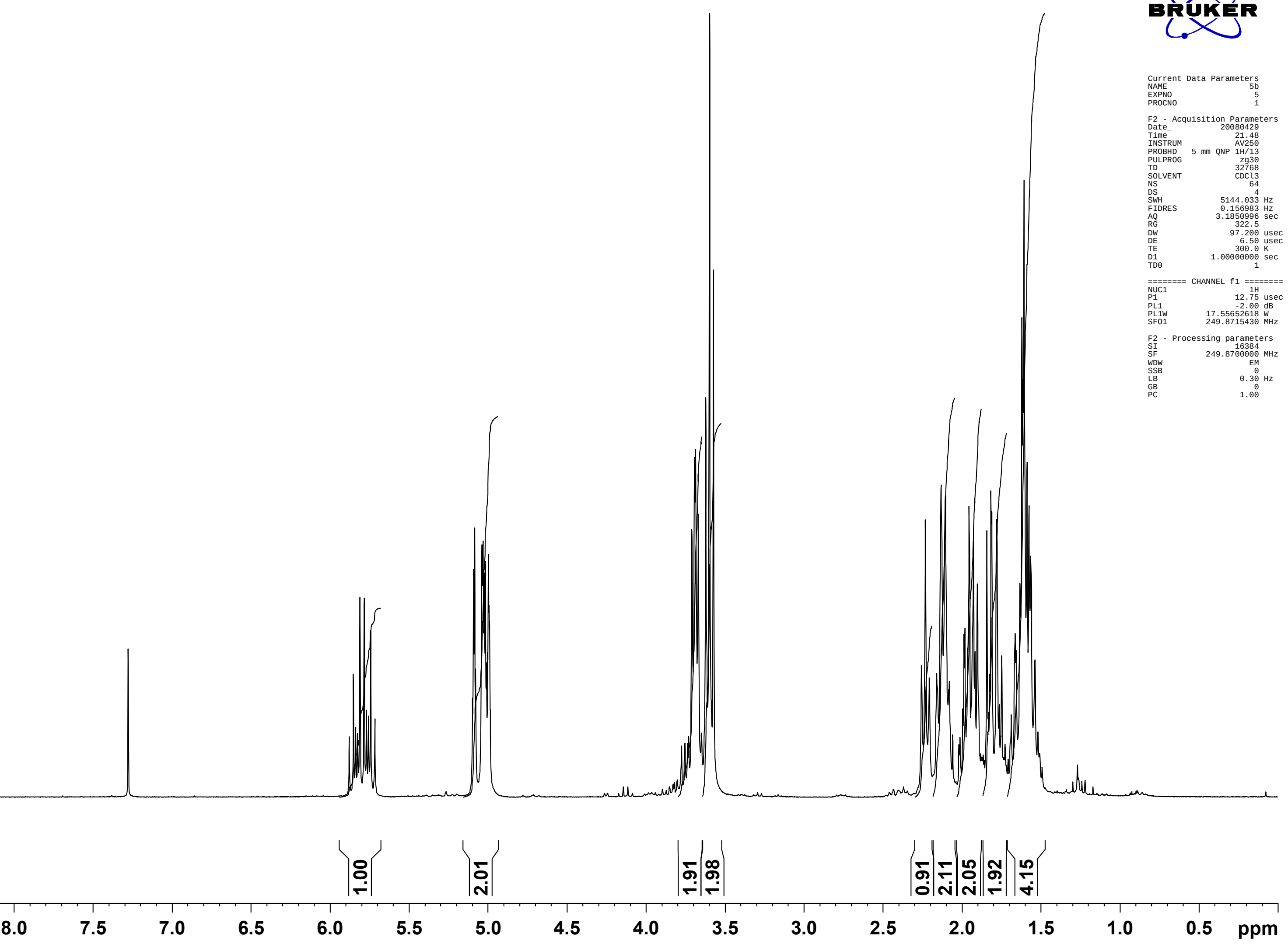
Current Data Parameters
NAME 5b
EXPNO 5
PROCNO 1
F2 - Acquisition Parameters
Date_ 20080429
Time 21.48
INSTRUM AV250
PROBHD 5 mm QNP 1H/13
PULPROG zg30
TD 32768
SOLVENT CDCl3
NS 64
DS 4
SWH 5144.033 Hz
FIDRES 0.156983 Hz
AQ 3.1850996 sec
RG 322.5
DW 97.200 usec
DE 6.50 usec
TE 300.0 K
D1 1.00000000 sec
TD0 1
======== CHANNEL f1 ========
NUC1 1H
P1 12.75 usec
PL1 -2.00 dB
PL1W 17.55652618 W
SFO1 249.8715430 MHz
F2 - Processing parameters
SI 16384
SF 249.8700000 MHz
WDW EM
SSB 0
LB 0.30 Hz
GB 0
PC 1.00
1.00
2.01
1.91
1.98
0.91
2.11
2.05
1.92
4.15
8.5
8.0
7.5
7.0
6.5
6.0
5.5
5.0
4.5
4.0
3.5
3.0
2.5
2.0
1.5
1.0
0.5
ppm

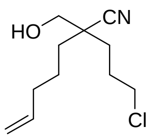

5b

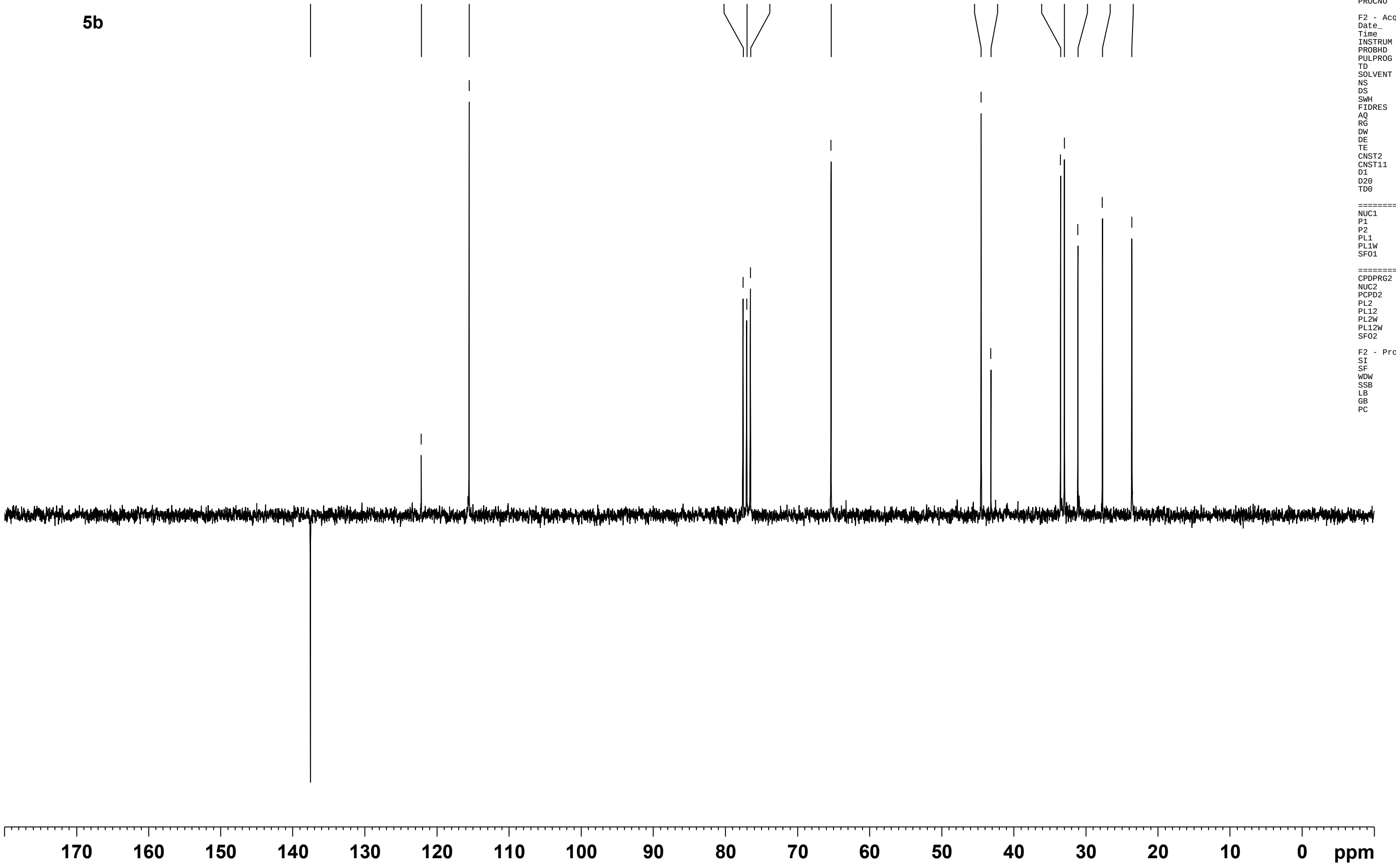


```
Current Data Parameters
NAME                 5b
EXPNO                 6
PROCNO                1

F2 - Acquisition Parameters
Date_          20080429
Time              22.32
INSTRUM           AV250
PROBHD   5 mm QNP 1H/13
PULPROG            jmod
TD                32768
SOLVENT           CDCl3
NS                  512
DS                    4
SWH           15723.271 Hz
FIDRES         0.479836 Hz
AQ            1.0420724 sec
RG                16384
DW               31.800 usec
DE                 6.50 usec
TE                300.0 K
CNST2       145.0000000
CNST11        1.0000000
D1           4.00000000 sec
D20          0.00689655 sec
TD0                   1

======== CHANNEL f1 ========
NUC1                13C
P1                 9.00 usec
P2                18.00 usec
PL1                0.00 dB
PL1W        37.17591858 W
SFO1         62.8370864 MHz

======== CHANNEL f2 ========
CPDPRG2         waltz16
NUC2                 1H
PCPD2             80.00 usec
PL2               -2.00 dB
PL12              13.95 dB
PL2W        17.55652618 W
PL12W        0.44610655 W
SFO2        249.8709995 MHz

F2 - Processing parameters
SI                16384
SF           62.8298610 MHz
WDW                  EM
SSB                   0
LB                 1.00 Hz
GB                    0
PC                 1.40
```

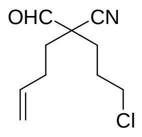
OHC
CN
Cl

6

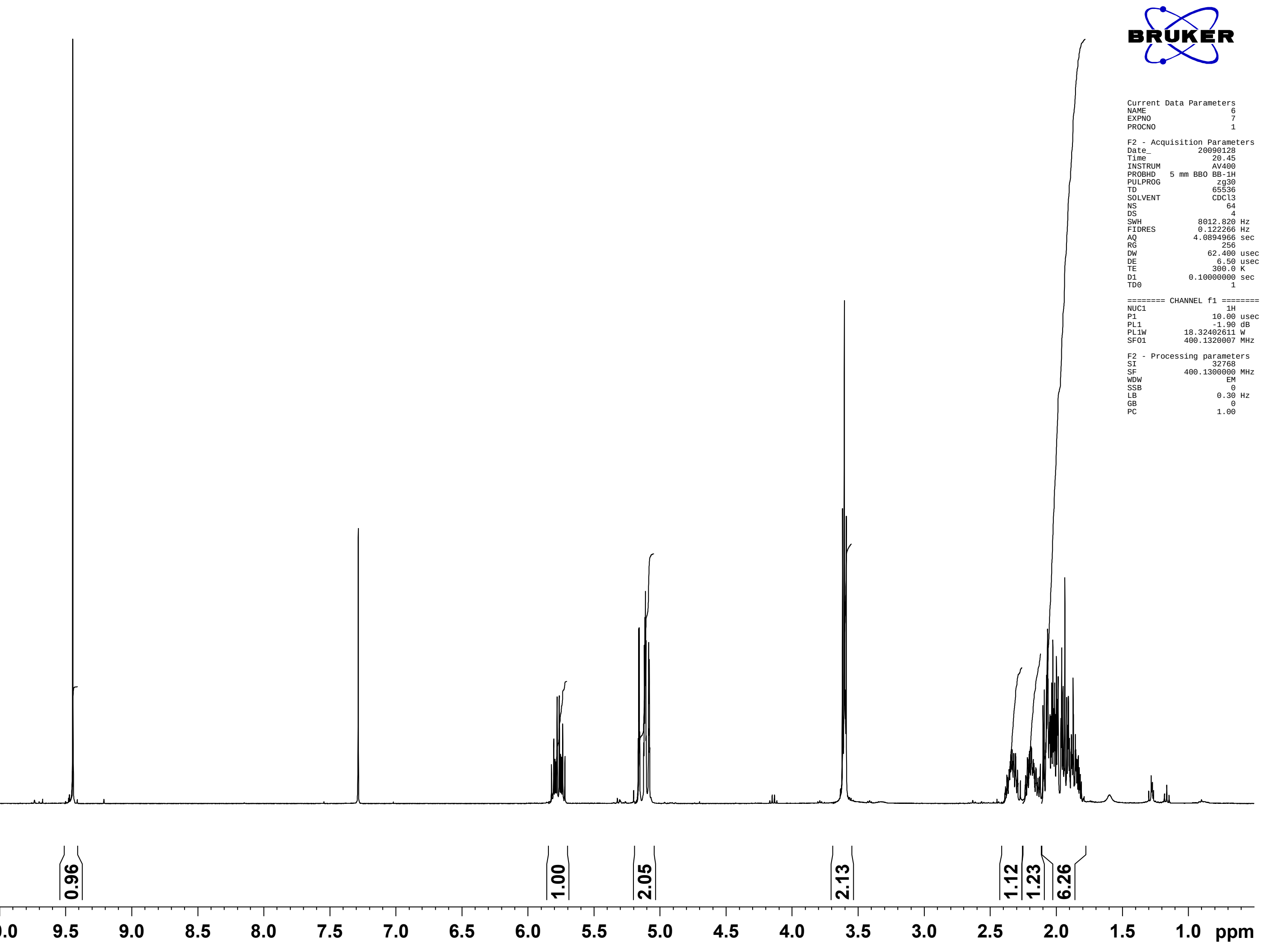
Current Data Parameters
NAME 6
EXPNO 7
PROCNO 1
F2 - Acquisition Parameters
Date_ 20090128
Time 20.45
INSTRUM AV400
PROBHD 5 mm BBO BB-1H
PULPROG zg30
TD 65536
SOLVENT CDCl3
NS 64
DS 4
SWH 8012.820 Hz
FIDRES 0.122266 Hz
AQ 4.0894966 sec
RG 256
DW 62.400 usec
DE 6.50 usec
TE 300.0 K
D1 0.10000000 sec
TD0 1
======== CHANNEL f1 ========
NUC1 1H
P1 10.00 usec
PL1 -1.90 dB
PL1W 18.32402611 W
SFO1 400.1320007 MHz
F2 - Processing parameters
SI 32768
SF 400.1300000 MHz
WDW EM
SSB 0
LB 0.30 Hz
GB 0
PC 1.00
0.96
1.00
2.05
2.13
1.12
1.23
6.26
10.5 10.0 9.5 9.0 8.5 8.0 7.5 7.0 6.5 6.0 5.5 5.0 4.5 4.0 3.5 3.0 2.5 2.0 1.5 1.0 ppm

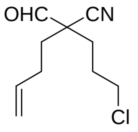

6

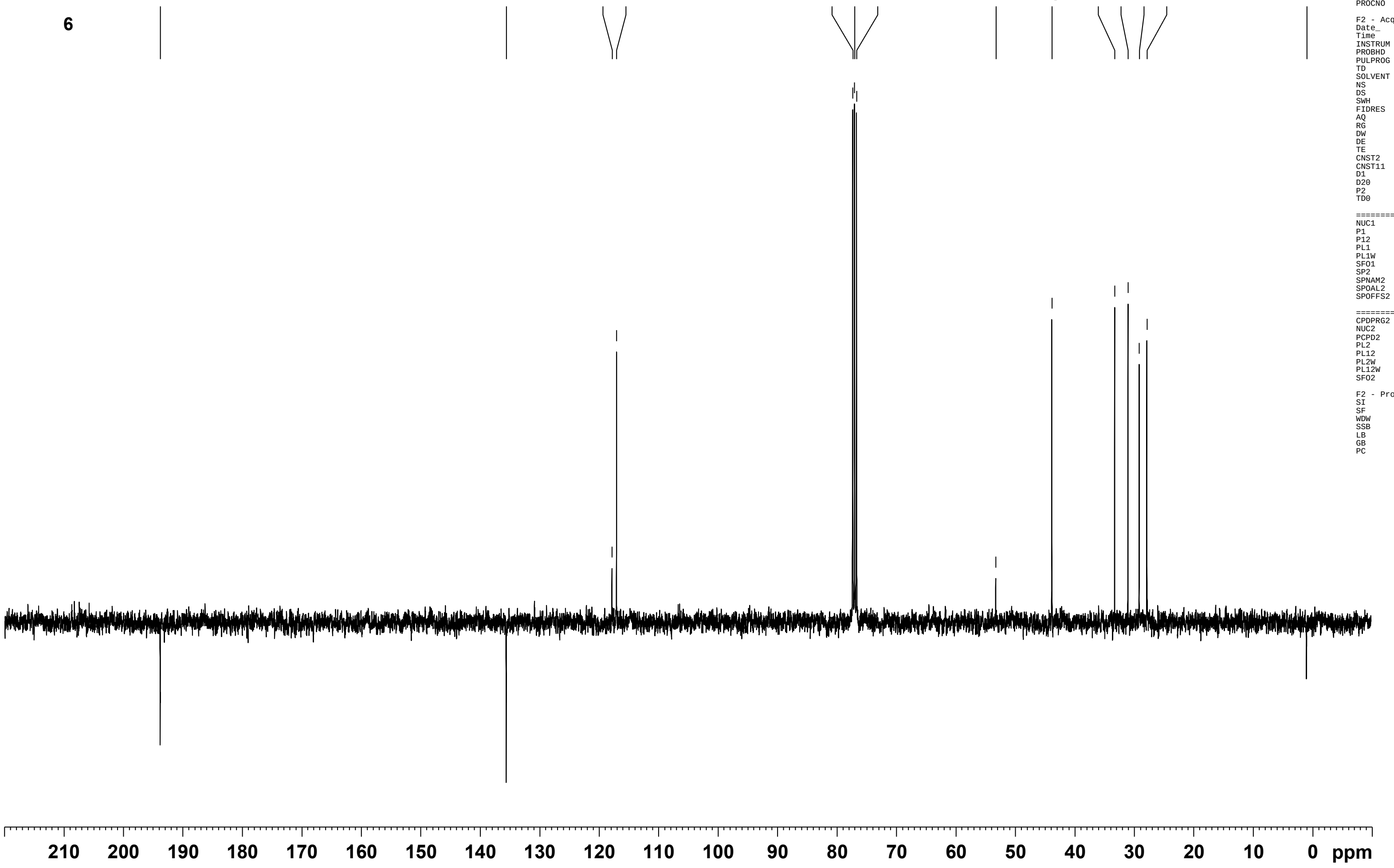


```
Current Data Parameters
NAME                  6
EXPNO                 8
PROCNO                1

F2 - Acquisition Parameters
Date_          20090128
Time              21.32
INSTRUM           AV400
PROBHD   5 mm BBO BB-1H
PULPROG          jmodsp
TD                65536
SOLVENT           CDCl3
NS                  512
DS                    4
SWH           25125.629 Hz
FIDRES         0.383387 Hz
AQ            1.3042164 sec
RG                16384
DW               19.900 usec
DE                 6.50 usec
TE                300.0 K
CNST2       145.0000000
CNST11        1.0000000
D1           4.00000000 sec
D20          0.00689655 sec
P2                16.00 usec
TD0                   1

======== CHANNEL f1 ========
NUC1                13C
P1                 8.00 usec
P12             2000.00 usec
PL1               -3.10 dB
PL1W        58.97905731 W
SFO1        100.6243395 MHz
SP2                7.00 dB
SPNAM2      Crp60comp.4
SPOAL2            0.500
SPOFFS2            0.00 Hz

======== CHANNEL f2 ========
CPDPRG2         waltz16
NUC2                 1H
PCPD2             88.00 usec
PL2               -1.90 dB
PL12              16.99 dB
PL2W        18.32402611 W
PL12W        0.23660338 W
SFO2        400.1316005 MHz

F2 - Processing parameters
SI                32768
SF          100.6127690 MHz
WDW                  EM
SSB                   0
LB                 2.00 Hz
GB                    0
PC                 1.40
```

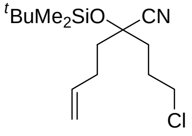

$^tBuMe_2SiO$
CN
Cl

7a

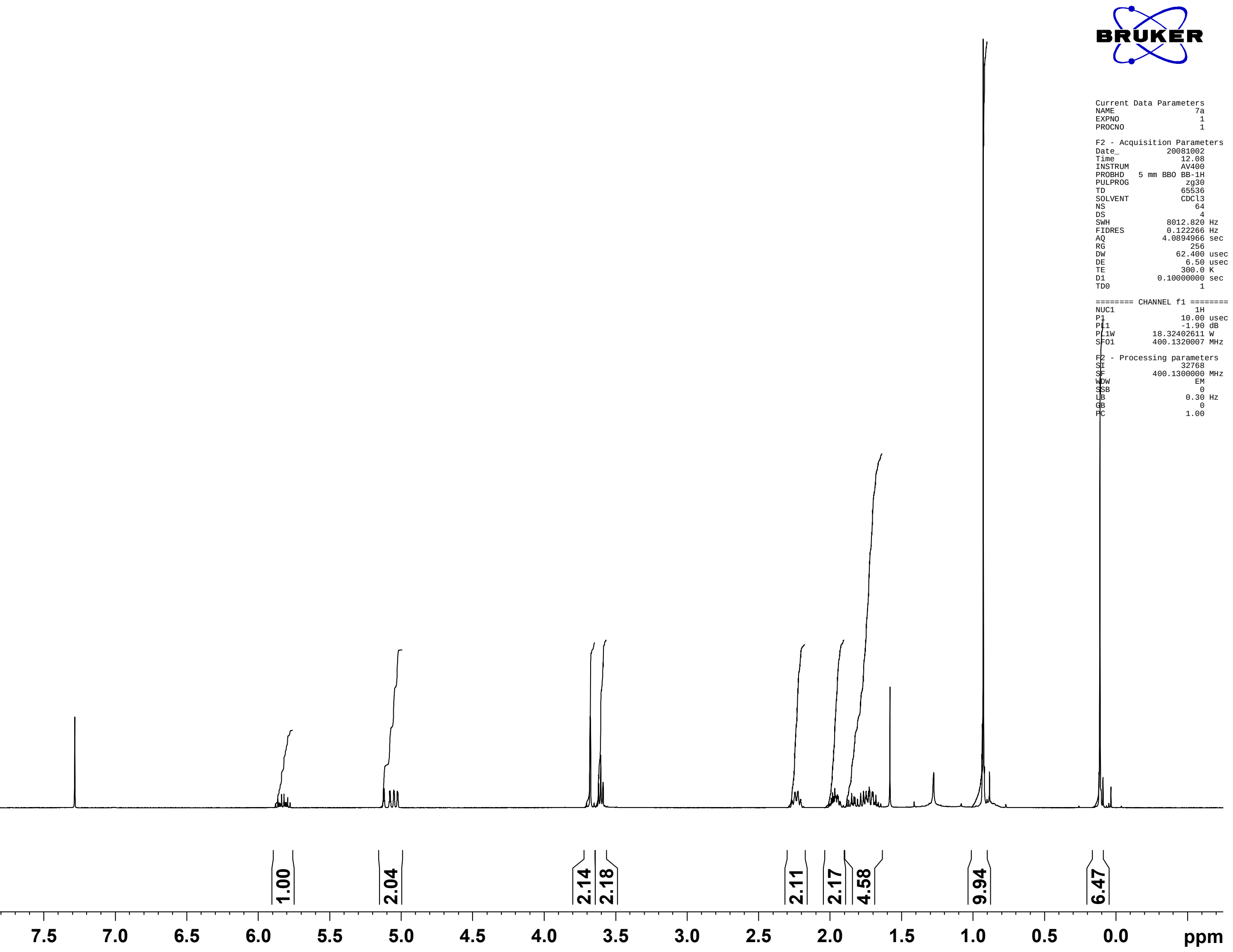

BRUKER
Current Data Parameters
NAME 7a
EXPNO 1
PROCNO 1
F2 - Acquisition Parameters
Date_ 20081002
Time 12.08
INSTRUM AV400
PROBHD 5 mm BBO BB-1H
PULPROG zg30
TD 65536
SOLVENT CDCl3
NS 64
DS 4
SWH 8012.820 Hz
FIDRES 0.122266 Hz
AQ 4.0894966 sec
RG 256
DW 62.400 usec
DE 6.50 usec
TE 300.0 K
D1 0.10000000 sec
TD0 1
======== CHANNEL f1 ========
NUC1 1H
P1 10.00 usec
PL1 -1.90 dB
PL1W 18.32402611 W
SFO1 400.1320007 MHz
F2 - Processing parameters
SI 32768
SF 400.1300000 MHz
WDW EM
SSB 0
LB 0.30 Hz
GB 0
PC 1.00
1.00
2.04
2.14
2.18
2.11
2.17
4.58
9.94
6.47
8.5
8.0
7.5
7.0
6.5
6.0
5.5
5.0
4.5
4.0
3.5
3.0
2.5
2.0
1.5
1.0
0.5
0.0
ppm

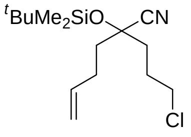

7a

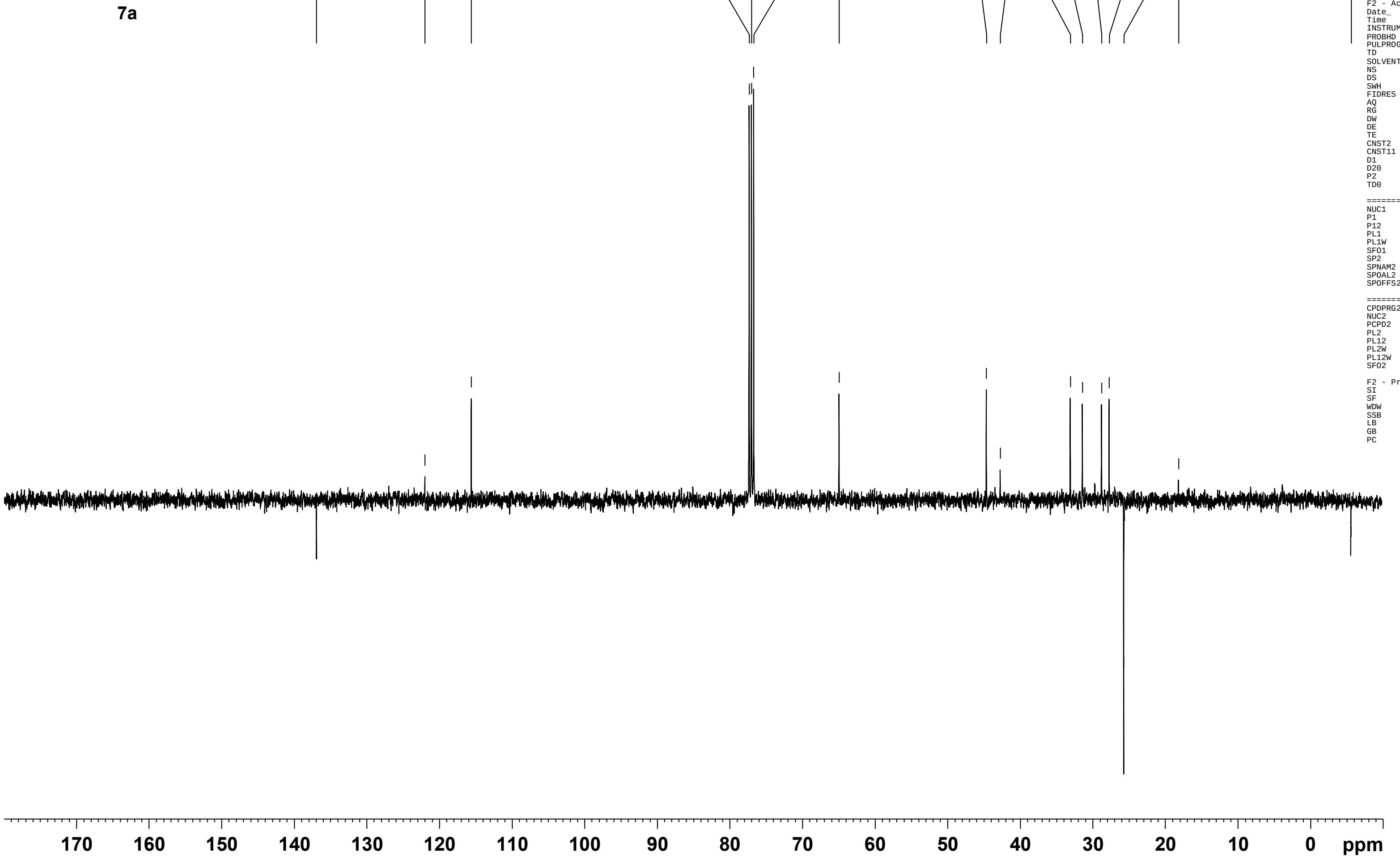


```
Current Data Parameters
NAME                 7a
EXPNO                11
PROCNO               1

F2 - Acquisition Parameters
Date_          20081002
Time              21.05
INSTRUM           AV400
PROBHD   5 mm BBO BB-1H
PULPROG          jmodsp
TD                65536
SOLVENT           CDCl3
NS                  512
DS                    4
SWH           25125.629 Hz
FIDRES         0.383387 Hz
AQ            1.3042164 sec
RG                16384
DW               19.900 usec
DE                 6.50 usec
TE                300.0 K
CNST2       145.0000000
CNST11        1.0000000
D1           4.00000000 sec
D20          0.00689655 sec
P2                16.00 usec
TD0                   1

======== CHANNEL f1 ========
NUC1                13C
P1                 8.00 usec
P12             2000.00 usec
PL1               -3.10 dB
PL1W        58.97905731 W
SFO1        100.6243395 MHz
SP2                7.00 dB
SPNAM2      Crp60comp.4
SPOAL2            0.500
SPOFFS2            0.00 Hz

======== CHANNEL f2 ========
CPDPRG2         waltz16
NUC2                 1H
PCPD2             88.00 usec
PL2               -1.90 dB
PL12              16.99 dB
PL2W        18.32402611 W
PL12W        0.23660338 W
SFO2        400.1316005 MHz

F2 - Processing parameters
SI                32768
SF          100.6127690 MHz
WDW                  EM
SSB                   0
LB                 2.00 Hz
GB                    0
PC                 1.40
```

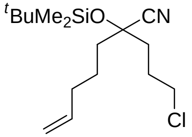

^{t}BuMe$_2$SiO
CN
Cl

7b

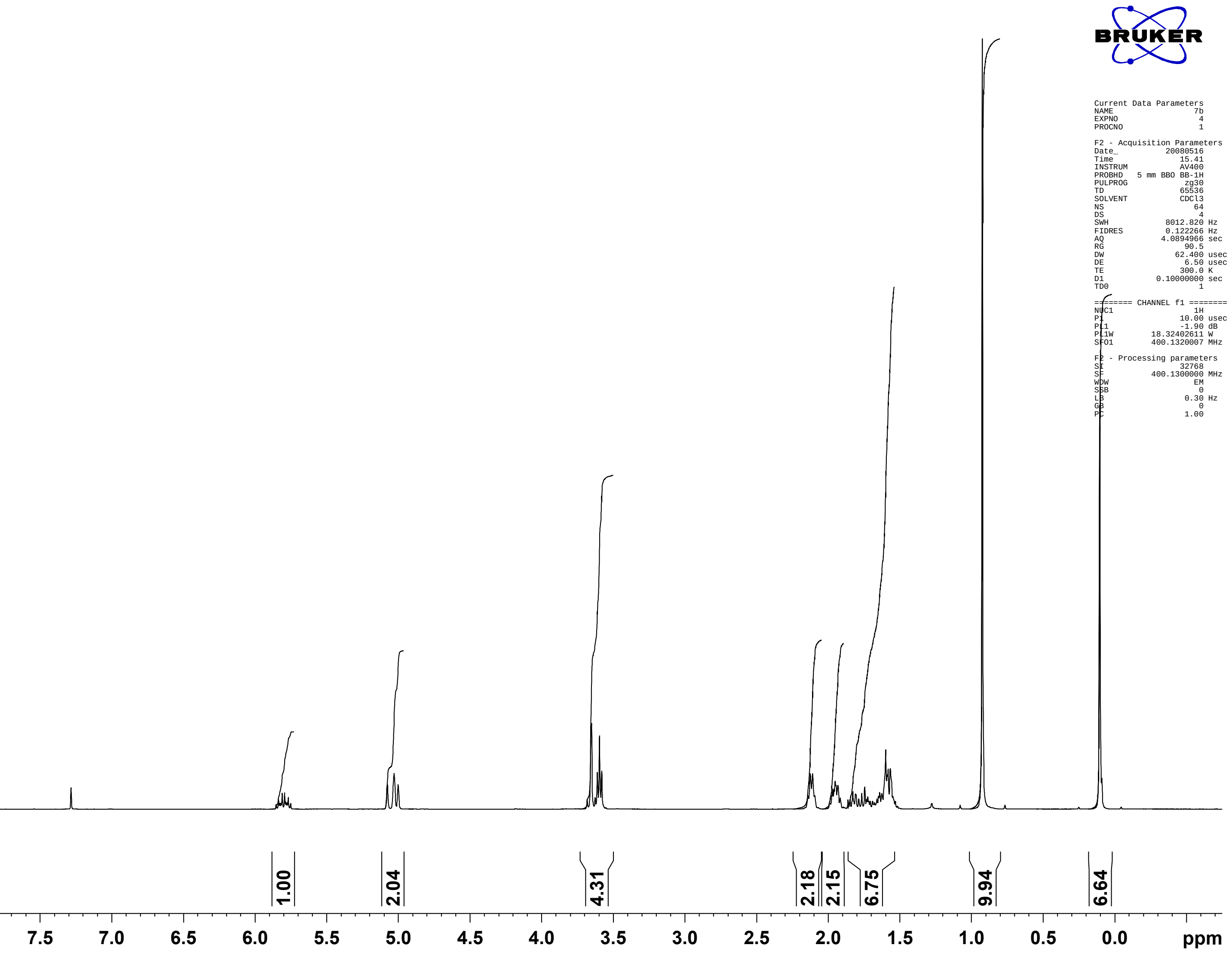

Current Data Parameters
NAME 7b
EXPNO 4
PROCNO 1
F2 - Acquisition Parameters
Date_ 20080516
Time 15.41
INSTRUM AV400
PROBHD 5 mm BBO BB-1H
PULPROG zg30
TD 65536
SOLVENT CDCl3
NS 64
DS 4
SWH 8012.820 Hz
FIDRES 0.122266 Hz
AQ 4.0894966 sec
RG 90.5
DW 62.400 usec
DE 6.50 usec
TE 300.0 K
D1 0.10000000 sec
TD0 1
======== CHANNEL f1 ========
NUC1 1H
P1 10.00 usec
PL1 -1.90 dB
PL1W 18.32402611 W
SFO1 400.1320007 MHz
F2 - Processing parameters
SI 32768
SF 400.1300000 MHz
WDW EM
SSB 0
LB 0.30 Hz
GB 0
PC 1.00
1.00
2.04
4.31
2.18
2.15
6.75
9.94
6.64
8.5
8.0
7.5
7.0
6.5
6.0
5.5
5.0
4.5
4.0
3.5
3.0
2.5
2.0
1.5
1.0
0.5
0.0
ppm

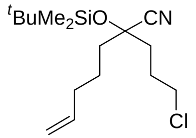

7b

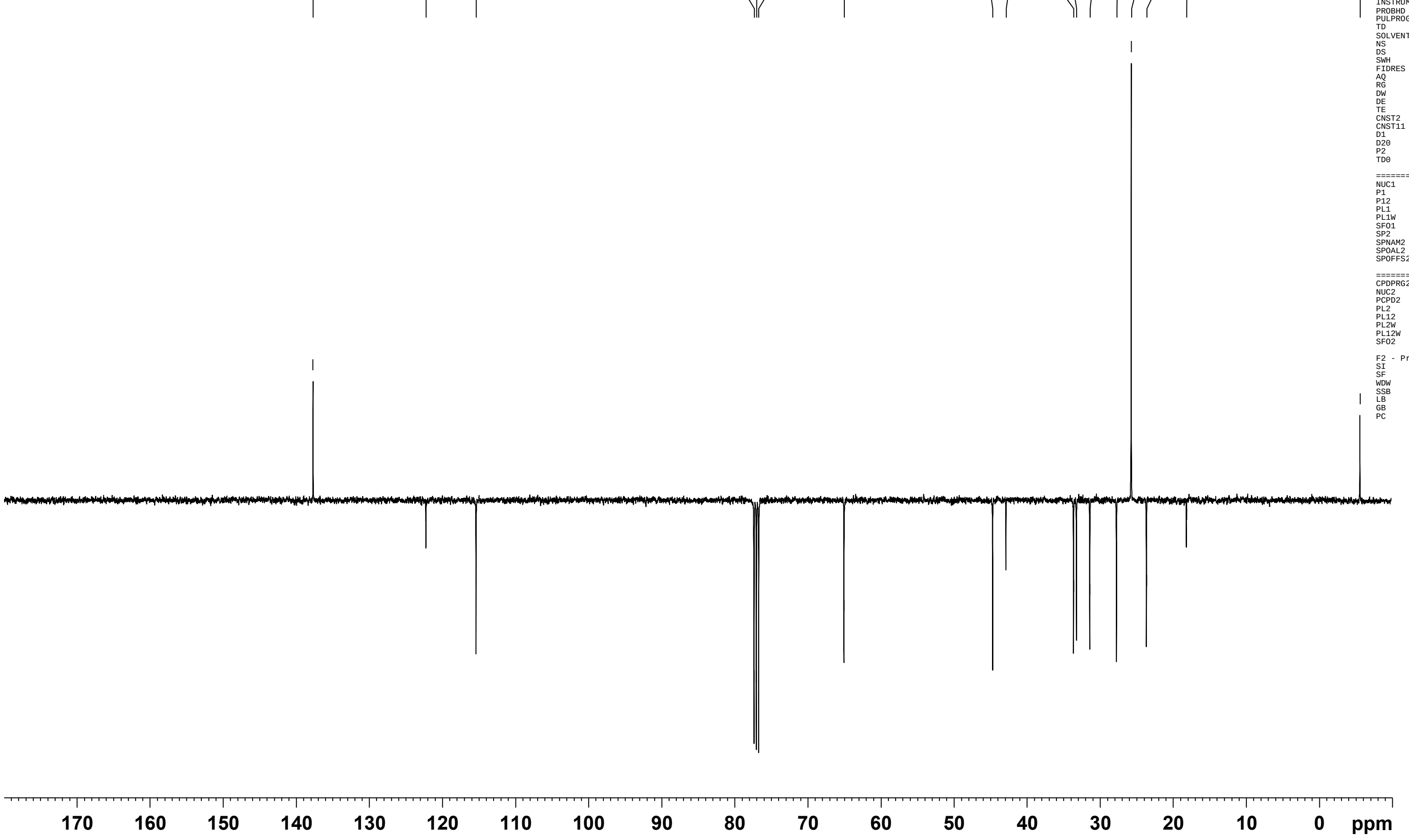


```
Current Data Parameters
NAME                 7b
EXPNO                12
PROCNO                1

F2 - Acquisition Parameters
Date_          20080517
Time               1.10
INSTRUM           AV400
PROBHD   5 mm BBO BB-1H
PULPROG          jmodsp
TD                65536
SOLVENT           CDCl3
NS                  512
DS                    4
SWH           25125.629 Hz
FIDRES         0.383387 Hz
AQ            1.3042164 sec
RG                16384
DW               19.900 usec
DE                 6.50 usec
TE                300.0 K
CNST2       145.0000000
CNST11        1.0000000
D1           4.00000000 sec
D20          0.00689655 sec
P2                16.00 usec
TD0                   1

======== CHANNEL f1 ========
NUC1                13C
P1                 8.00 usec
P12             2000.00 usec
PL1               -3.10 dB
PL1W        58.97905731 W
SFO1        100.6243395 MHz
SP2                7.00 dB
SPNAM2      Crp60comp.4
SPOAL2            0.500
SPOFFS2            0.00 Hz

======== CHANNEL f2 ========
CPDPRG2         waltz16
NUC2                 1H
PCPD2             88.00 usec
PL2               -1.90 dB
PL12              16.99 dB
PL2W        18.32402611 W
PL12W        0.23660338 W
SFO2        400.1316005 MHz

F2 - Processing parameters
SI                32768
SF          100.6127690 MHz
WDW                  EM
SSB                   0
LB                 2.00 Hz
GB                    0
PC                 1.40
```

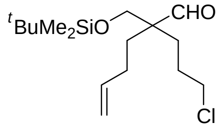

^{t}BuMe$_2$SiO
CHO
Cl

8a

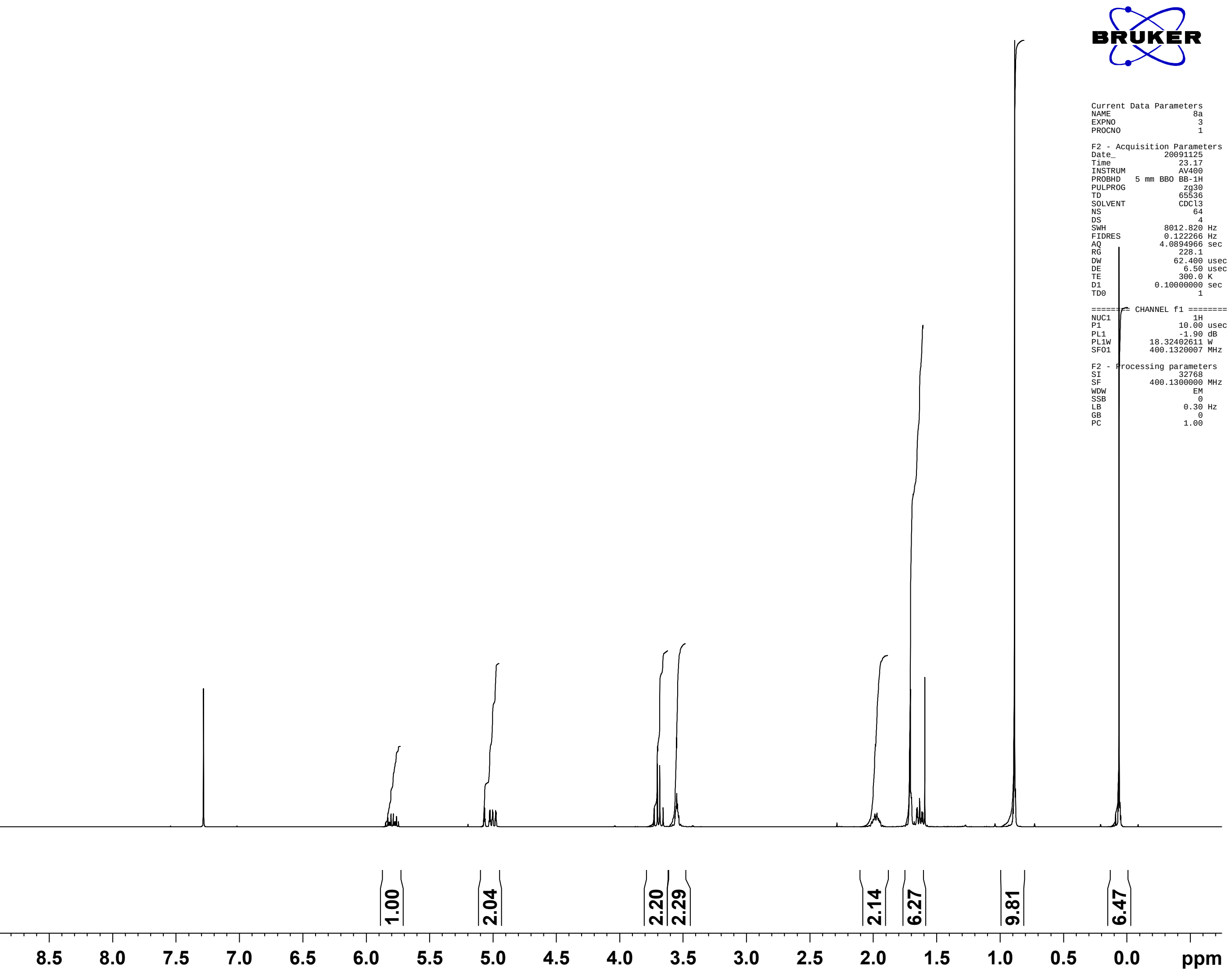

BRUKER
Current Data Parameters
NAME 8a
EXPNO 3
PROCNO 1
F2 - Acquisition Parameters
Date_ 20091125
Time 23.17
INSTRUM AV400
PROBHD 5 mm BBO BB-1H
PULPROG zg30
TD 65536
SOLVENT CDCl3
NS 64
DS 4
SWH 8012.820 Hz
FIDRES 0.122266 Hz
AQ 4.0894966 sec
RG 228.1
DW 62.400 usec
DE 6.50 usec
TE 300.0 K
D1 0.10000000 sec
TD0 1
======== CHANNEL f1 ========
NUC1 1H
P1 10.00 usec
PL1 -1.90 dB
PL1W 18.32402611 W
SFO1 400.1320007 MHz
F2 - Processing parameters
SI 32768
SF 400.1300000 MHz
WDW EM
SSB 0
LB 0.30 Hz
GB 0
PC 1.00
1.00
1.00
2.04
2.20
2.29
2.14
6.27
9.81
6.47
10.0 9.5 9.0 8.5 8.0 7.5 7.0 6.5 6.0 5.5 5.0 4.5 4.0 3.5 3.0 2.5 2.0 1.5 1.0 0.5 0.0 ppm

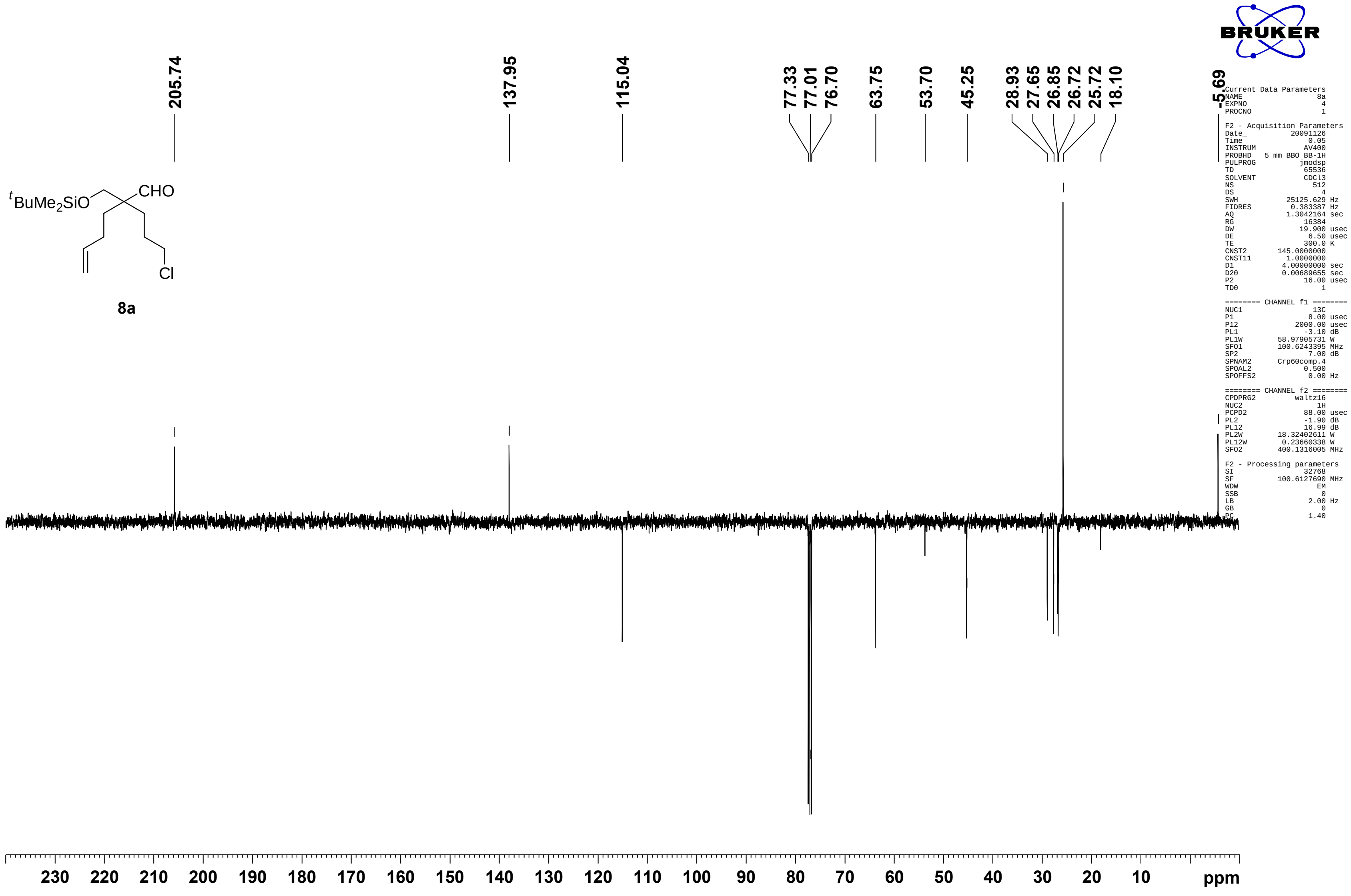
205.74
137.95
115.04
77.33
77.01
76.70
63.75
53.70
45.25
28.93
27.65
26.85
26.72
25.72
18.10
-5.69
tBuMe2SiO
CHO
Cl
8a
Current Data Parameters
NAME 8a
EXPNO 4
PROCNO 1
F2 - Acquisition Parameters
Date_ 20091126
Time 0.05
INSTRUM AV400
PROBHD 5 mm BBO BB-1H
PULPROG jmodsp
TD 65536
SOLVENT CDCl3
NS 512
DS 4
SWH 25125.629 Hz
FIDRES 0.383387 Hz
AQ 1.3042164 sec
RG 16384
DW 19.900 usec
DE 6.50 usec
TE 300.0 K
CNST2 145.0000000
CNST11 1.0000000
D1 4.00000000 sec
D20 0.00689655 sec
P2 16.00 usec
TD0 1
======== CHANNEL f1 ========
NUC1 13C
P1 8.00 usec
P12 2000.00 usec
PL1 -3.10 dB
PL1W 58.97905731 W
SFO1 100.6243395 MHz
SP2 7.00 dB
SPNAM2 Crp60comp.4
SPOAL2 0.500
SPOFFS2 0.00 Hz
======== CHANNEL f2 ========
CPDPRG2 waltz16
NUC2 1H
PCPD2 88.00 usec
PL2 -1.90 dB
PL12 16.99 dB
PL2W 18.32402611 W
PL12W 0.23660338 W
SFO2 400.1316005 MHz
F2 - Processing parameters
SI 32768
SF 100.6127690 MHz
WDW EM
SSB 0
LB 2.00 Hz
GB 0
PC 1.40
230 220 210 200 190 180 170 160 150 140 130 120 110 100 90 80 70 60 50 40 30 20 10 ppm

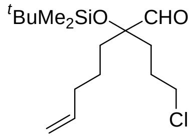
tBuMe2SiO
CHO
Cl

8b

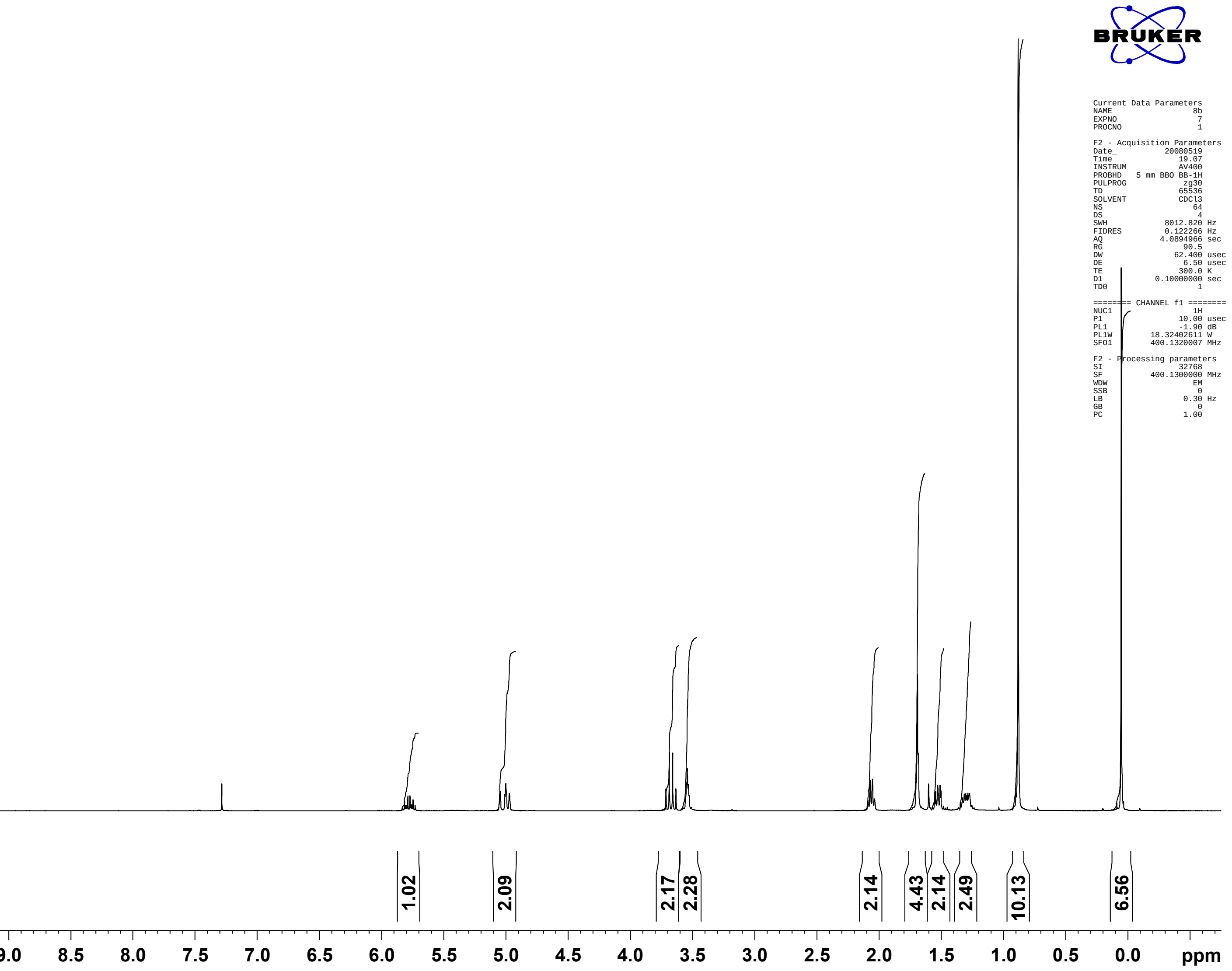
Current Data Parameters
NAME 8b
EXPNO 7
PROCNO 1
F2 - Acquisition Parameters
Date_ 20080519
Time 19.07
INSTRUM AV400
PROBHD 5 mm BBO BB-1H
PULPROG zg30
TD 65536
SOLVENT CDCl3
NS 64
DS 4
SWH 8012.820 Hz
FIDRES 0.122266 Hz
AQ 4.0894966 sec
RG 90.5
DW 62.400 usec
DE 6.50 usec
TE 300.0 K
D1 0.10000000 sec
TD0 1
======== CHANNEL f1 ========
NUC1 1H
P1 10.00 usec
PL1 -1.90 dB
PL1W 18.32402611 W
SFO1 400.1320007 MHz
F2 - Processing parameters
SI 32768
SF 400.1300000 MHz
WDW EM
SSB 0
LB 0.30 Hz
GB 0
PC 1.00
1.00
1.02
2.09
2.17
2.28
2.14
4.43
2.14
2.49
10.13
6.56
10.0
9.5
9.0
8.5
8.0
7.5
7.0
6.5
6.0
5.5
5.0
4.5
4.0
3.5
3.0
2.5
2.0
1.5
1.0
0.5
0.0
ppm

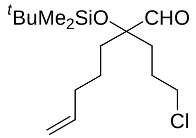
$^tBuMe_2SiO$
CHO
Cl

8b

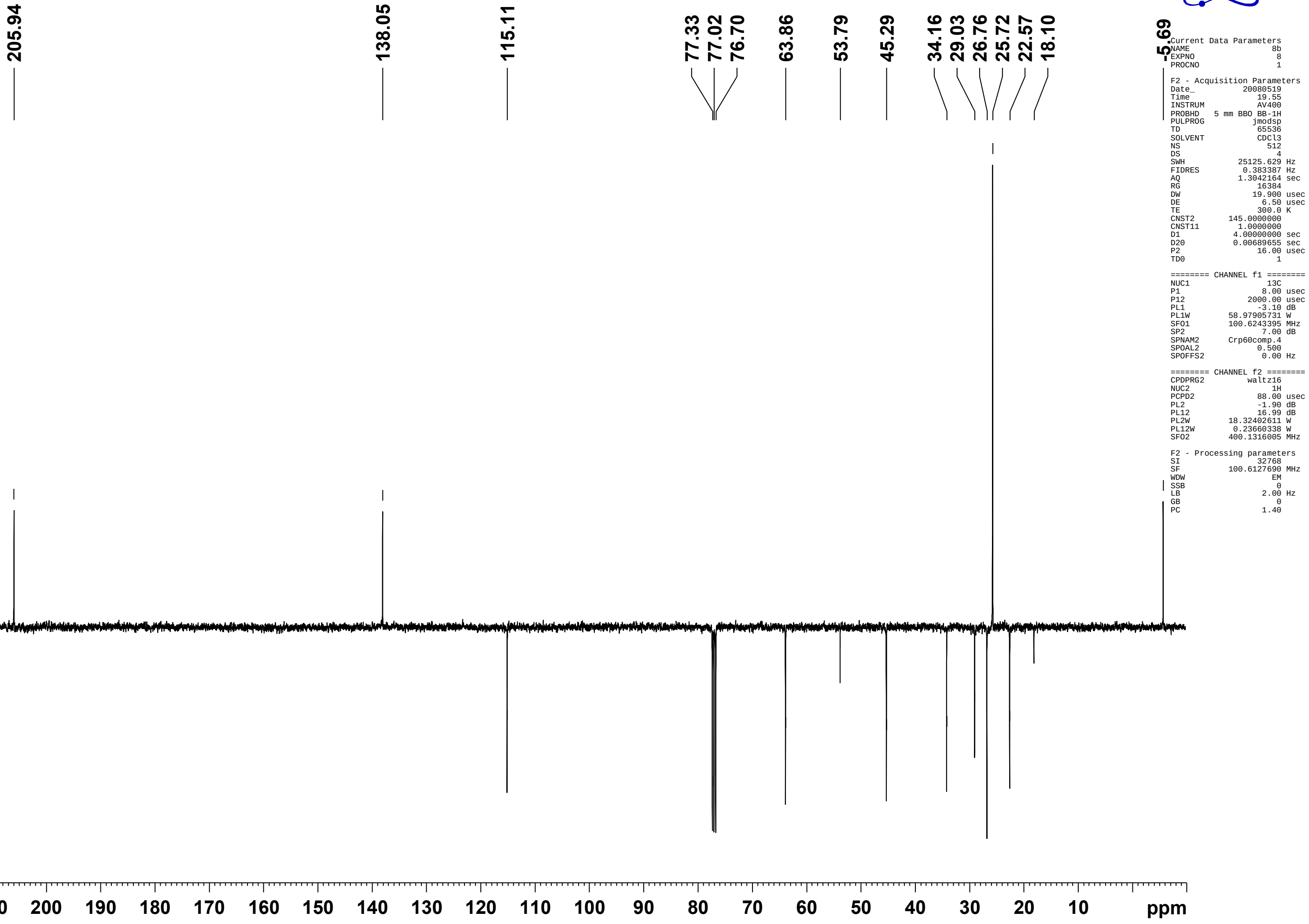
205.94
138.05
115.11
77.33
77.02
76.70
63.86
53.79
45.29
34.16
29.03
26.76
25.72
22.57
18.10
-5.69
Current Data Parameters
NAME 8b
EXPNO 8
PROCNO 1
F2 - Acquisition Parameters
Date_ 20080519
Time 19.55
INSTRUM AV400
PROBHD 5 mm BBO BB-1H
PULPROG jmodsp
TD 65536
SOLVENT CDCl3
NS 512
DS 4
SWH 25125.629 Hz
FIDRES 0.383387 Hz
AQ 1.3042164 sec
RG 16384
DW 19.900 usec
DE 6.50 usec
TE 300.0 K
CNST2 145.0000000
CNST11 1.0000000
D1 4.00000000 sec
D20 0.00689655 sec
P2 16.00 usec
TD0 1
======== CHANNEL f1 ========
NUC1 13C
P1 8.00 usec
P12 2000.00 usec
PL1 -3.10 dB
PL1W 58.97905731 W
SFO1 100.6243395 MHz
SP2 7.00 dB
SPNAM2 Crp60comp.4
SPOAL2 0.500
SPOFFS2 0.00 Hz
======== CHANNEL f2 ========
CPDPRG2 waltz16
NUC2 1H
PCPD2 88.00 usec
PL2 -1.90 dB
PL12 16.99 dB
PL2W 18.32402611 W
PL12W 0.23660338 W
SFO2 400.1316005 MHz
F2 - Processing parameters
SI 32768
SF 100.6127690 MHz
WDW EM
SSB 0
LB 2.00 Hz
GB 0
PC 1.40
230 220 210 200 190 180 170 160 150 140 130 120 110 100 90 80 70 60 50 40 30 20 10 ppm

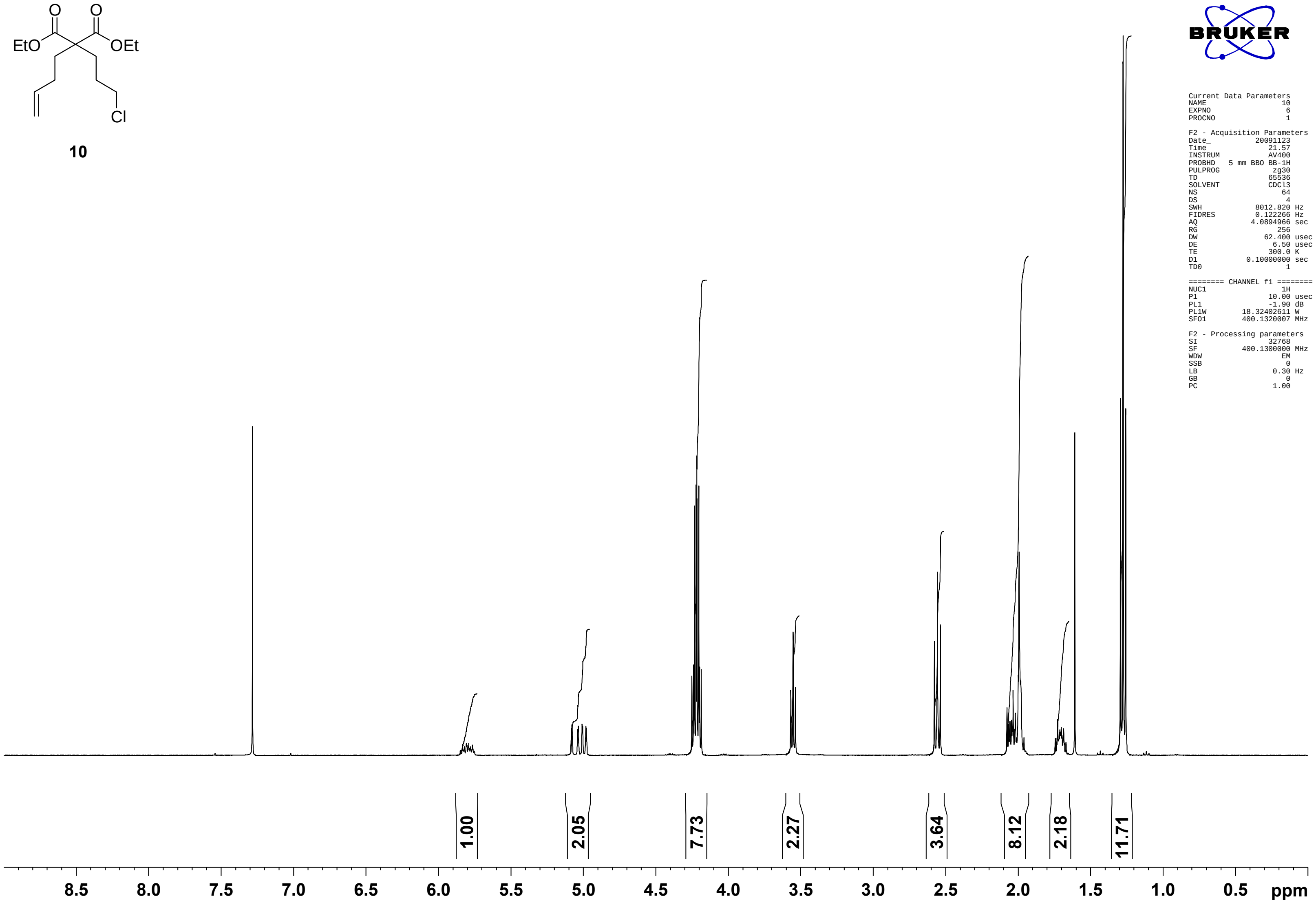


```
Current Data Parameters
NAME                10
EXPNO                6
PROCNO               1

F2 - Acquisition Parameters
Date_         20091123
Time             21.57
INSTRUM          AV400
PROBHD   5 mm BBO BB-1H
PULPROG           zg30
TD               65536
SOLVENT          CDCl3
NS                  64
DS                   4
SWH           8012.820 Hz
FIDRES        0.122266 Hz
AQ           4.0894966 sec
RG                 256
DW              62.400 usec
DE                6.50 usec
TE               300.0 K
D1          0.10000000 sec
TD0                  1

======== CHANNEL f1 ========
NUC1                1H
P1               10.00 usec
PL1              -1.90 dB
PL1W       18.32402611 W
SFO1       400.1320007 MHz

F2 - Processing parameters
SI               32768
SF         400.1300000 MHz
WDW                 EM
SSB                  0
LB                0.30 Hz
GB                   0
PC                1.00
```

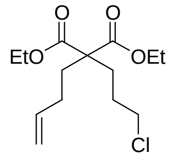
O
O
EtO
OEt
Cl

10

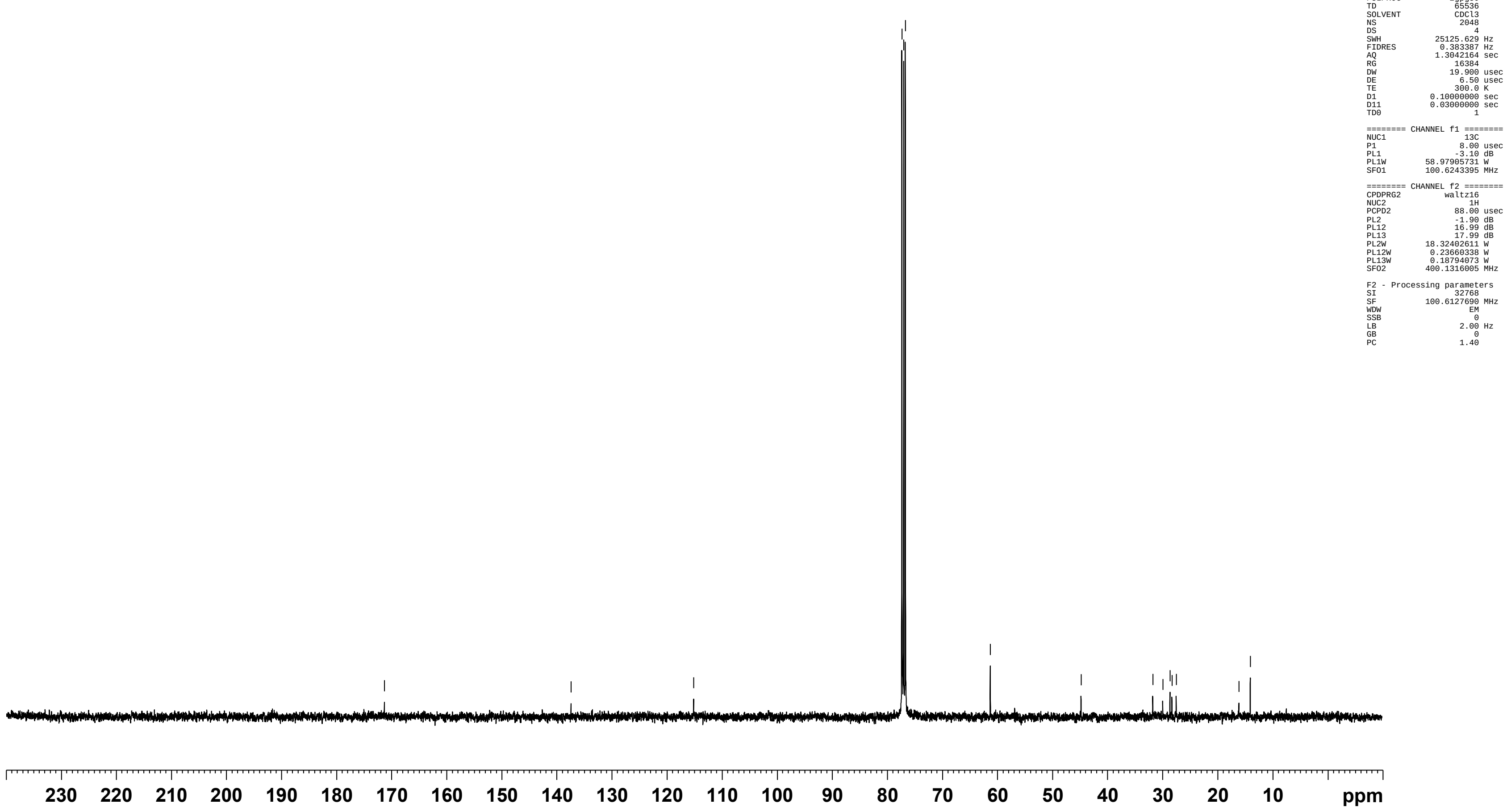
171.30
137.42
115.15
77.33
77.01
76.69
61.28
44.81
31.77
29.97
28.64
28.31
27.52
16.14
14.09
230
220
210
200
190
180
170
160
150
140
130
120
110
100
90
80
70
60
50
40
30
20
10
ppm
BRUKER
Current Data Parameters
NAME 10
EXPNO 5
PROCNO 1
F2 - Acquisition Parameters
Date_ 20100813
Time 0.32
INSTRUM AV400
PROBHD 5 mm BBO BB-1H
PULPROG zgpg30
TD 65536
SOLVENT CDCl3
NS 2048
DS 4
SWH 25125.629 Hz
FIDRES 0.383387 Hz
AQ 1.3042164 sec
RG 16384
DW 19.900 usec
DE 6.50 usec
TE 300.0 K
D1 0.10000000 sec
D11 0.03000000 sec
TD0 1
======== CHANNEL f1 ========
NUC1 13C
P1 8.00 usec
PL1 -3.10 dB
PL1W 58.97905731 W
SFO1 100.6243395 MHz
======== CHANNEL f2 ========
CPDPRG2 waltz16
NUC2 1H
PCPD2 88.00 usec
PL2 -1.90 dB
PL12 16.99 dB
PL13 17.99 dB
PL2W 18.32402611 W
PL12W 0.23660338 W
PL13W 0.18794073 W
SFO2 400.1316005 MHz
F2 - Processing parameters
SI 32768
SF 100.6127690 MHz
WDW EM
SSB 0
LB 2.00 Hz
GB 0
PC 1.40

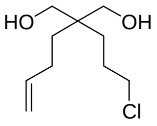

HO
OH
Cl

11

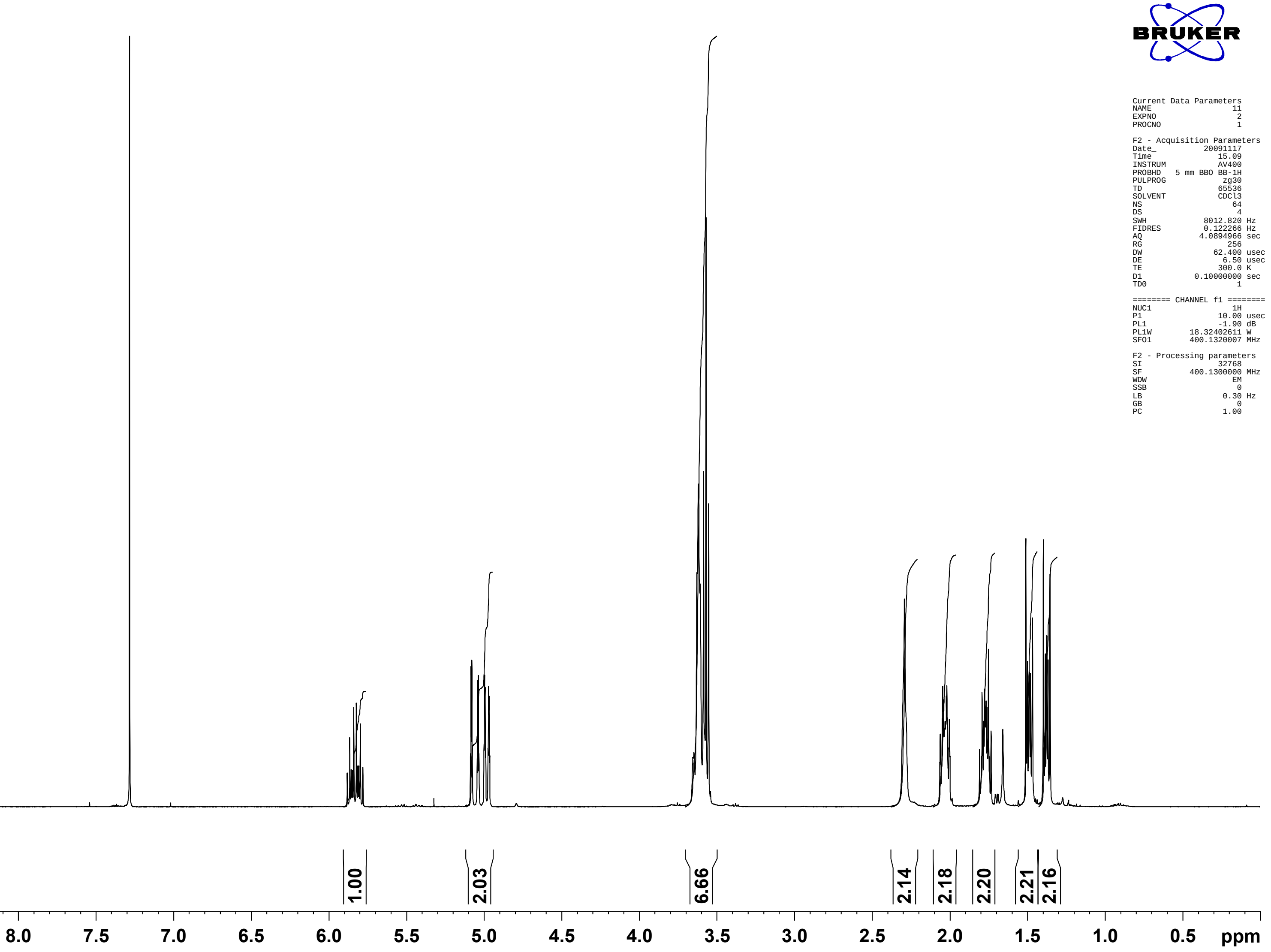

BRUKER
Current Data Parameters
NAME 11
EXPNO 2
PROCNO 1
F2 - Acquisition Parameters
Date_ 20091117
Time 15.09
INSTRUM AV400
PROBHD 5 mm BBO BB-1H
PULPROG zg30
TD 65536
SOLVENT CDCl3
NS 64
DS 4
SWH 8012.820 Hz
FIDRES 0.122266 Hz
AQ 4.0894966 sec
RG 256
DW 62.400 usec
DE 6.50 usec
TE 300.0 K
D1 0.10000000 sec
TD0 1
======== CHANNEL f1 ========
NUC1 1H
P1 10.00 usec
PL1 -1.90 dB
PL1W 18.32402611 W
SFO1 400.1320007 MHz
F2 - Processing parameters
SI 32768
SF 400.1300000 MHz
WDW EM
SSB 0
LB 0.30 Hz
GB 0
PC 1.00
1.00
2.03
6.66
2.14
2.18
2.20
2.21
2.16
8.5
8.0
7.5
7.0
6.5
6.0
5.5
5.0
4.5
4.0
3.5
3.0
2.5
2.0
1.5
1.0
0.5
ppm

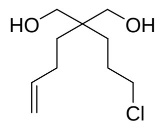

11

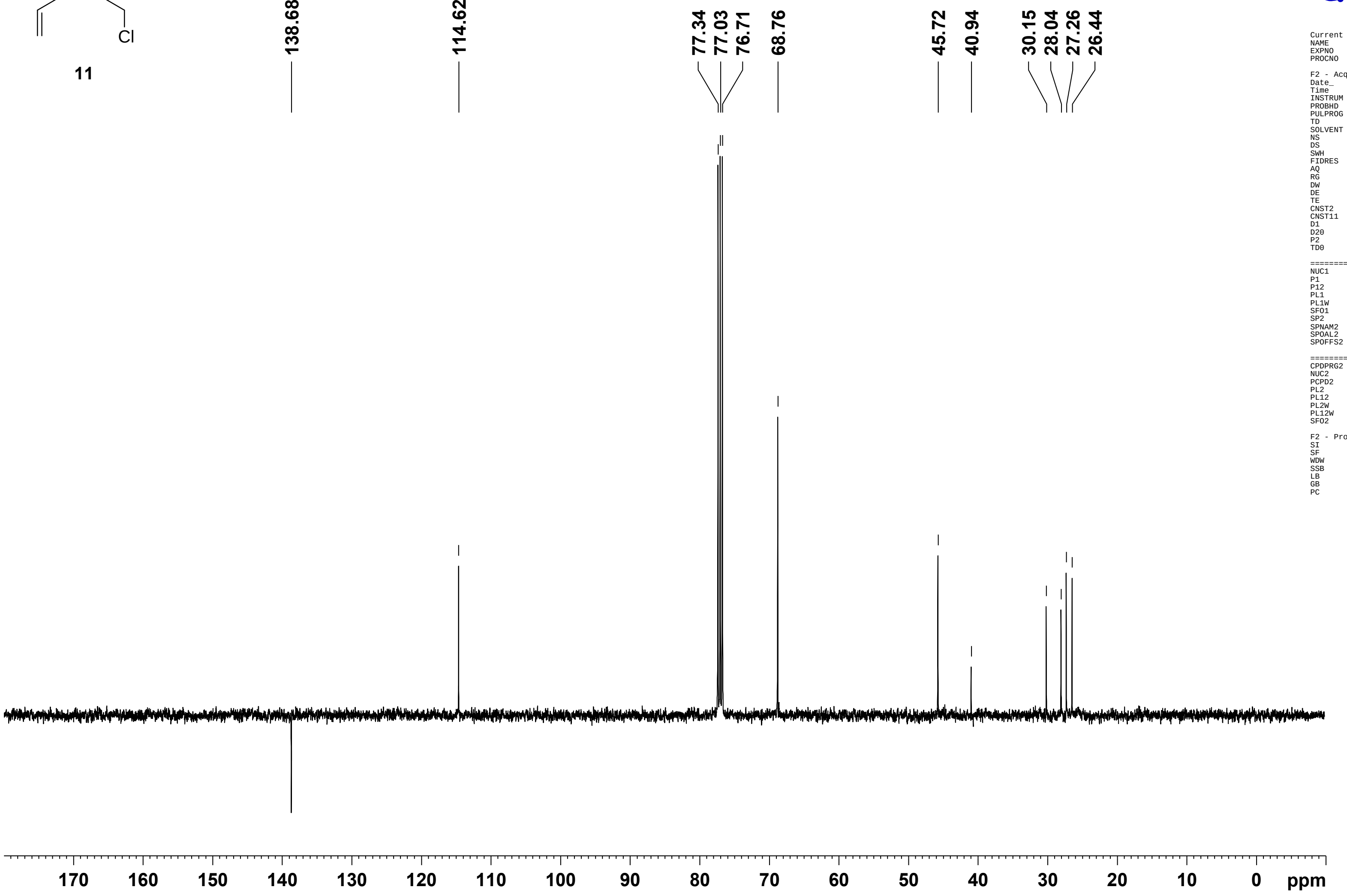


```
Current Data Parameters
NAME                 11
EXPNO                 7
PROCNO                1

F2 - Acquisition Parameters
Date_          20091117
Time              23.52
INSTRUM           AV400
PROBHD   5 mm BBO BB-1H
PULPROG          jmodsp
TD                65536
SOLVENT           CDCl3
NS                  512
DS                    4
SWH           25125.629 Hz
FIDRES         0.383387 Hz
AQ            1.3042164 sec
RG                16384
DW               19.900 usec
DE                 6.50 usec
TE                300.0 K
CNST2       145.0000000
CNST11        1.0000000
D1           4.00000000 sec
D20          0.00689655 sec
P2                16.00 usec
TD0                   1

======== CHANNEL f1 ========
NUC1                13C
P1                 8.00 usec
P12             2000.00 usec
PL1               -3.10 dB
PL1W        58.97905731 W
SFO1        100.6243395 MHz
SP2                7.00 dB
SPNAM2      Crp60comp.4
SPOAL2            0.500
SPOFFS2            0.00 Hz

======== CHANNEL f2 ========
CPDPRG2         waltz16
NUC2                 1H
PCPD2             88.00 usec
PL2               -1.90 dB
PL12              16.99 dB
PL2W        18.32402611 W
PL12W        0.23660338 W
SFO2        400.1316005 MHz

F2 - Processing parameters
SI                32768
SF          100.6127690 MHz
WDW                  EM
SSB                   0
LB                 2.00 Hz
GB                    0
PC                 1.40
```

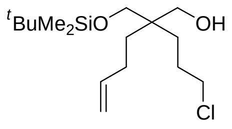
^{t}BuMe$_2$SiO
OH
Cl

12

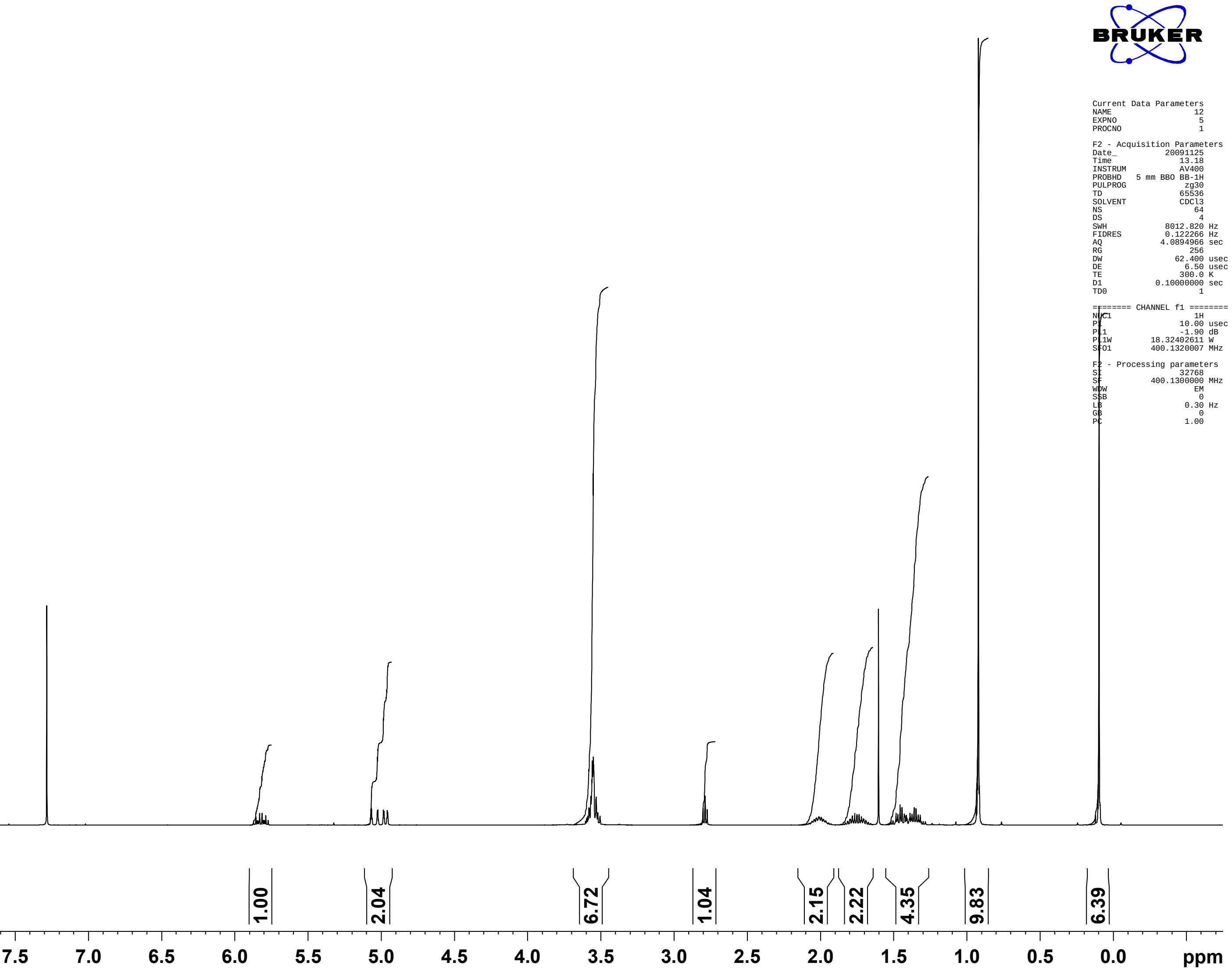
BRUKER
Current Data Parameters
NAME 12
EXPNO 5
PROCNO 1
F2 - Acquisition Parameters
Date_ 20091125
Time 13.18
INSTRUM AV400
PROBHD 5 mm BBO BB-1H
PULPROG zg30
TD 65536
SOLVENT CDCl3
NS 64
DS 4
SWH 8012.820 Hz
FIDRES 0.122266 Hz
AQ 4.0894966 sec
RG 256
DW 62.400 usec
DE 6.50 usec
TE 300.0 K
D1 0.10000000 sec
TD0 1
======== CHANNEL f1 ========
NUC1 1H
P1 10.00 usec
PL1 -1.90 dB
PL1W 18.32402611 W
SFO1 400.1320007 MHz
F2 - Processing parameters
SI 32768
SF 400.1300000 MHz
WDW EM
SSB 0
LB 0.30 Hz
GB 0
PC 1.00
1.00
2.04
6.72
1.04
2.15
2.22
4.35
9.83
6.39
8.5
8.0
7.5
7.0
6.5
6.0
5.5
5.0
4.5
4.0
3.5
3.0
2.5
2.0
1.5
1.0
0.5
0.0
ppm

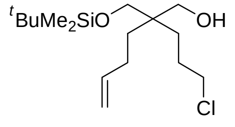
tBuMe2SiO
OH
Cl

12

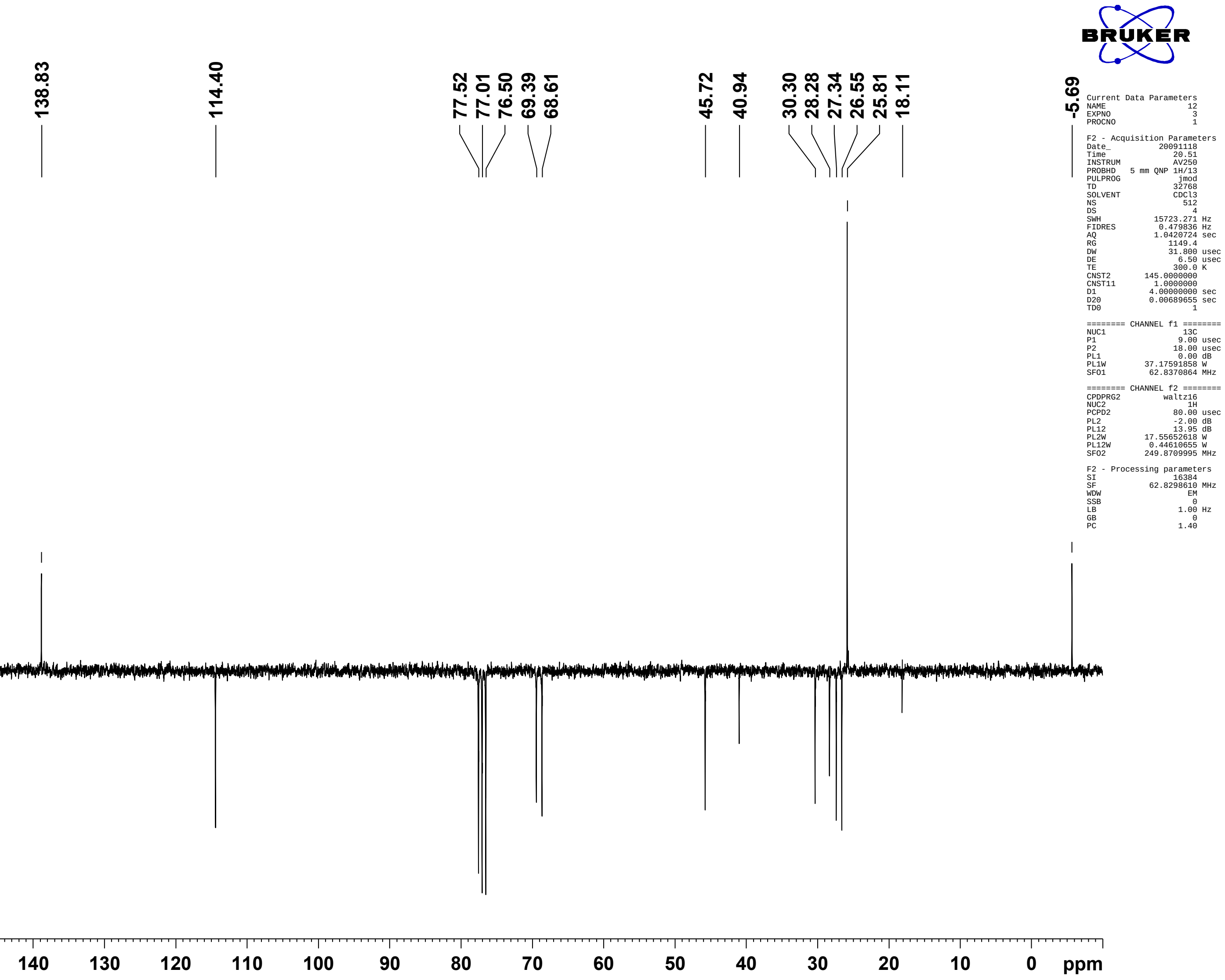
138.83
114.40
77.52
77.01
76.50
69.39
68.61
45.72
40.94
30.30
28.28
27.34
26.55
25.81
18.11
-5.69
BRUKER
Current Data Parameters
NAME 12
EXPNO 3
PROCNO 1
F2 - Acquisition Parameters
Date_ 20091118
Time 20.51
INSTRUM AV250
PROBHD 5 mm QNP 1H/13
PULPROG jmod
TD 32768
SOLVENT CDCl3
NS 512
DS 4
SWH 15723.271 Hz
FIDRES 0.479836 Hz
AQ 1.0420724 sec
RG 1149.4
DW 31.800 usec
DE 6.50 usec
TE 300.0 K
CNST2 145.0000000
CNST11 1.0000000
D1 4.00000000 sec
D20 0.00689655 sec
TD0 1
======== CHANNEL f1 ========
NUC1 13C
P1 9.00 usec
P2 18.00 usec
PL1 0.00 dB
PL1W 37.17591858 W
SFO1 62.8370864 MHz
======== CHANNEL f2 ========
CPDPRG2 waltz16
NUC2 1H
PCPD2 80.00 usec
PL2 -2.00 dB
PL12 13.95 dB
PL2W 17.55652618 W
PL12W 0.44610655 W
SFO2 249.8709995 MHz
F2 - Processing parameters
SI 16384
SF 62.8298610 MHz
WDW EM
SSB 0
LB 1.00 Hz
GB 0
PC 1.40
170
160
150
140
130
120
110
100
90
80
70
60
50
40
30
20
10
0
ppm

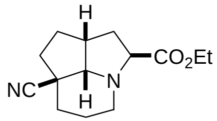
H
CO2Et
N
NC
H

13a

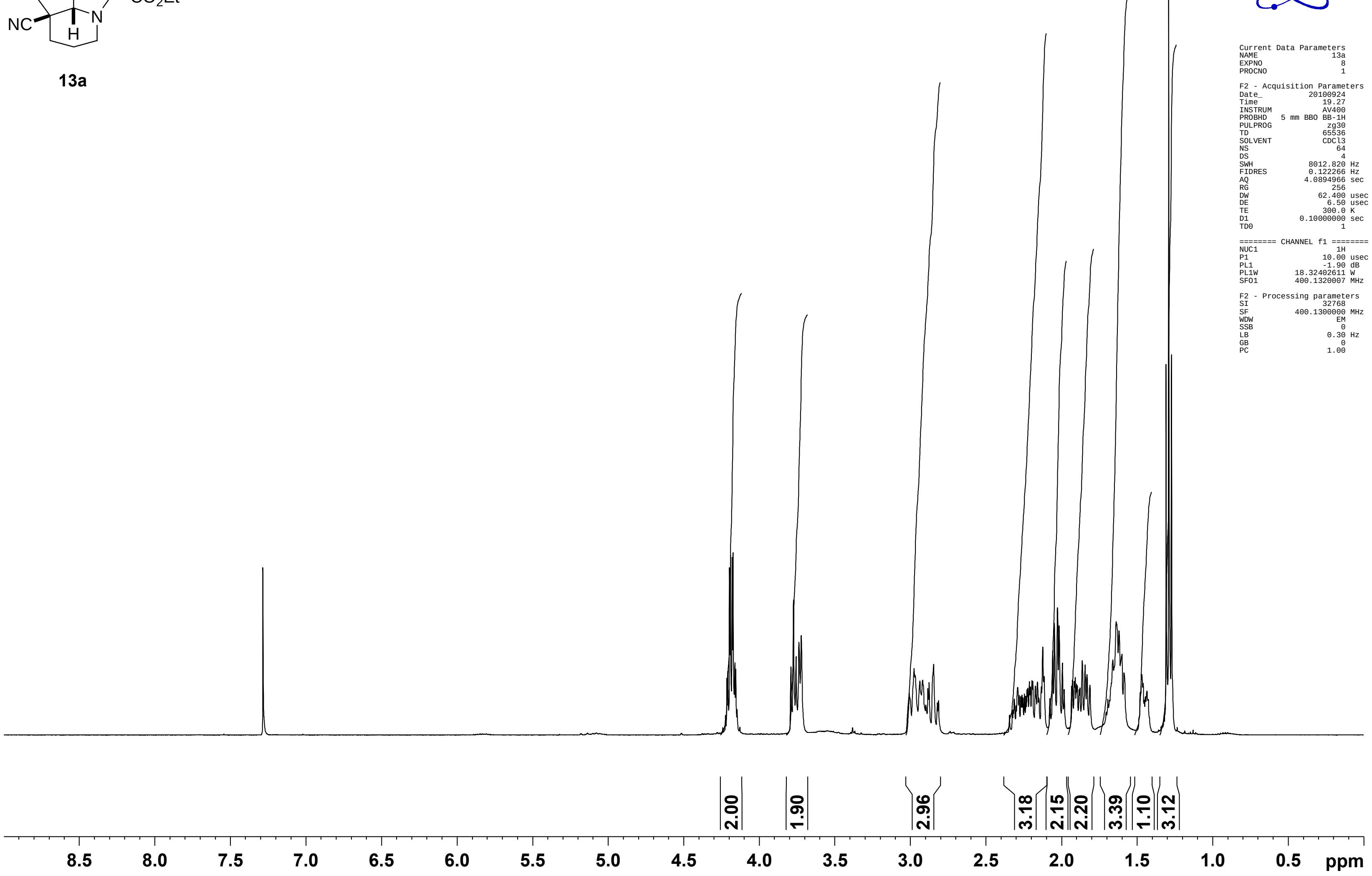
Current Data Parameters
NAME 13a
EXPNO 8
PROCNO 1
F2 - Acquisition Parameters
Date_ 20100924
Time 19.27
INSTRUM AV400
PROBHD 5 mm BBO BB-1H
PULPROG zg30
TD 65536
SOLVENT CDCl3
NS 64
DS 4
SWH 8012.820 Hz
FIDRES 0.122266 Hz
AQ 4.0894966 sec
RG 256
DW 62.400 usec
DE 6.50 usec
TE 300.0 K
D1 0.10000000 sec
TD0 1
======== CHANNEL f1 ========
NUC1 1H
P1 10.00 usec
PL1 -1.90 dB
PL1W 18.32402611 W
SFO1 400.1320007 MHz
F2 - Processing parameters
SI 32768
SF 400.1300000 MHz
WDW EM
SSB 0
LB 0.30 Hz
GB 0
PC 1.00
2.00
1.90
2.96
3.18
2.15
2.20
3.39
1.10
3.12
8.5
8.0
7.5
7.0
6.5
6.0
5.5
5.0
4.5
4.0
3.5
3.0
2.5
2.0
1.5
1.0
0.5
ppm

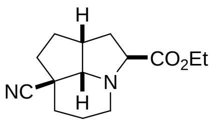

13a

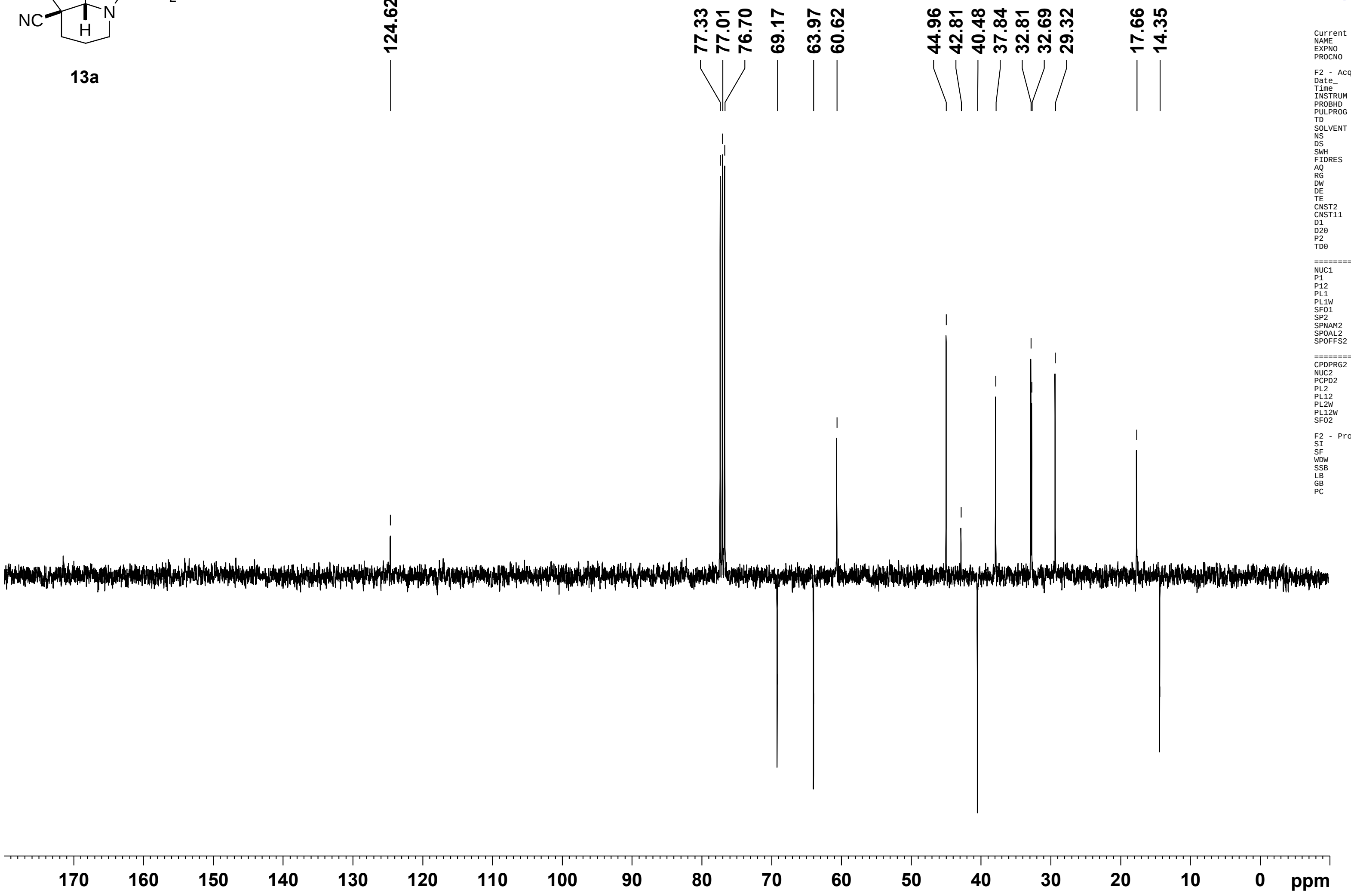


```
Current Data Parameters
NAME                 13a
EXPNO                  9
PROCNO                 1

F2 - Acquisition Parameters
Date_           20100924
Time               20.14
INSTRUM            AV400
PROBHD   5 mm BBO BB-1H
PULPROG           jmodsp
TD                 65536
SOLVENT            CDCl3
NS                   512
DS                     4
SWH            25125.629 Hz
FIDRES          0.383387 Hz
AQ             1.3042164 sec
RG                 16384
DW                19.900 usec
DE                  6.50 usec
TE                 300.0 K
CNST2        145.0000000
CNST11         1.0000000
D1            4.00000000 sec
D20           0.00689655 sec
P2                 16.00 usec
TD0                    1

======== CHANNEL f1 ========
NUC1                 13C
P1                  8.00 usec
P12              2000.00 usec
PL1                -3.10 dB
PL1W         58.97905731 W
SFO1         100.6243395 MHz
SP2                 7.00 dB
SPNAM2      Crp60comp.4
SPOAL2             0.500
SPOFFS2             0.00 Hz

======== CHANNEL f2 ========
CPDPRG2          waltz16
NUC2                  1H
PCPD2              88.00 usec
PL2                -1.90 dB
PL12               16.99 dB
PL2W         18.32402611 W
PL12W         0.23660338 W
SFO2         400.1316005 MHz

F2 - Processing parameters
SI                 32768
SF           100.6127690 MHz
WDW                   EM
SSB                    0
LB                  2.00 Hz
GB                     0
PC                  1.40
```

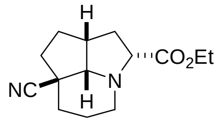

13b

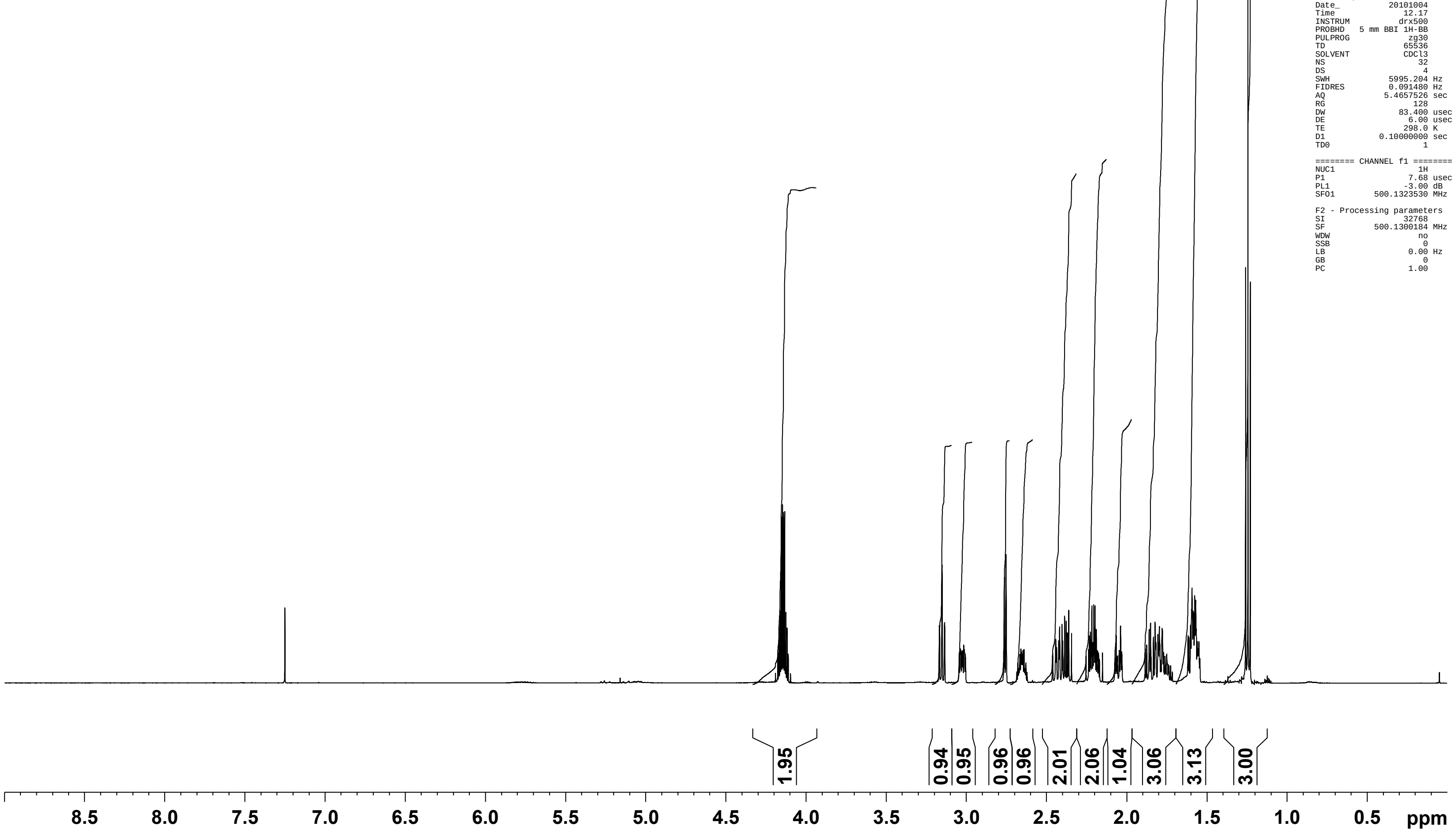


```
Current Data Parameters
NAME              13b'
EXPNO              500
PROCNO               1

F2 - Acquisition Parameters
Date_         20101004
Time             12.17
INSTRUM         drx500
PROBHD   5 mm BBI 1H-BB
PULPROG           zg30
TD               65536
SOLVENT          CDCl3
NS                  32
DS                   4
SWH           5995.204 Hz
FIDRES        0.091480 Hz
AQ           5.4657526 sec
RG                 128
DW              83.400 usec
DE                6.00 usec
TE               298.0 K
D1          0.10000000 sec
TD0                  1

======== CHANNEL f1 ========
NUC1                1H
P1                7.68 usec
PL1              -3.00 dB
SFO1       500.1323530 MHz

F2 - Processing parameters
SI               32768
SF         500.1300184 MHz
WDW                 no
SSB                  0
LB                0.00 Hz
GB                   0
PC                1.00
```

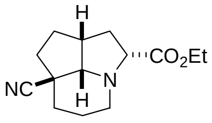

13b

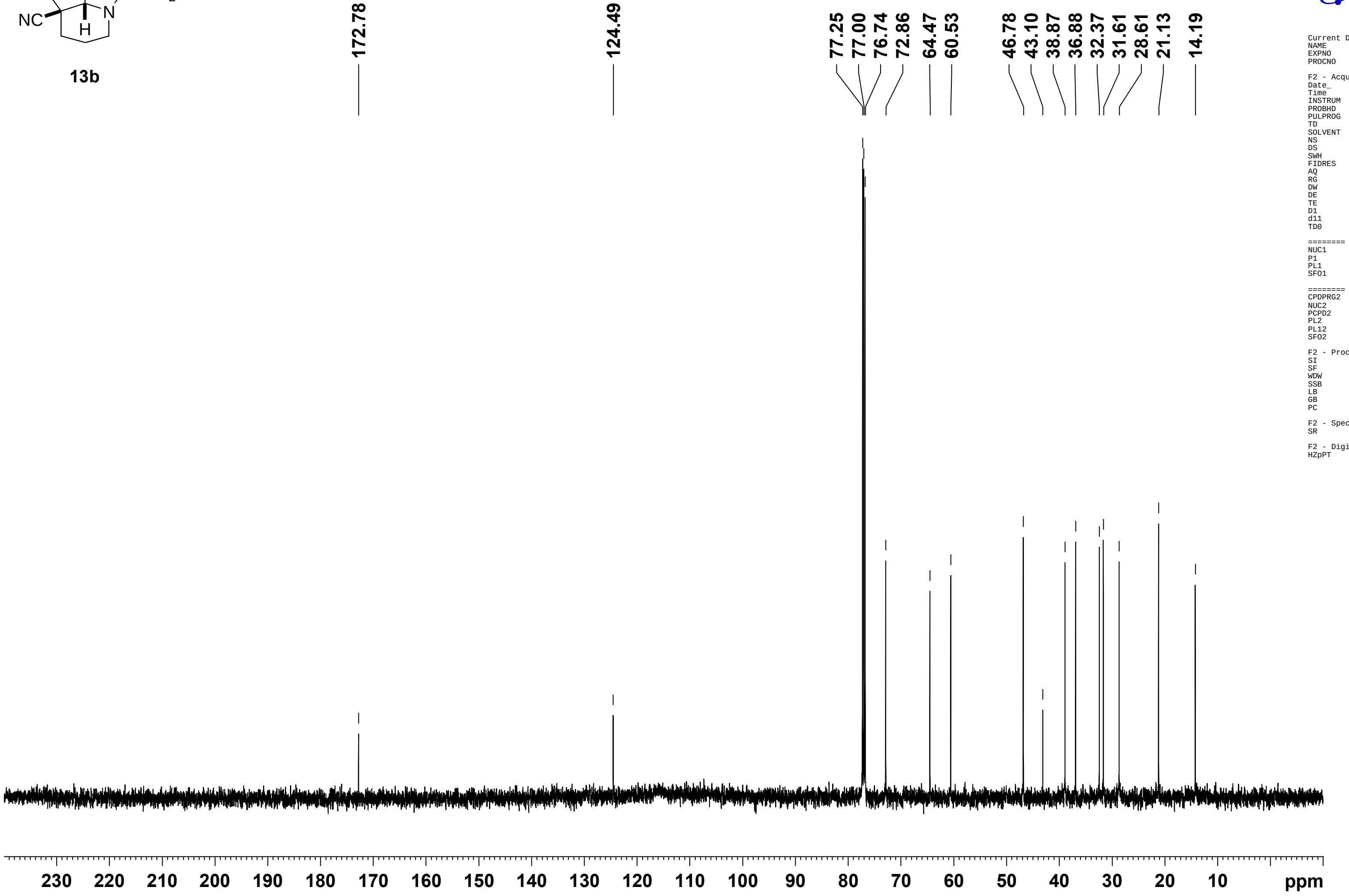


```
Current Data Parameters
NAME              Oct04
EXPNO               503
PROCNO                1

F2 - Acquisition Parameters
Date_          20101004
Time              13.38
INSTRUM          drx500
PROBHD   5 mm BBI 1H-BB
PULPROG          zgdc30
TD                65536
SOLVENT           CDCl3
NS                  265
DS                    4
SWH           31446.541 Hz
FIDRES         0.479836 Hz
AQ            1.0420724 sec
RG                16384
DW               15.900 usec
DE                 6.00 usec
TE                298.0 K
D1           0.10000000 sec
d11          0.03000000 sec
TD0                   1

======== CHANNEL f1 ========
NUC1                13C
P1                14.75 usec
PL1               -3.00 dB
SFO1        125.7722511 MHz

======== CHANNEL f2 ========
CPDPRG2         waltz16
NUC2                 1H
PCPD2             80.00 usec
PL2               -3.00 dB
PL12              18.00 dB
SFO2        500.1325007 MHz

F2 - Processing parameters
SI                32768
SF          125.7577944 MHz
WDW                  EM
SSB                   0
LB                 2.00 Hz
GB                    0
PC                 1.40

F2 - Spectrum reference
SR                 5.35 Hz

F2 - Digital resolution: Hz per point
HZpPT          0.959672 Hz
```

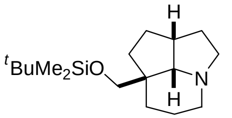
^{t}BuMe$_2$SiO
H
H
N

14

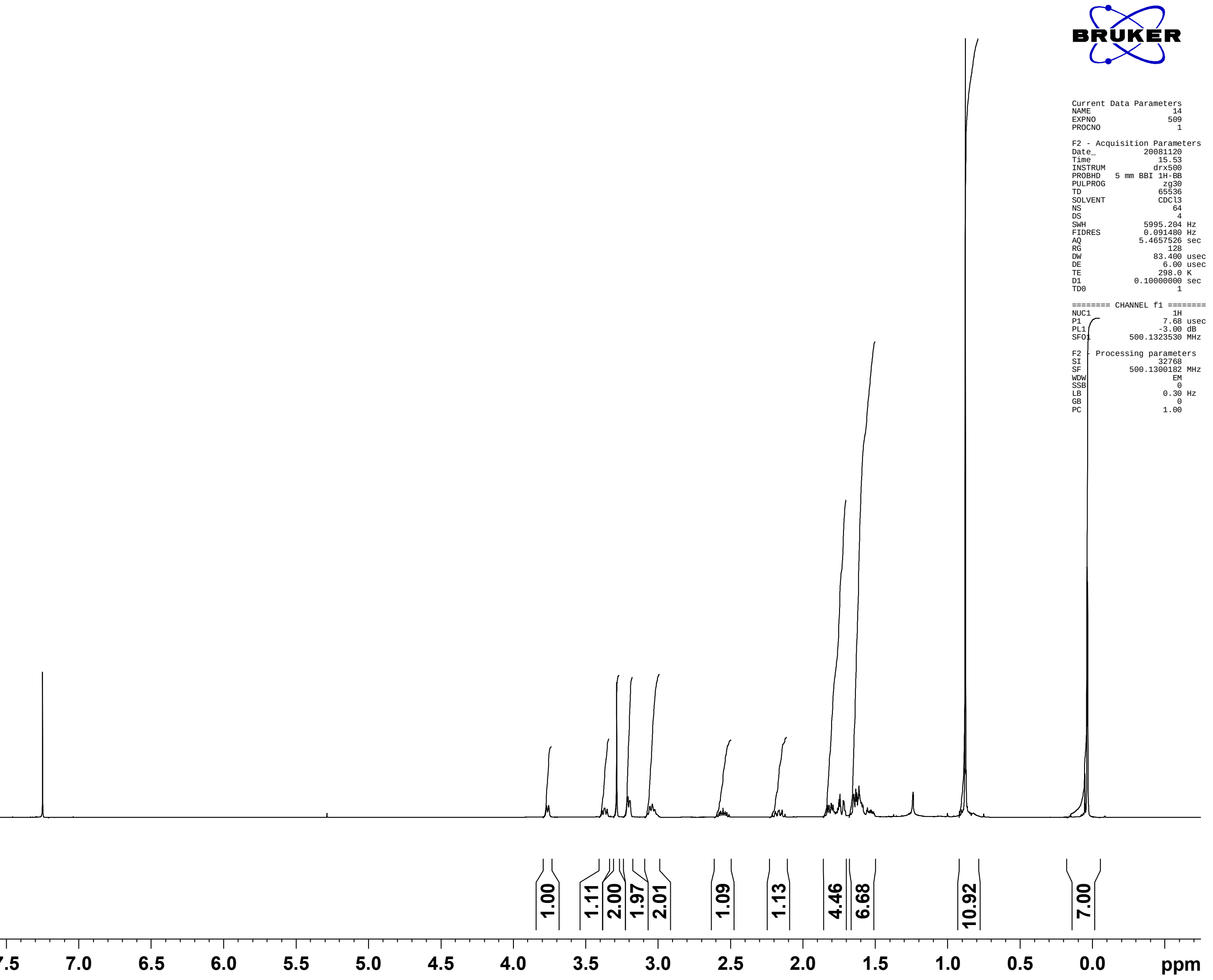
Current Data Parameters
NAME 14
EXPNO 509
PROCNO 1
F2 - Acquisition Parameters
Date_ 20081120
Time 15.53
INSTRUM drx500
PROBHD 5 mm BBI 1H-BB
PULPROG zg30
TD 65536
SOLVENT CDCl3
NS 64
DS 4
SWH 5995.204 Hz
FIDRES 0.091480 Hz
AQ 5.4657526 sec
RG 128
DW 83.400 usec
DE 6.00 usec
TE 298.0 K
D1 0.10000000 sec
TD0 1
======== CHANNEL f1 ========
NUC1 1H
P1 7.68 usec
PL1 -3.00 dB
SFO1 500.1323530 MHz
F2 - Processing parameters
SI 32768
SF 500.1300182 MHz
WDW EM
SSB 0
LB 0.30 Hz
GB 0
PC 1.00
1.00
1.11
2.00
1.97
2.01
1.09
1.13
4.46
6.68
10.92
7.00
8.5
8.0
7.5
7.0
6.5
6.0
5.5
5.0
4.5
4.0
3.5
3.0
2.5
2.0
1.5
1.0
0.5
0.0
ppm

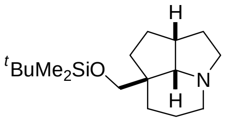

$^tBuMe_2SiO$
H
N
H

14

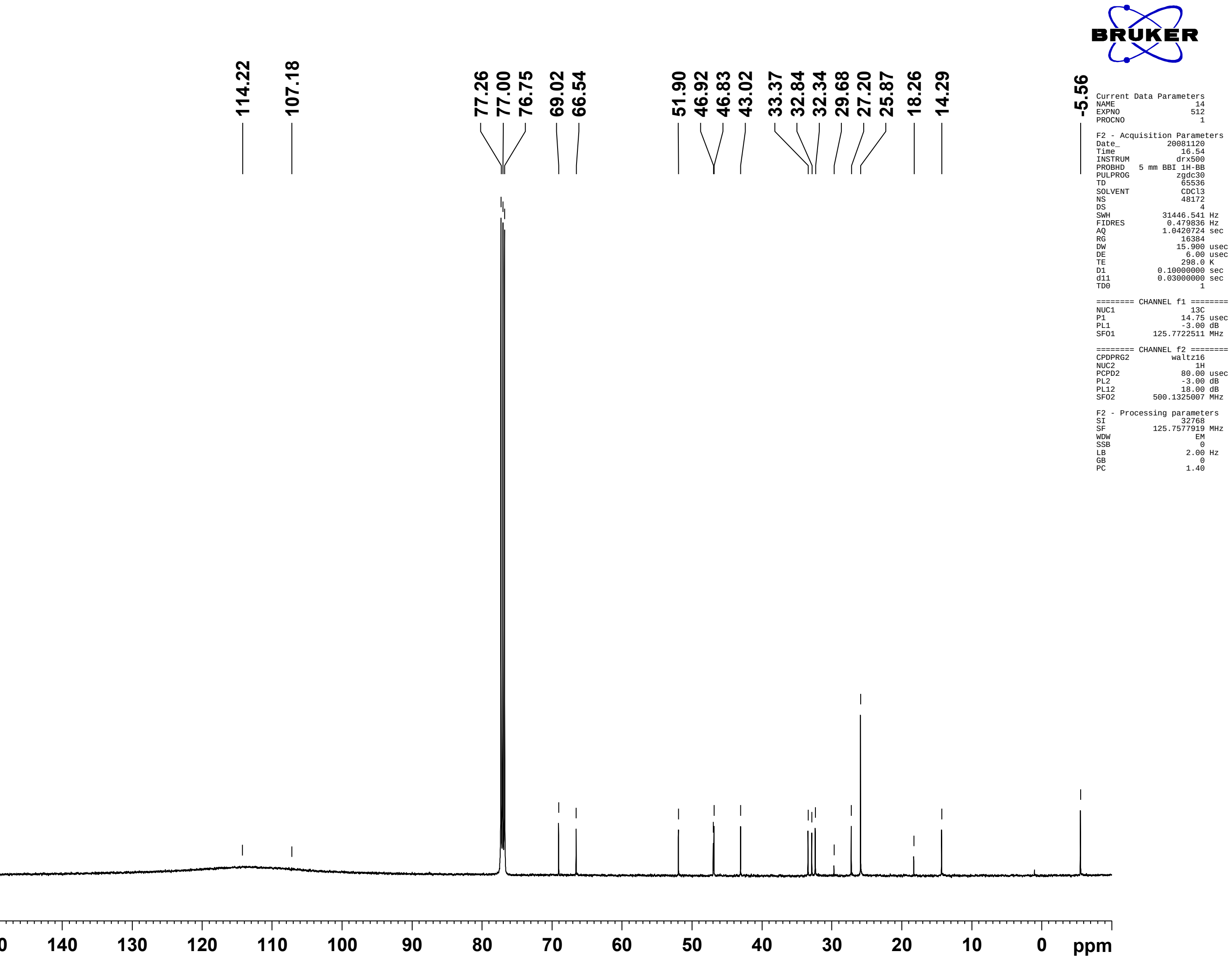

114.22
107.18
77.26
77.00
76.75
69.02
66.54
51.90
46.92
46.83
43.02
33.37
32.84
32.34
29.68
27.20
25.87
18.26
14.29
-5.56
BRUKER
Current Data Parameters
NAME 14
EXPNO 512
PROCNO 1
F2 - Acquisition Parameters
Date_ 20081120
Time 16.54
INSTRUM drx500
PROBHD 5 mm BBI 1H-BB
PULPROG zgdc30
TD 65536
SOLVENT CDCl3
NS 48172
DS 4
SWH 31446.541 Hz
FIDRES 0.479836 Hz
AQ 1.0420724 sec
RG 16384
DW 15.900 usec
DE 6.00 usec
TE 298.0 K
D1 0.10000000 sec
d11 0.03000000 sec
TD0 1
======== CHANNEL f1 ========
NUC1 13C
P1 14.75 usec
PL1 -3.00 dB
SFO1 125.7722511 MHz
======== CHANNEL f2 ========
CPDPRG2 waltz16
NUC2 1H
PCPD2 80.00 usec
PL2 -3.00 dB
PL12 18.00 dB
SFO2 500.1325007 MHz
F2 - Processing parameters
SI 32768
SF 125.7577919 MHz
WDW EM
SSB 0
LB 2.00 Hz
GB 0
PC 1.40
170 160 150 140 130 120 110 100 90 80 70 60 50 40 30 20 10 0 ppm

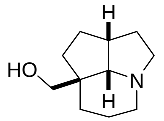
HO
H
H
N

15

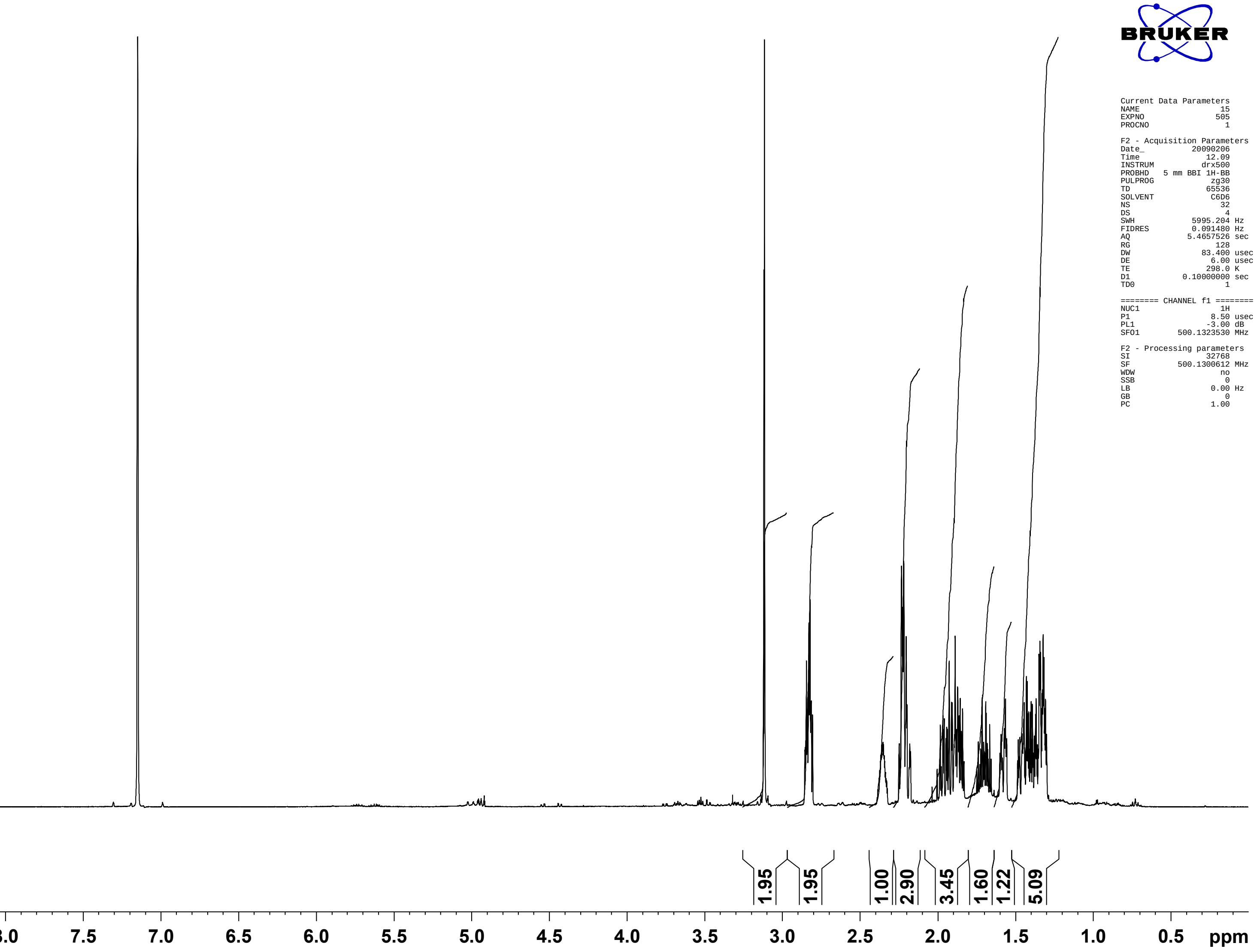
BRUKER
Current Data Parameters
NAME 15
EXPNO 505
PROCNO 1
F2 - Acquisition Parameters
Date_ 20090206
Time 12.09
INSTRUM drx500
PROBHD 5 mm BBI 1H-BB
PULPROG zg30
TD 65536
SOLVENT C6D6
NS 32
DS 4
SWH 5995.204 Hz
FIDRES 0.091480 Hz
AQ 5.4657526 sec
RG 128
DW 83.400 usec
DE 6.00 usec
TE 298.0 K
D1 0.10000000 sec
TD0 1
======== CHANNEL f1 ========
NUC1 1H
P1 8.50 usec
PL1 -3.00 dB
SFO1 500.1323530 MHz
F2 - Processing parameters
SI 32768
SF 500.1300612 MHz
WDW no
SSB 0
LB 0.00 Hz
GB 0
PC 1.00
1.95
1.95
1.00
2.90
3.45
1.60
1.22
5.09
8.5
8.0
7.5
7.0
6.5
6.0
5.5
5.0
4.5
4.0
3.5
3.0
2.5
2.0
1.5
1.0
0.5
ppm

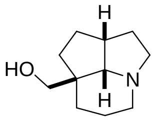
HO
H
N
H

15

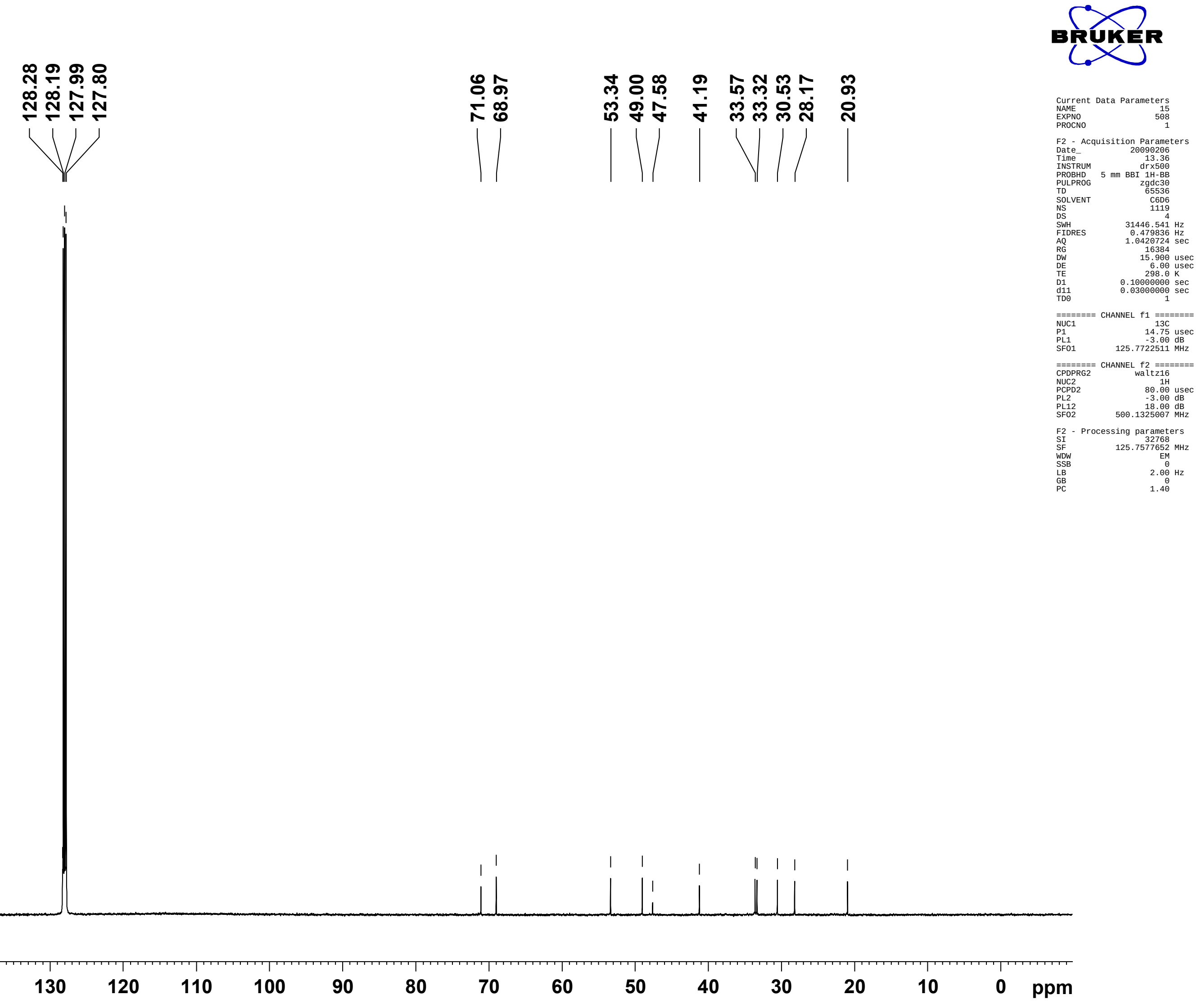
128.28
128.19
127.99
127.80
71.06
68.97
53.34
49.00
47.58
41.19
33.57
33.32
30.53
28.17
20.93
170
160
150
140
130
120
110
100
90
80
70
60
50
40
30
20
10
0
ppm
BRUKER
Current Data Parameters
NAME 15
EXPNO 508
PROCNO 1
F2 - Acquisition Parameters
Date_ 20090206
Time 13.36
INSTRUM drx500
PROBHD 5 mm BBI 1H-BB
PULPROG zgdc30
TD 65536
SOLVENT C6D6
NS 1119
DS 4
SWH 31446.541 Hz
FIDRES 0.479836 Hz
AQ 1.0420724 sec
RG 16384
DW 15.900 usec
DE 6.00 usec
TE 298.0 K
D1 0.10000000 sec
d11 0.03000000 sec
TD0 1
======== CHANNEL f1 ========
NUC1 13C
P1 14.75 usec
PL1 -3.00 dB
SFO1 125.7722511 MHz
======== CHANNEL f2 ========
CPDPRG2 waltz16
NUC2 1H
PCPD2 80.00 usec
PL2 -3.00 dB
PL12 18.00 dB
SFO2 500.1325007 MHz
F2 - Processing parameters
SI 32768
SF 125.7577652 MHz
WDW EM
SSB 0
LB 2.00 Hz
GB 0
PC 1.40

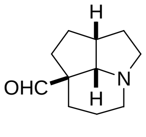

16

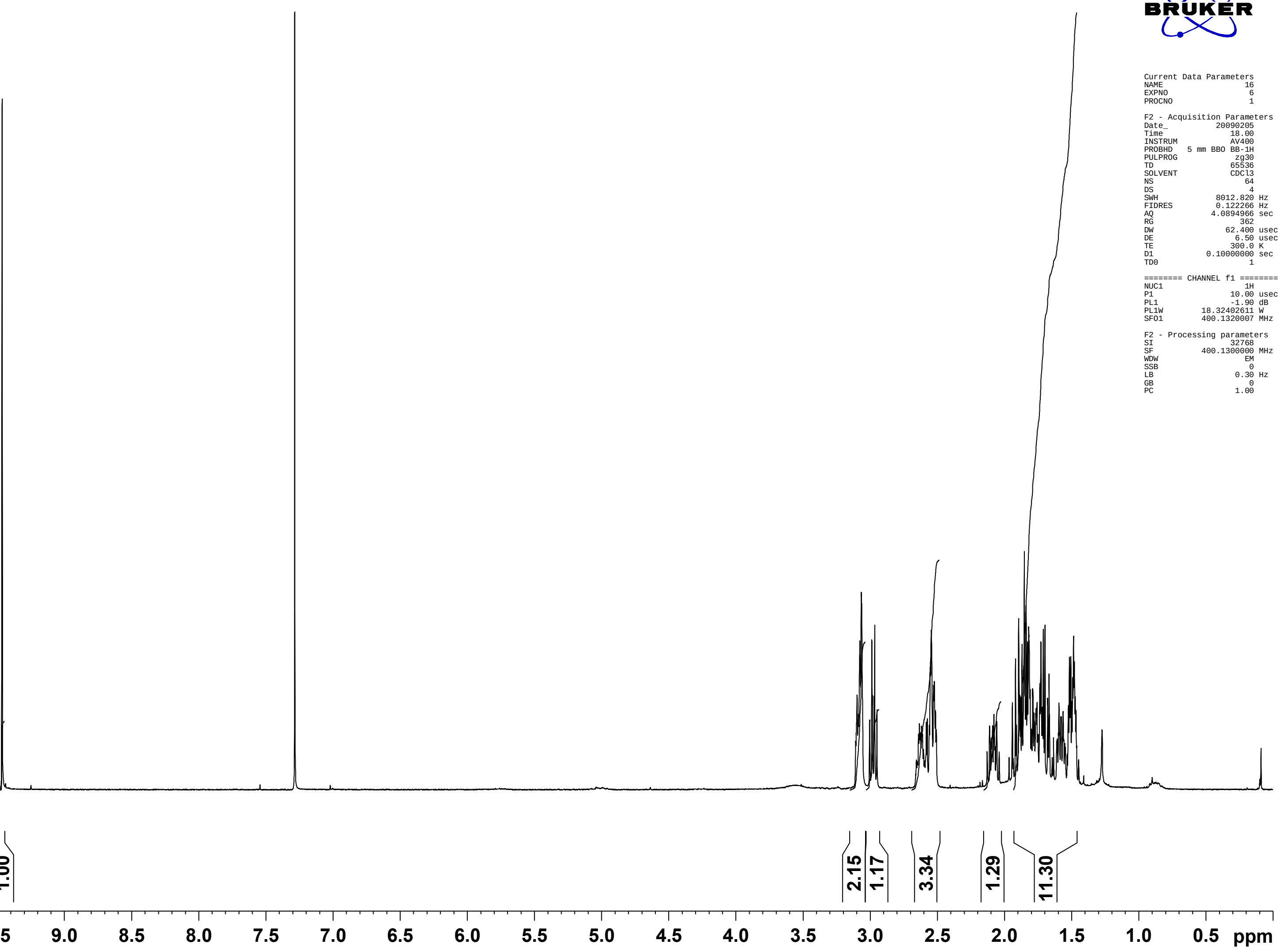
Current Data Parameters
NAME 16
EXPNO 6
PROCNO 1
F2 - Acquisition Parameters
Date_ 20090205
Time 18.00
INSTRUM AV400
PROBHD 5 mm BBO BB-1H
PULPROG zg30
TD 65536
SOLVENT CDCl3
NS 64
DS 4
SWH 8012.820 Hz
FIDRES 0.122266 Hz
AQ 4.0894966 sec
RG 362
DW 62.400 usec
DE 6.50 usec
TE 300.0 K
D1 0.10000000 sec
TD0 1
======== CHANNEL f1 ========
NUC1 1H
P1 10.00 usec
PL1 -1.90 dB
PL1W 18.32402611 W
SFO1 400.1320007 MHz
F2 - Processing parameters
SI 32768
SF 400.1300000 MHz
WDW EM
SSB 0
LB 0.30 Hz
GB 0
PC 1.00
1.00
2.15
1.17
3.34
1.29
11.30
10.0
9.5
9.0
8.5
8.0
7.5
7.0
6.5
6.0
5.5
5.0
4.5
4.0
3.5
3.0
2.5
2.0
1.5
1.0
0.5
ppm

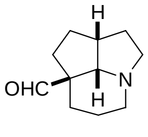

16

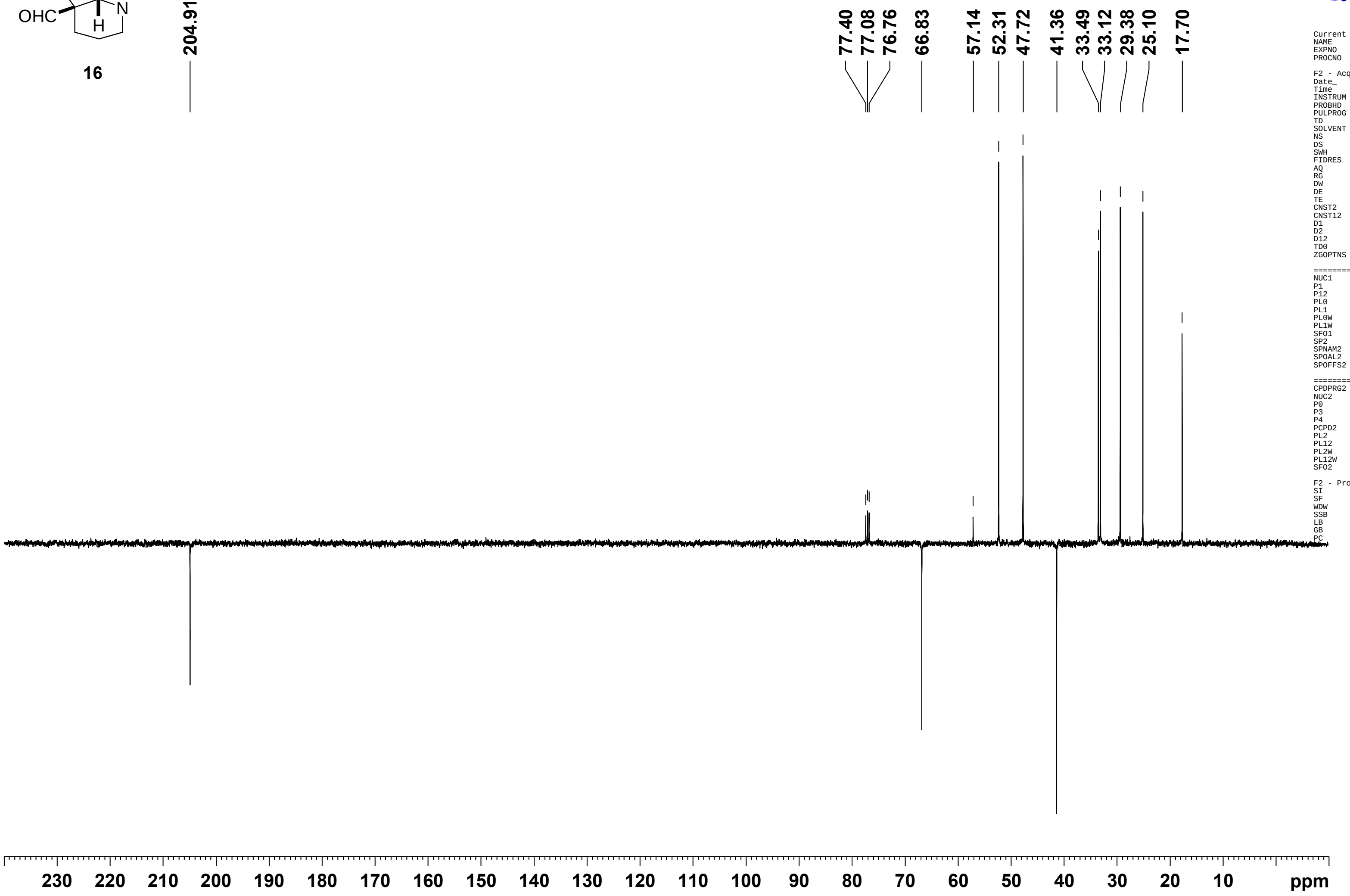


```
Current Data Parameters
NAME                    16
EXPNO                    5
PROCNO                   1

F2 - Acquisition Parameters
Date_             20090206
Time                 11.05
INSTRUM              AV400
PROBHD   5 mm BBO BB-1H
PULPROG            deptqsp
TD                   65536
SOLVENT              CDCl3
NS                     128
DS                       4
SWH              25125.629 Hz
FIDRES            0.383387 Hz
AQ               1.3042164 sec
RG                   16384
DW                  19.900 usec
DE                    6.50 usec
TE                   300.0 K
CNST2          145.0000000
CNST12           1.5000000
D1              2.00000000 sec
D2              0.00344828 sec
D12             0.00002000 sec
TD0                      1
ZGOPTNS

======== CHANNEL f1 ========
NUC1                   13C
P1                    8.00 usec
P12                2000.00 usec
PL0                 120.00 dB
PL1                  -3.10 dB
PL0W            0.00000000 W
PL1W           58.97905731 W
SFO1           100.6243395 MHz
SP2                   7.00 dB
SPNAM2         Crp60comp.4
SPOAL2               0.500
SPOFFS2               0.00 Hz

======== CHANNEL f2 ========
CPDPRG2            waltz16
NUC2                    1H
P0                   15.00 usec
P3                   10.00 usec
P4                   20.00 usec
PCPD2                88.00 usec
PL2                  -1.90 dB
PL12                 16.99 dB
PL2W           18.32402611 W
PL12W           0.23660338 W
SFO2           400.1316005 MHz

F2 - Processing parameters
SI                   32768
SF             100.6127690 MHz
WDW                     EM
SSB                      0
LB                    2.00 Hz
GB                       0
PC                    1.40
```

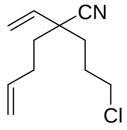
CN
Cl

17

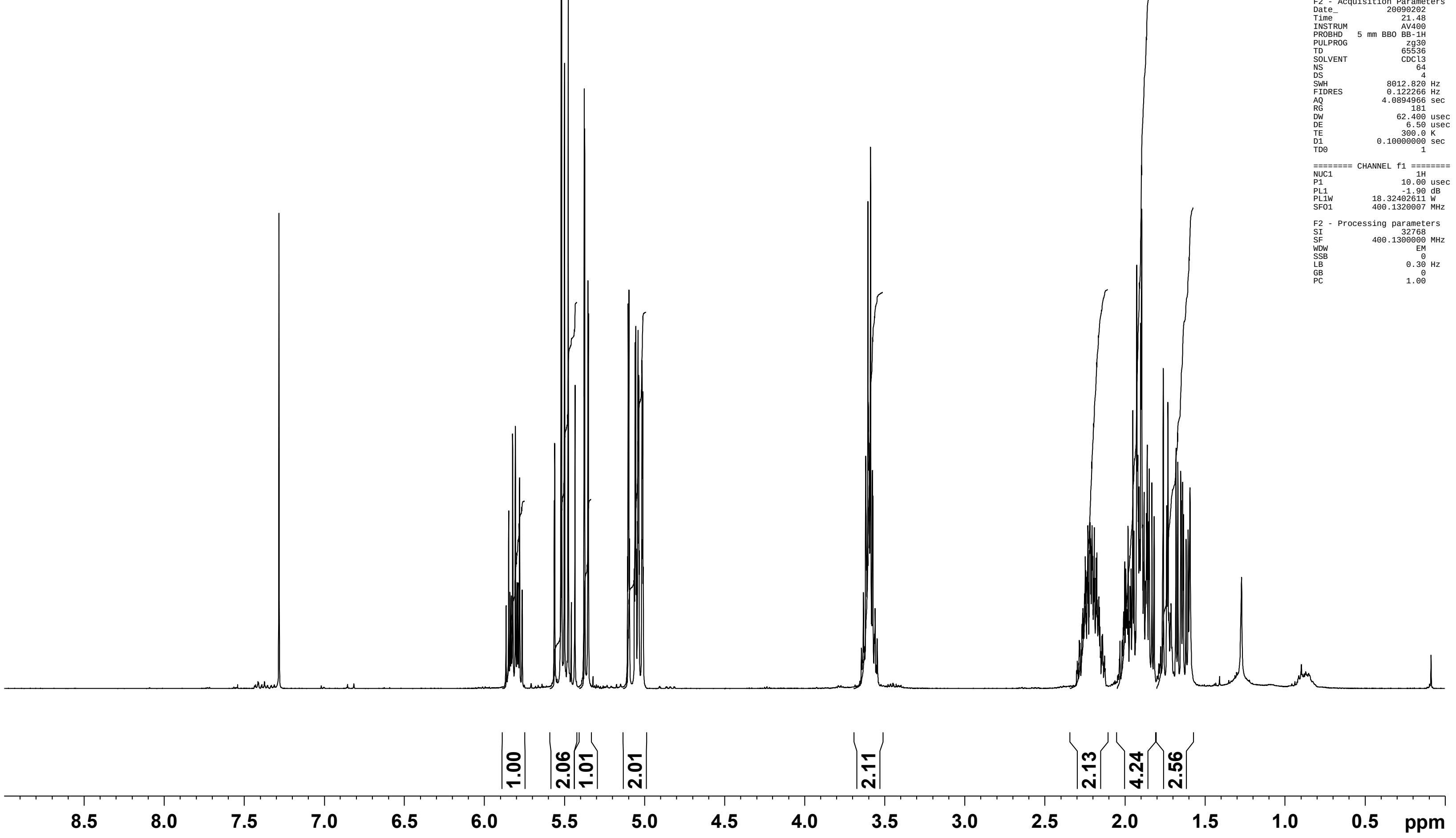
BRUKER
Current Data Parameters
NAME 17
EXPNO 7
PROCNO 1
F2 - Acquisition Parameters
Date_ 20090202
Time 21.48
INSTRUM AV400
PROBHD 5 mm BBO BB-1H
PULPROG zg30
TD 65536
SOLVENT CDCl3
NS 64
DS 4
SWH 8012.820 Hz
FIDRES 0.122266 Hz
AQ 4.0894966 sec
RG 181
DW 62.400 usec
DE 6.50 usec
TE 300.0 K
D1 0.10000000 sec
TD0 1
======== CHANNEL f1 ========
NUC1 1H
P1 10.00 usec
PL1 -1.90 dB
PL1W 18.32402611 W
SFO1 400.1320007 MHz
F2 - Processing parameters
SI 32768
SF 400.1300000 MHz
WDW EM
SSB 0
LB 0.30 Hz
GB 0
PC 1.00
1.00
2.06
1.01
2.01
2.11
2.13
4.24
2.56
8.5
8.0
7.5
7.0
6.5
6.0
5.5
5.0
4.5
4.0
3.5
3.0
2.5
2.0
1.5
1.0
0.5
ppm

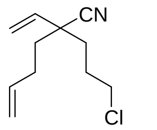

17

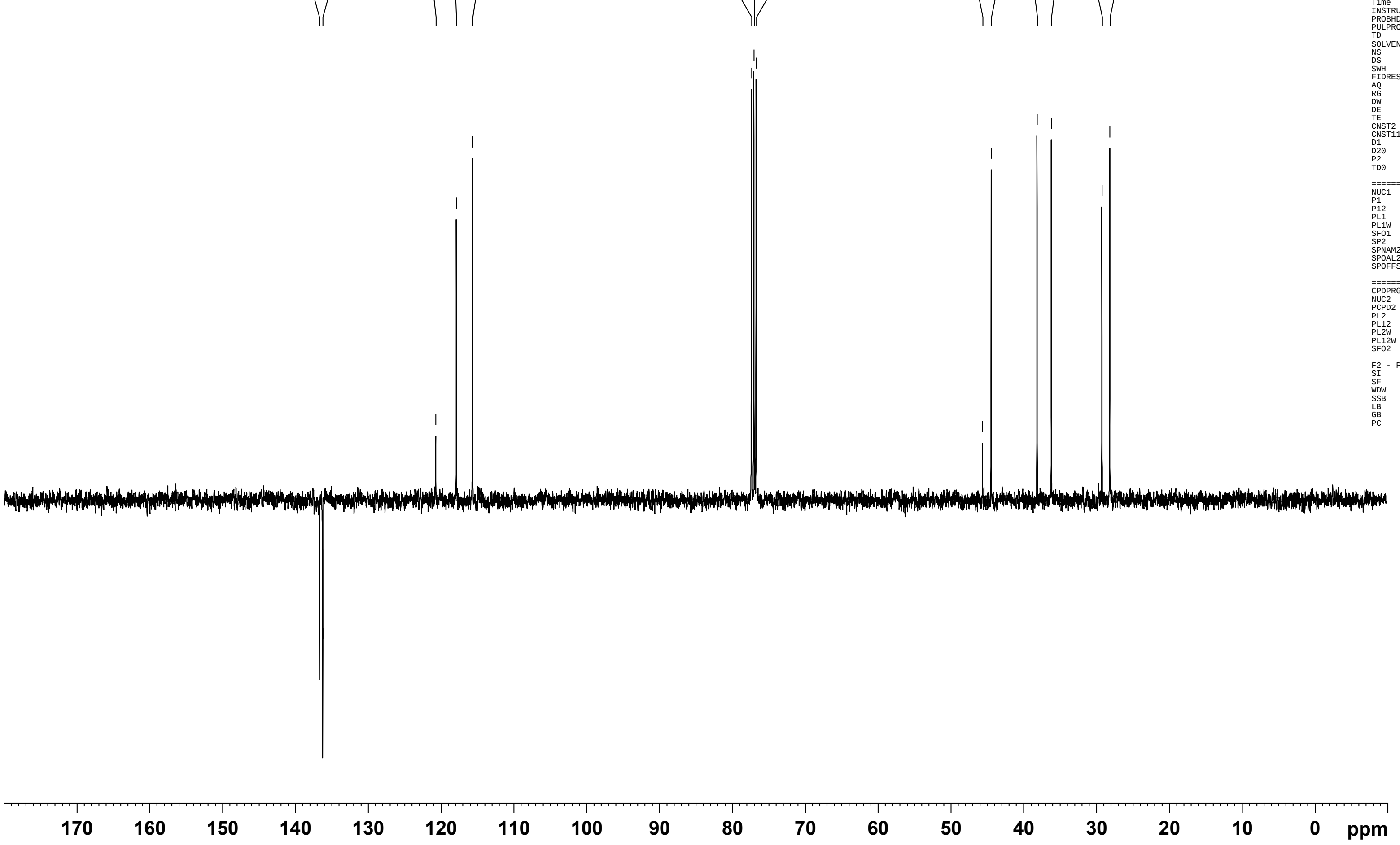


```
Current Data Parameters
NAME                  17
EXPNO                  8
PROCNO                 1

F2 - Acquisition Parameters
Date_           20090202
Time               22.35
INSTRUM            AV400
PROBHD   5 mm BBO BB-1H
PULPROG           jmodsp
TD                 65536
SOLVENT            CDCl3
NS                   512
DS                     4
SWH            25125.629 Hz
FIDRES          0.383387 Hz
AQ             1.3042164 sec
RG                 16384
DW                19.900 usec
DE                  6.50 usec
TE                 300.0 K
CNST2        145.0000000
CNST11         1.0000000
D1            4.00000000 sec
D20           0.00689655 sec
P2                 16.00 usec
TD0                    1

======== CHANNEL f1 ========
NUC1                 13C
P1                  8.00 usec
P12              2000.00 usec
PL1                -3.10 dB
PL1W         58.97905731 W
SFO1         100.6243395 MHz
SP2                 7.00 dB
SPNAM2       Crp60comp.4
SPOAL2             0.500
SPOFFS2             0.00 Hz

======== CHANNEL f2 ========
CPDPRG2          waltz16
NUC2                  1H
PCPD2              88.00 usec
PL2                -1.90 dB
PL12               16.99 dB
PL2W         18.32402611 W
PL12W         0.23660338 W
SFO2         400.1316005 MHz

F2 - Processing parameters
SI                 32768
SF           100.6127690 MHz
WDW                   EM
SSB                    0
LB                  2.00 Hz
GB                     0
PC                  1.40
```

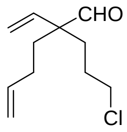
CHO
Cl

18

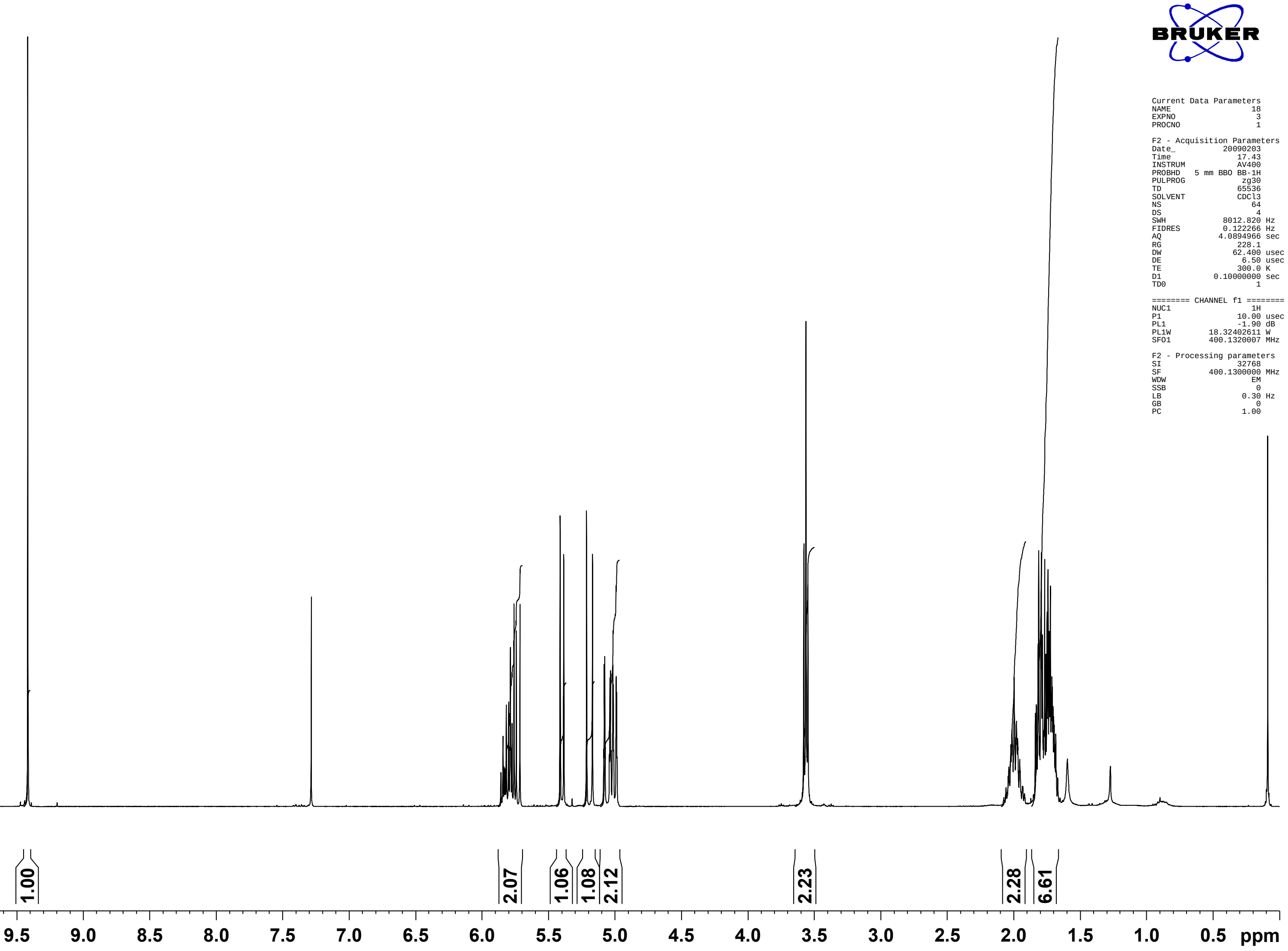
BRUKER
Current Data Parameters
NAME 18
EXPNO 3
PROCNO 1
F2 - Acquisition Parameters
Date_ 20090203
Time 17.43
INSTRUM AV400
PROBHD 5 mm BBO BB-1H
PULPROG zg30
TD 65536
SOLVENT CDCl3
NS 64
DS 4
SWH 8012.820 Hz
FIDRES 0.122266 Hz
AQ 4.0894966 sec
RG 228.1
DW 62.400 usec
DE 6.50 usec
TE 300.0 K
D1 0.10000000 sec
TD0 1
======== CHANNEL f1 ========
NUC1 1H
P1 10.00 usec
PL1 -1.90 dB
PL1W 18.32402611 W
SFO1 400.1320007 MHz
F2 - Processing parameters
SI 32768
SF 400.1300000 MHz
WDW EM
SSB 0
LB 0.30 Hz
GB 0
PC 1.00
1.00
2.07
1.06
1.08
2.12
2.23
2.28
6.61
10.0
9.5
9.0
8.5
8.0
7.5
7.0
6.5
6.0
5.5
5.0
4.5
4.0
3.5
3.0
2.5
2.0
1.5
1.0
0.5
ppm

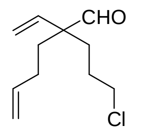

18

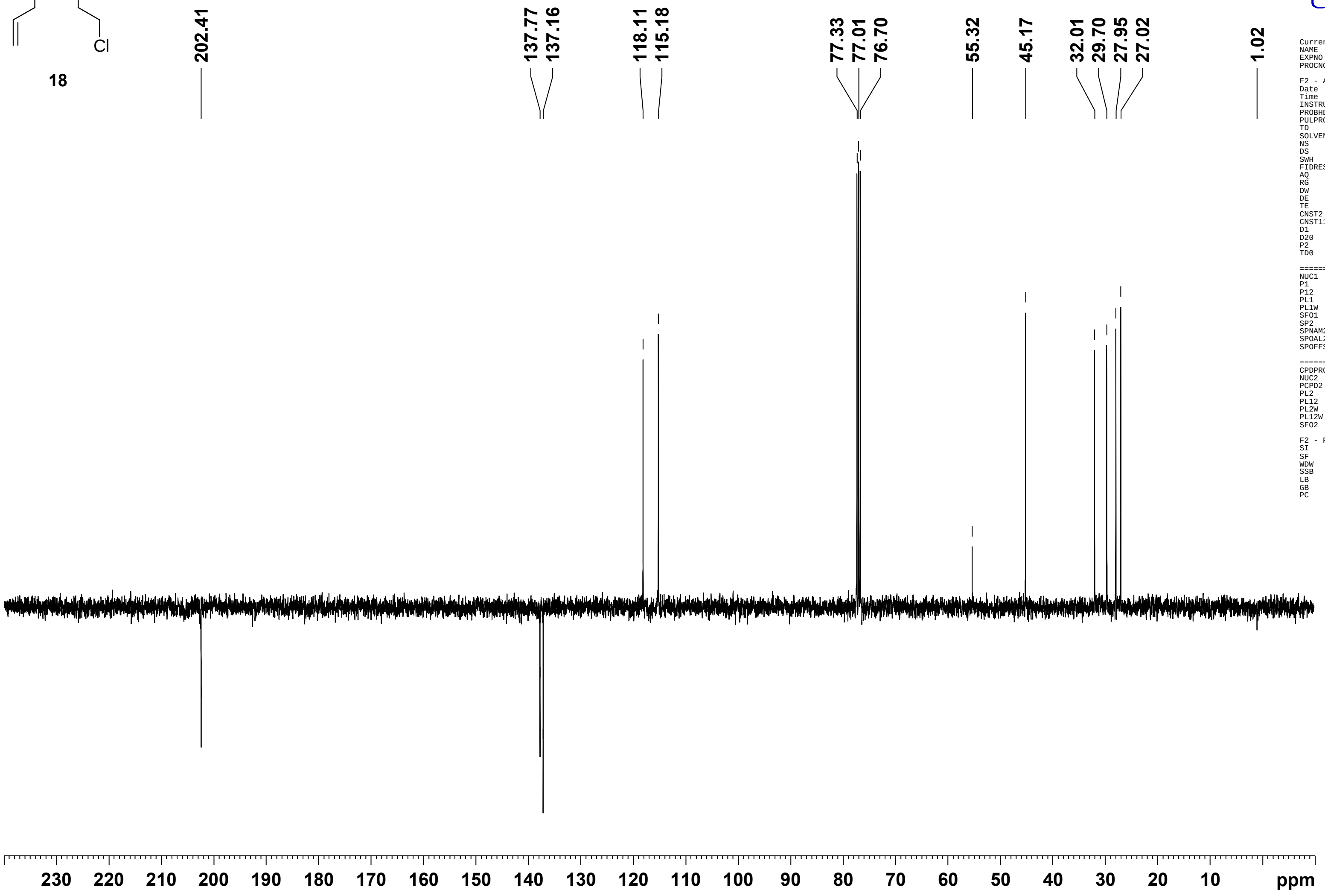


```
Current Data Parameters
NAME                     18
EXPNO                     4
PROCNO                    1

F2 - Acquisition Parameters
Date_              20090203
Time                  18.30
INSTRUM               AV400
PROBHD   5 mm BBO BB-1H
PULPROG              jmodsp
TD                    65536
SOLVENT               CDCl3
NS                      512
DS                        4
SWH               25125.629 Hz
FIDRES             0.383387 Hz
AQ                1.3042164 sec
RG                    16384
DW                   19.900 usec
DE                     6.50 usec
TE                    300.0 K
CNST2           145.0000000
CNST11            1.0000000
D1               4.00000000 sec
D20              0.00689655 sec
P2                    16.00 usec
TD0                       1

======== CHANNEL f1 ========
NUC1                    13C
P1                     8.00 usec
P12                 2000.00 usec
PL1                   -3.10 dB
PL1W            58.97905731 W
SFO1            100.6243395 MHz
SP2                    7.00 dB
SPNAM2          Crp60comp.4
SPOAL2                0.500
SPOFFS2                0.00 Hz

======== CHANNEL f2 ========
CPDPRG2             waltz16
NUC2                     1H
PCPD2                 88.00 usec
PL2                   -1.90 dB
PL12                  16.99 dB
PL2W            18.32402611 W
PL12W            0.23660338 W
SFO2            400.1316005 MHz

F2 - Processing parameters
SI                    32768
SF              100.6127690 MHz
WDW                      EM
SSB                       0
LB                     2.00 Hz
GB                        0
PC                     1.40
```

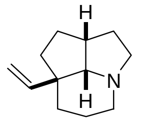

19

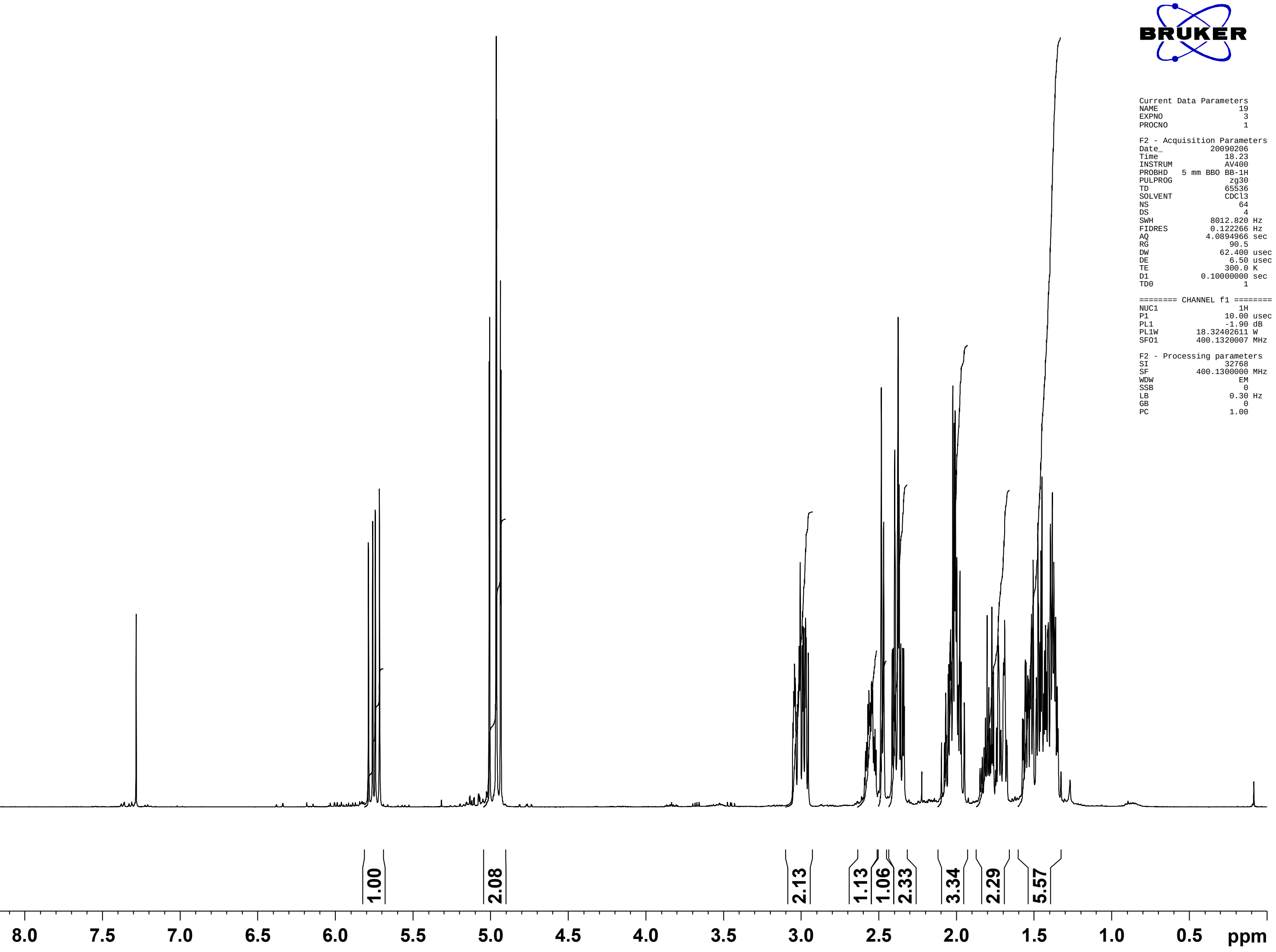


```
Current Data Parameters
NAME                 19
EXPNO                 3
PROCNO                1

F2 - Acquisition Parameters
Date_          20090206
Time              18.23
INSTRUM           AV400
PROBHD   5 mm BBO BB-1H
PULPROG            zg30
TD                65536
SOLVENT           CDCl3
NS                   64
DS                    4
SWH            8012.820 Hz
FIDRES         0.122266 Hz
AQ            4.0894966 sec
RG                 90.5
DW               62.400 usec
DE                 6.50 usec
TE                300.0 K
D1           0.10000000 sec
TD0                   1

======== CHANNEL f1 ========
NUC1                 1H
P1                10.00 usec
PL1               -1.90 dB
PL1W        18.32402611 W
SFO1        400.1320007 MHz

F2 - Processing parameters
SI                32768
SF          400.1300000 MHz
WDW                  EM
SSB                   0
LB                 0.30 Hz
GB                    0
PC                 1.00
```

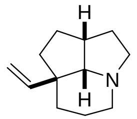

19

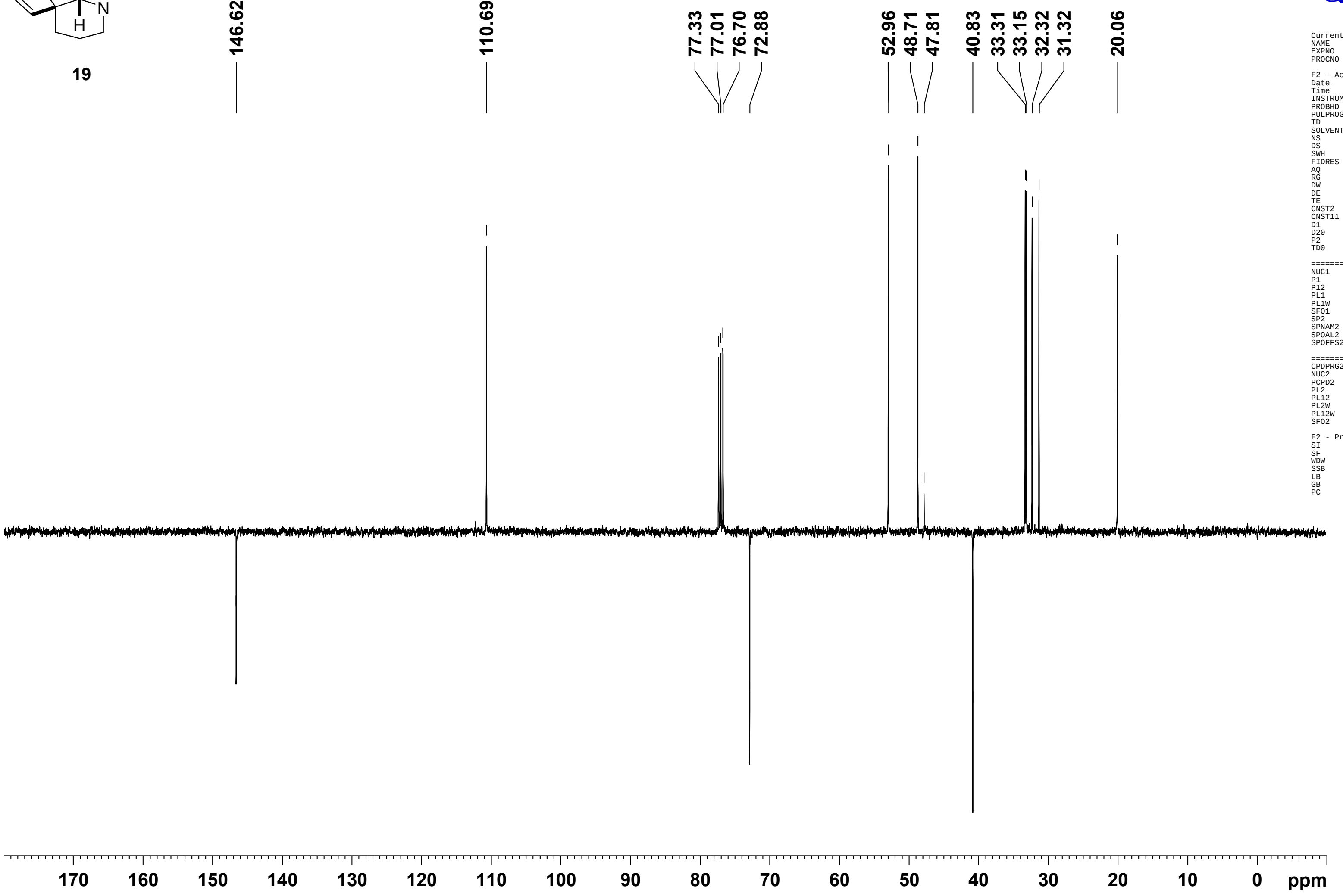

BRUKER

```
Current Data Parameters
NAME                    19
EXPNO                    4
PROCNO                   1

F2 - Acquisition Parameters
Date_             20090206
Time                 19.10
INSTRUM              AV400
PROBHD   5 mm BBO BB-1H
PULPROG            jmodsp
TD                   65536
SOLVENT              CDCl3
NS                     512
DS                       4
SWH              25125.629 Hz
FIDRES            0.383387 Hz
AQ               1.3042164 sec
RG                   16384
DW                  19.900 usec
DE                    6.50 usec
TE                   300.0 K
CNST2          145.0000000
CNST11           1.0000000
D1              4.00000000 sec
D20             0.00689655 sec
P2                   16.00 usec
TD0                      1

======== CHANNEL f1 ========
NUC1                   13C
P1                    8.00 usec
P12                2000.00 usec
PL1                  -3.10 dB
PL1W           58.97905731 W
SFO1           100.6243395 MHz
SP2                   7.00 dB
SPNAM2         Crp60comp.4
SPOAL2               0.500
SPOFFS2               0.00 Hz

======== CHANNEL f2 ========
CPDPRG2            waltz16
NUC2                    1H
PCPD2                88.00 usec
PL2                  -1.90 dB
PL12                 16.99 dB
PL2W           18.32402611 W
PL12W           0.23660338 W
SFO2           400.1316005 MHz

F2 - Processing parameters
SI                   32768
SF             100.6127690 MHz
WDW                     EM
SSB                      0
LB                    2.00 Hz
GB                       0
PC                    1.40
```

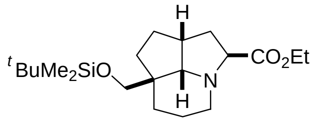

ᵗBuMe2SiO
H
H
N
CO2Et

20

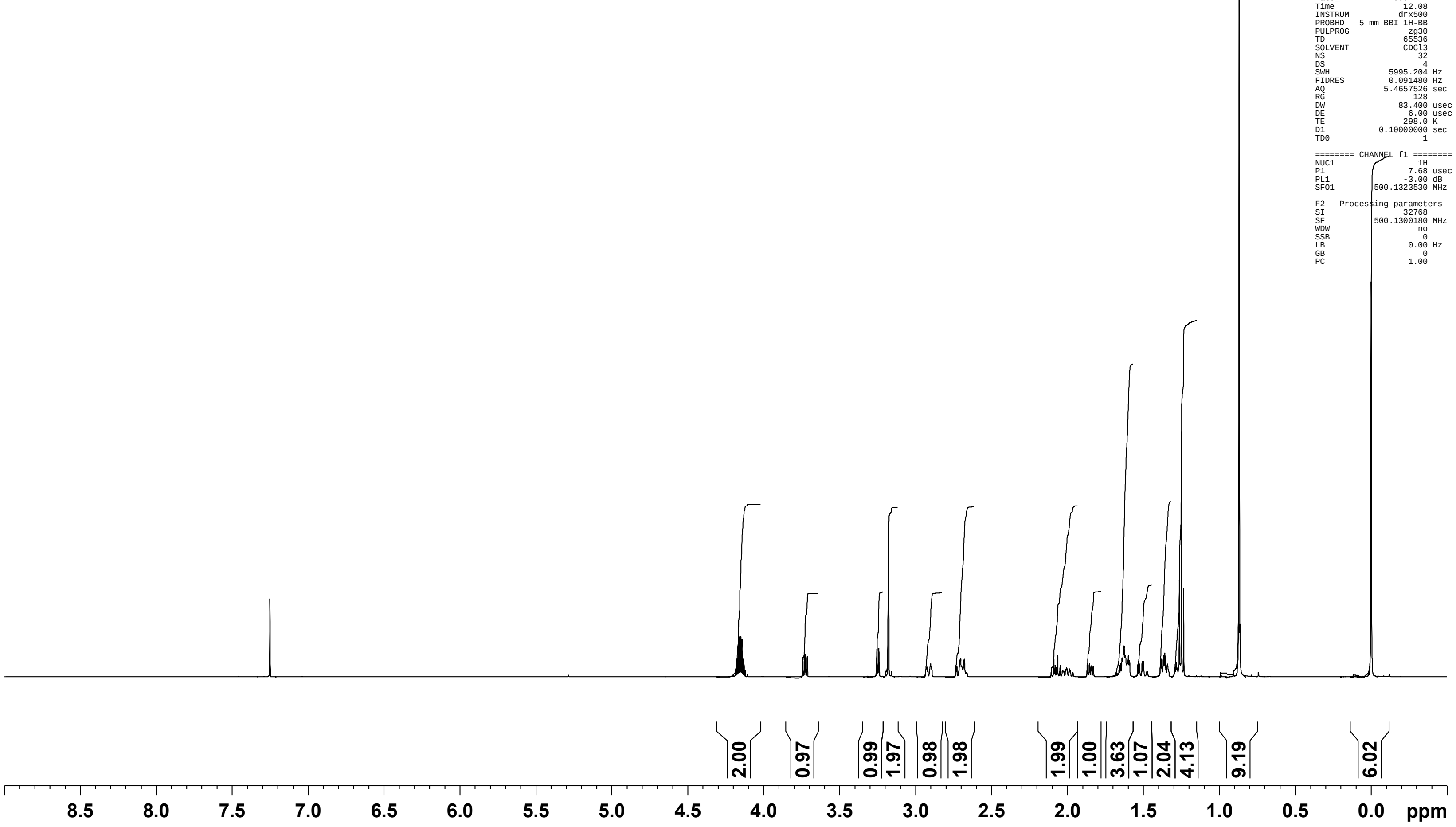

BRUKER
Current Data Parameters
NAME 20
EXPNO 500
PROCNO 1
F2 - Acquisition Parameters
Date_ 20091211
Time 12.08
INSTRUM drx500
PROBHD 5 mm BBI 1H-BB
PULPROG zg30
TD 65536
SOLVENT CDCl3
NS 32
DS 4
SWH 5995.204 Hz
FIDRES 0.091480 Hz
AQ 5.4657526 sec
RG 128
DW 83.400 usec
DE 6.00 usec
TE 298.0 K
D1 0.10000000 sec
TD0 1
======== CHANNEL f1 ========
NUC1 1H
P1 7.68 usec
PL1 -3.00 dB
SFO1 500.1323530 MHz
F2 - Processing parameters
SI 32768
SF 500.1300180 MHz
WDW no
SSB 0
LB 0.00 Hz
GB 0
PC 1.00
2.00
0.97
0.99
1.97
0.98
1.98
1.99
1.00
3.63
1.07
2.04
4.13
9.19
6.02
8.5
8.0
7.5
7.0
6.5
6.0
5.5
5.0
4.5
4.0
3.5
3.0
2.5
2.0
1.5
1.0
0.5
0.0
ppm

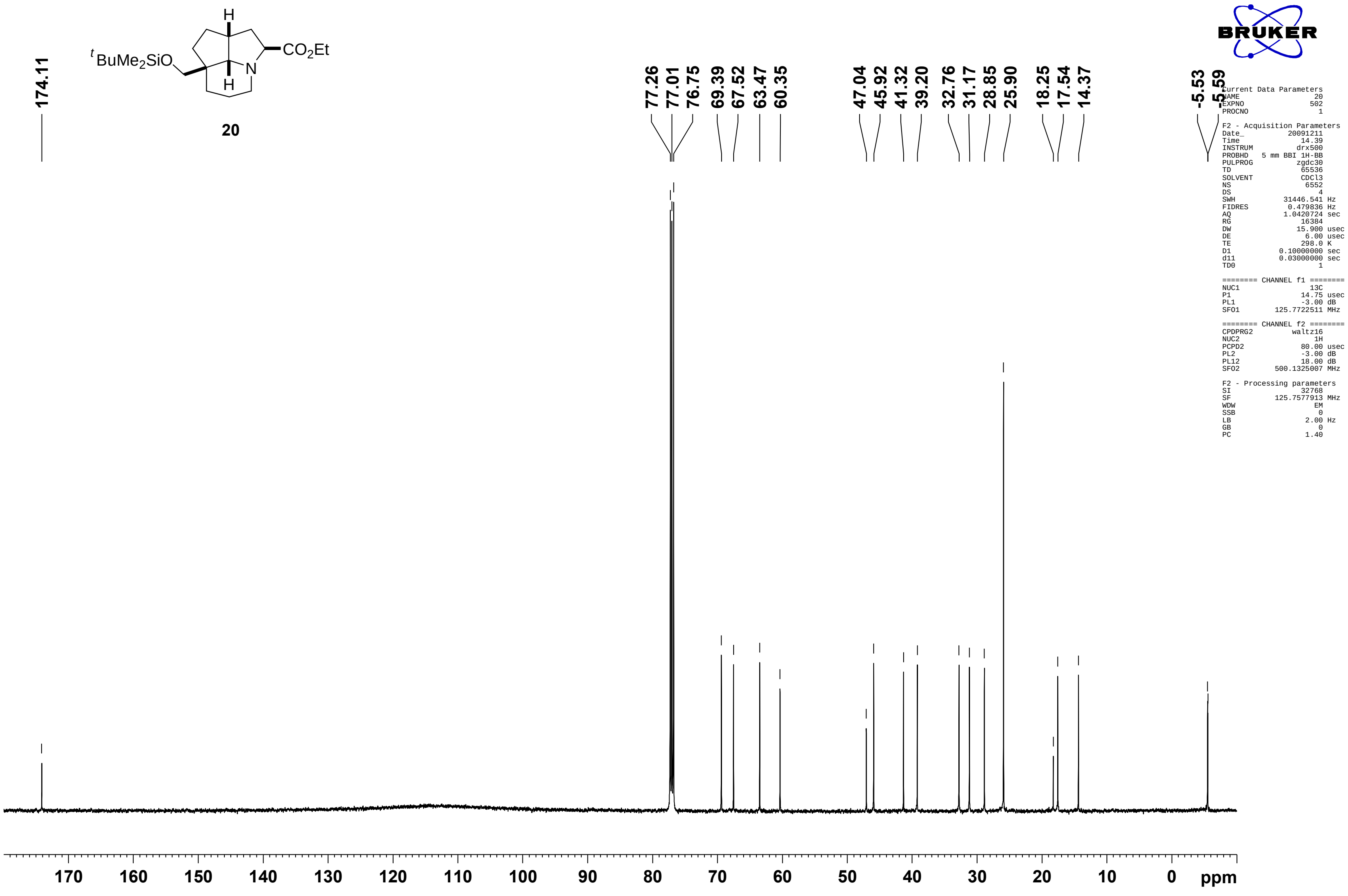

^{t}BuMe$_2$SiO
H
H
N
CO$_2$Et
174.11
77.26
77.01
76.75
69.39
67.52
63.47
60.35
47.04
45.92
41.32
39.20
32.76
31.17
28.85
25.90
18.25
17.54
14.37
-5.53
-5.59
BRUKER
Current Data Parameters
NAME 20
EXPNO 502
PROCNO 1
F2 - Acquisition Parameters
Date_ 20091211
Time 14.39
INSTRUM drx500
PROBHD 5 mm BBI 1H-BB
PULPROG zgdc30
TD 65536
SOLVENT CDCl3
NS 6552
DS 4
SWH 31446.541 Hz
FIDRES 0.479836 Hz
AQ 1.0420724 sec
RG 16384
DW 15.900 usec
DE 6.00 usec
TE 298.0 K
D1 0.10000000 sec
d11 0.03000000 sec
TD0 1
======== CHANNEL f1 ========
NUC1 13C
P1 14.75 usec
PL1 -3.00 dB
SFO1 125.7722511 MHz
======== CHANNEL f2 ========
CPDPRG2 waltz16
NUC2 1H
PCPD2 80.00 usec
PL2 -3.00 dB
PL12 18.00 dB
SFO2 500.1325007 MHz
F2 - Processing parameters
SI 32768
SF 125.7577913 MHz
WDW EM
SSB 0
LB 2.00 Hz
GB 0
PC 1.40
170 160 150 140 130 120 110 100 90 80 70 60 50 40 30 20 10 0 ppm

20

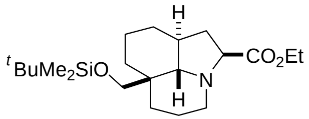

^{t}BuMe$_2$SiO
H
H
N
CO$_2$Et

21

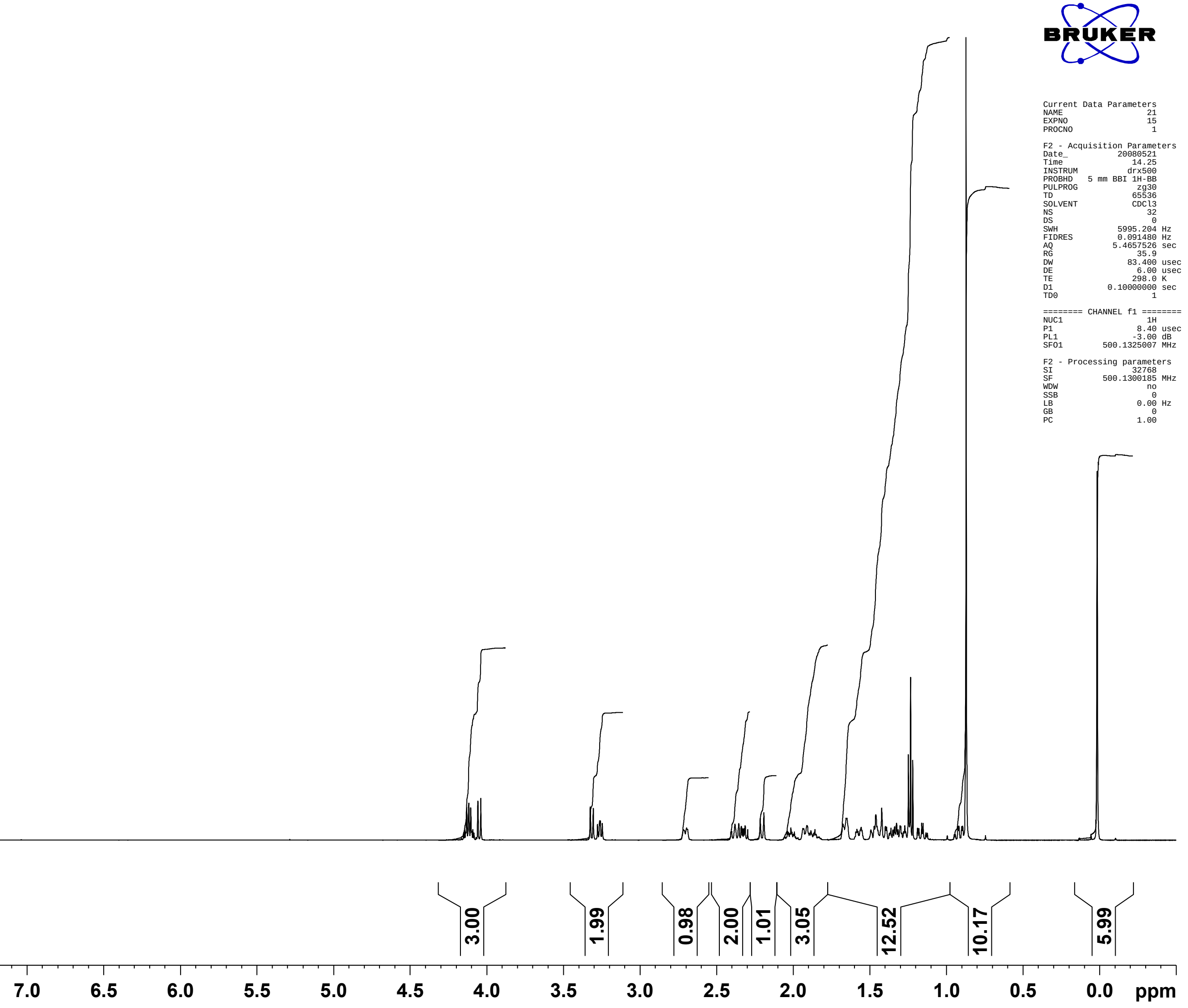

Current Data Parameters
NAME 21
EXPNO 15
PROCNO 1
F2 - Acquisition Parameters
Date_ 20080521
Time 14.25
INSTRUM drx500
PROBHD 5 mm BBI 1H-BB
PULPROG zg30
TD 65536
SOLVENT CDCl3
NS 32
DS 0
SWH 5995.204 Hz
FIDRES 0.091480 Hz
AQ 5.4657526 sec
RG 35.9
DW 83.400 usec
DE 6.00 usec
TE 298.0 K
D1 0.10000000 sec
TD0 1
======== CHANNEL f1 ========
NUC1 1H
P1 8.40 usec
PL1 -3.00 dB
SFO1 500.1325007 MHz
F2 - Processing parameters
SI 32768
SF 500.1300185 MHz
WDW no
SSB 0
LB 0.00 Hz
GB 0
PC 1.00
3.00
1.99
0.98
2.00
1.01
3.05
12.52
10.17
5.99
8.5
8.0
7.5
7.0
6.5
6.0
5.5
5.0
4.5
4.0
3.5
3.0
2.5
2.0
1.5
1.0
0.5
0.0
ppm

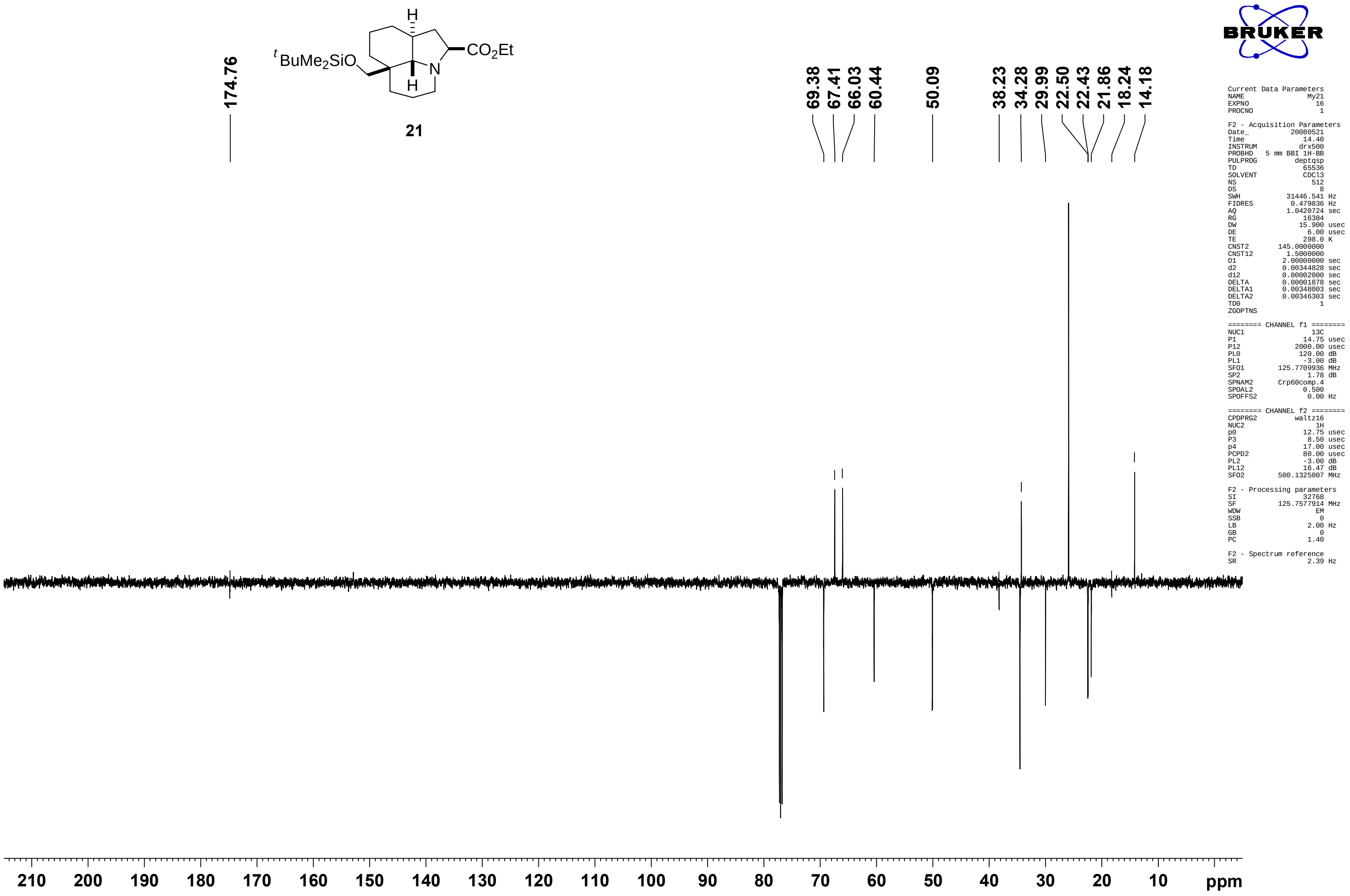

174.76
^{t}BuMe$_2$SiO
H
H
N
CO$_2$Et
21
69.38
67.41
66.03
60.44
50.09
38.23
34.28
29.99
22.50
22.43
21.86
18.24
14.18
BRUKER
Current Data Parameters
NAME My21
EXPNO 16
PROCNO 1
F2 - Acquisition Parameters
Date_ 20080521
Time 14.40
INSTRUM drx500
PROBHD 5 mm BBI 1H-BB
PULPROG deptqsp
TD 65536
SOLVENT CDCl3
NS 512
DS 8
SWH 31446.541 Hz
FIDRES 0.479836 Hz
AQ 1.0420724 sec
RG 16384
DW 15.900 usec
DE 6.00 usec
TE 298.0 K
CNST2 145.0000000
CNST12 1.5000000
D1 2.00000000 sec
d2 0.00344828 sec
d12 0.00002000 sec
DELTA 0.00001878 sec
DELTA1 0.00348003 sec
DELTA2 0.00346303 sec
TD0 1
ZGOPTNS
======== CHANNEL f1 ========
NUC1 13C
P1 14.75 usec
P12 2000.00 usec
PL0 120.00 dB
PL1 -3.00 dB
SFO1 125.7709936 MHz
SP2 1.78 dB
SPNAM2 Crp60comp.4
SPOAL2 0.500
SPOFFS2 0.00 Hz
======== CHANNEL f2 ========
CPDPRG2 waltz16
NUC2 1H
p0 12.75 usec
P3 8.50 usec
p4 17.00 usec
PCPD2 80.00 usec
PL2 -3.00 dB
PL12 16.47 dB
SFO2 500.1325007 MHz
F2 - Processing parameters
SI 32768
SF 125.7577914 MHz
WDW EM
SSB 0
LB 2.00 Hz
GB 0
PC 1.40
F2 - Spectrum reference
SR 2.39 Hz
210
200
190
180
170
160
150
140
130
120
110
100
90
80
70
60
50
40
30
20
10
ppm

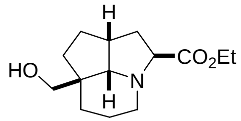

H
HO
CO2Et
N
H

24

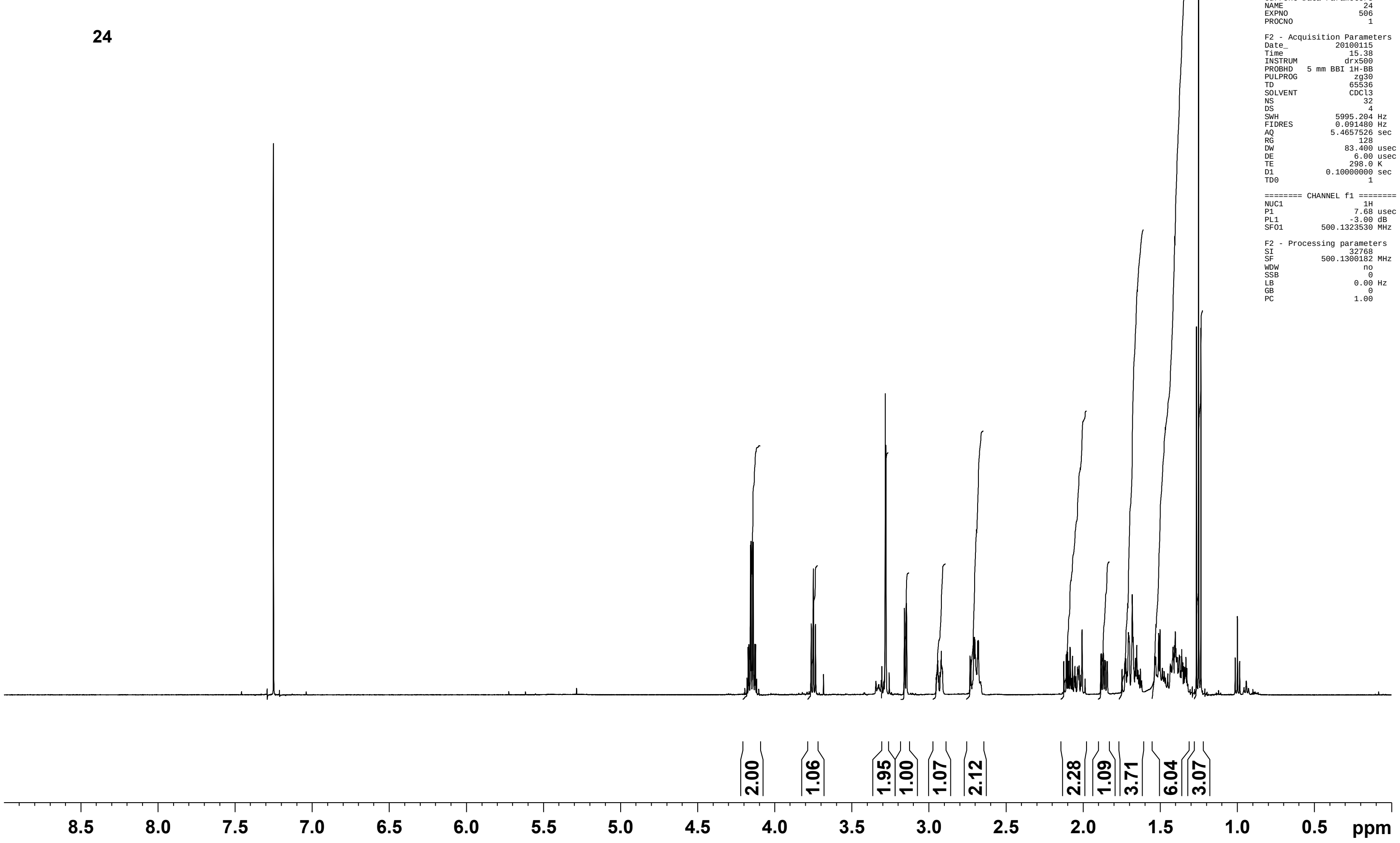

Current Data Parameters
NAME 24
EXPNO 506
PROCNO 1
F2 - Acquisition Parameters
Date_ 20100115
Time 15.38
INSTRUM drx500
PROBHD 5 mm BBI 1H-BB
PULPROG zg30
TD 65536
SOLVENT CDCl3
NS 32
DS 4
SWH 5995.204 Hz
FIDRES 0.091480 Hz
AQ 5.4657526 sec
RG 128
DW 83.400 usec
DE 6.00 usec
TE 298.0 K
D1 0.10000000 sec
TD0 1
======== CHANNEL f1 ========
NUC1 1H
P1 7.68 usec
PL1 -3.00 dB
SFO1 500.1323530 MHz
F2 - Processing parameters
SI 32768
SF 500.1300182 MHz
WDW no
SSB 0
LB 0.00 Hz
GB 0
PC 1.00
2.00
1.06
1.95
1.00
1.07
2.12
2.28
1.09
3.71
6.04
3.07
8.5
8.0
7.5
7.0
6.5
6.0
5.5
5.0
4.5
4.0
3.5
3.0
2.5
2.0
1.5
1.0
0.5
ppm

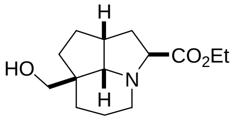

24

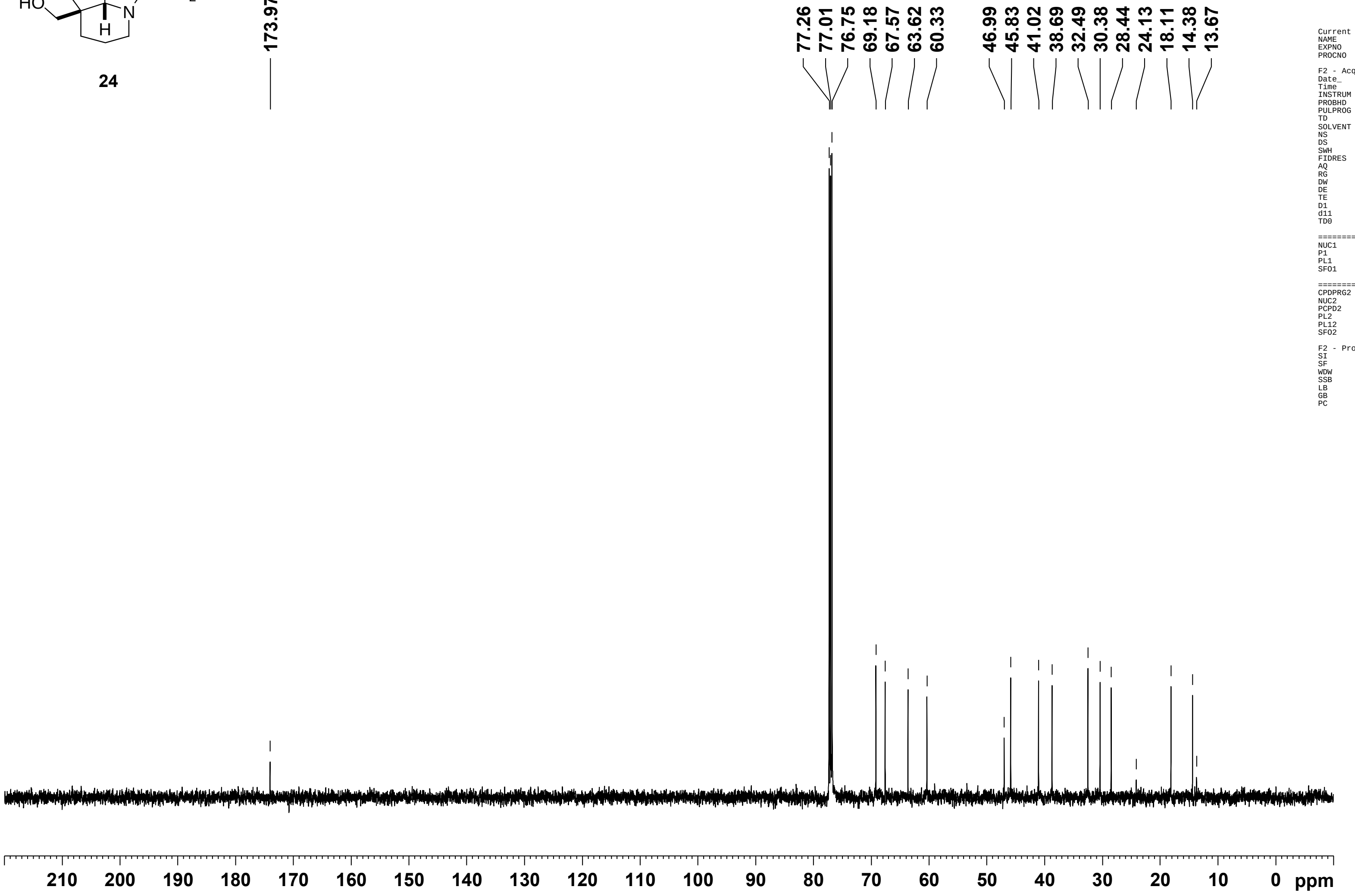


```
Current Data Parameters
NAME                 24
EXPNO               707
PROCNO                1

F2 - Acquisition Parameters
Date_          20100115
Time              16.11
INSTRUM          drx500
PROBHD   5 mm BBI 1H-BB
PULPROG          zgdc30
TD                65402
SOLVENT           CDCl3
NS                  747
DS                    4
SWH           31446.541 Hz
FIDRES         0.480819 Hz
AQ            1.0399418 sec
RG                16384
DW               15.900 usec
DE                 6.00 usec
TE                298.0 K
D1           0.10000000 sec
d11          0.03000000 sec
TD0                   1

======== CHANNEL f1 ========
NUC1                13C
P1                14.75 usec
PL1               -3.00 dB
SFO1        125.7722511 MHz

======== CHANNEL f2 ========
CPDPRG2         waltz16
NUC2                 1H
PCPD2             80.00 usec
PL2               -3.00 dB
PL12              18.00 dB
SFO2        500.1325007 MHz

F2 - Processing parameters
SI                32768
SF          125.7577910 MHz
WDW                  EM
SSB                   0
LB                 2.00 Hz
GB                    0
PC                 1.40
```

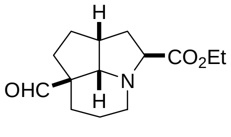
H
CO2Et
N
OHC
H

25

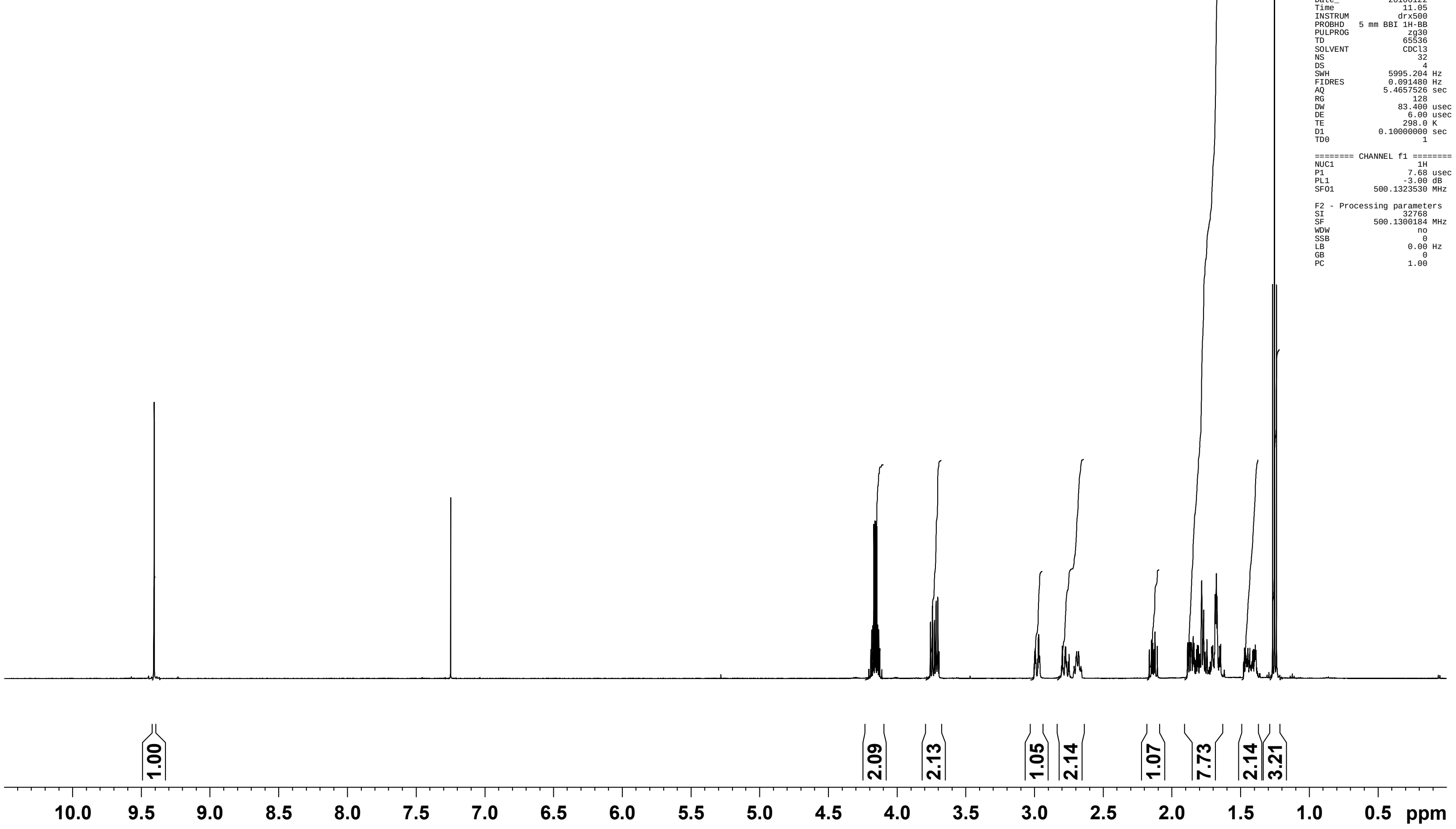
Current Data Parameters
NAME 25
EXPNO 500
PROCNO 1
F2 - Acquisition Parameters
Date_ 20100122
Time 11.05
INSTRUM drx500
PROBHD 5 mm BBI 1H-BB
PULPROG zg30
TD 65536
SOLVENT CDCl3
NS 32
DS 4
SWH 5995.204 Hz
FIDRES 0.091480 Hz
AQ 5.4657526 sec
RG 128
DW 83.400 usec
DE 6.00 usec
TE 298.0 K
D1 0.10000000 sec
TD0 1
======== CHANNEL f1 ========
NUC1 1H
P1 7.68 usec
PL1 -3.00 dB
SFO1 500.1323530 MHz
F2 - Processing parameters
SI 32768
SF 500.1300184 MHz
WDW no
SSB 0
LB 0.00 Hz
GB 0
PC 1.00
1.00
2.09
2.13
1.05
2.14
1.07
7.73
2.14
3.21
10.0
9.5
9.0
8.5
8.0
7.5
7.0
6.5
6.0
5.5
5.0
4.5
4.0
3.5
3.0
2.5
2.0
1.5
1.0
0.5
ppm

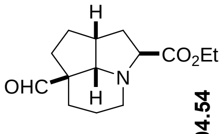

25

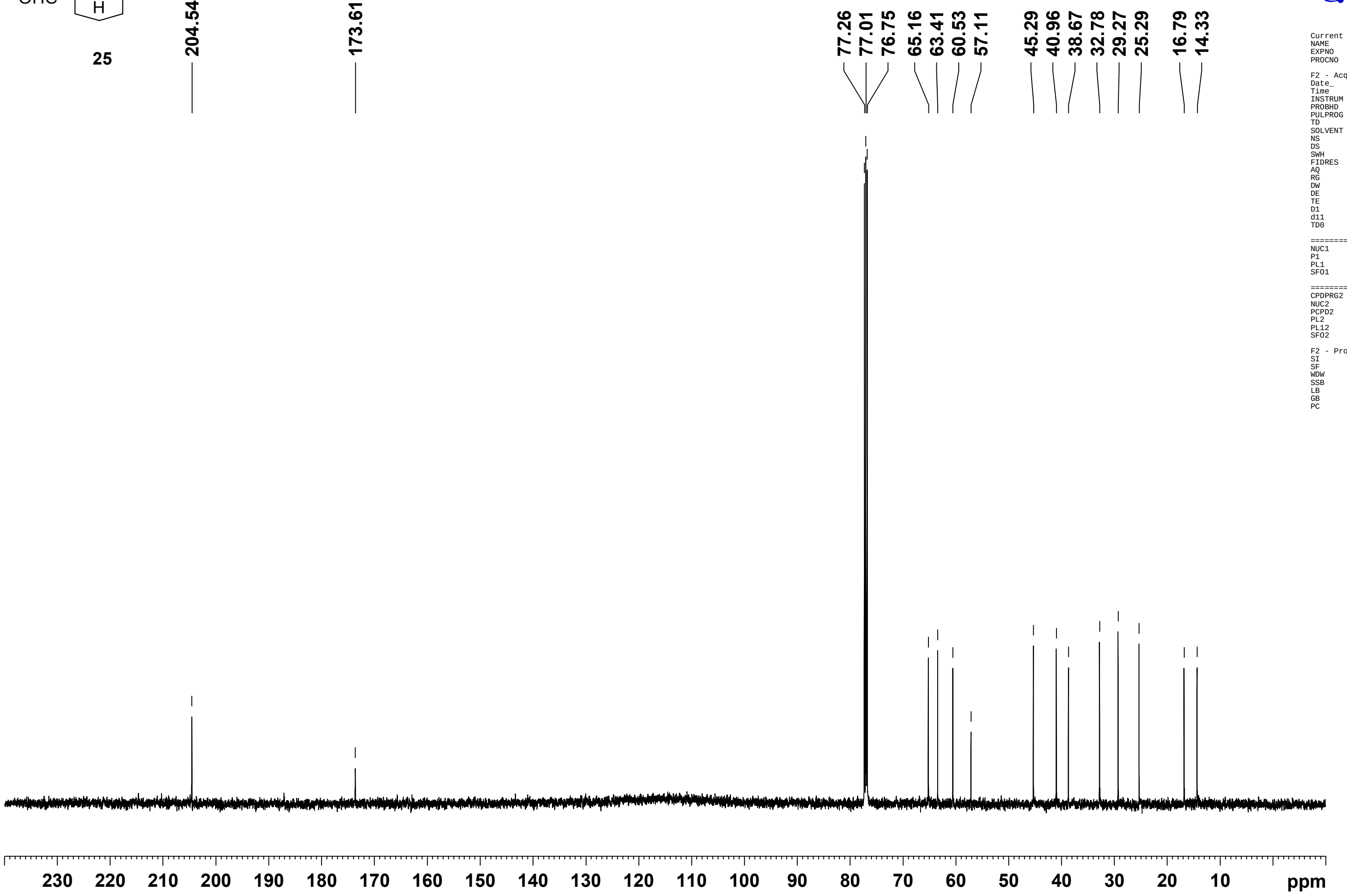

BRUKER

```
Current Data Parameters
NAME            jan22
EXPNO             503
PROCNO              1

F2 - Acquisition Parameters
Date_        20100122
Time            11.56
INSTRUM        drx500
PROBHD   5 mm BBI 1H-BB
PULPROG        zgdc30
TD              65536
SOLVENT         CDCl3
NS                765
DS                  4
SWH         31446.541 Hz
FIDRES       0.479836 Hz
AQ          1.0420724 sec
RG              16384
DW             15.900 usec
DE               6.00 usec
TE              297.9 K
D1         0.10000000 sec
d11        0.03000000 sec
TD0                 1

======== CHANNEL f1 ========
NUC1              13C
P1              14.75 usec
PL1             -3.00 dB
SFO1      125.7722511 MHz

======== CHANNEL f2 ========
CPDPRG2       waltz16
NUC2               1H
PCPD2           80.00 usec
PL2             -3.00 dB
PL12            18.00 dB
SFO2      500.1325007 MHz

F2 - Processing parameters
SI              32768
SF        125.7577919 MHz
WDW                EM
SSB                 0
LB               2.00 Hz
GB                  0
PC               1.40
```

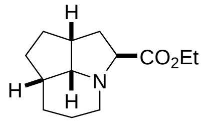
H
CO2Et
H
N
H

26

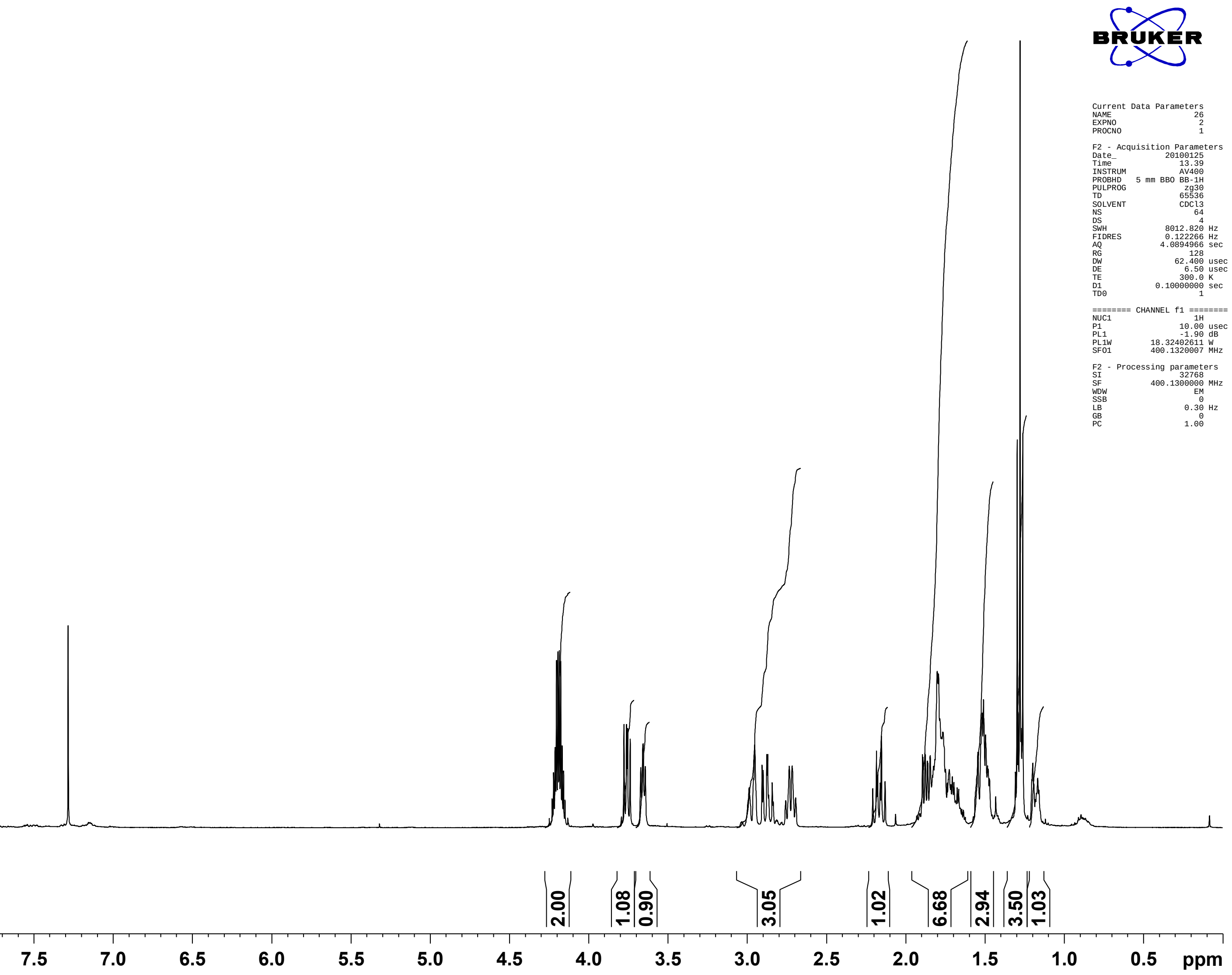
BRUKER
Current Data Parameters
NAME 26
EXPNO 2
PROCNO 1
F2 - Acquisition Parameters
Date_ 20100125
Time 13.39
INSTRUM AV400
PROBHD 5 mm BBO BB-1H
PULPROG zg30
TD 65536
SOLVENT CDCl3
NS 64
DS 4
SWH 8012.820 Hz
FIDRES 0.122266 Hz
AQ 4.0894966 sec
RG 128
DW 62.400 usec
DE 6.50 usec
TE 300.0 K
D1 0.10000000 sec
TD0 1
======== CHANNEL f1 ========
NUC1 1H
P1 10.00 usec
PL1 -1.90 dB
PL1W 18.32402611 W
SFO1 400.1320007 MHz
F2 - Processing parameters
SI 32768
SF 400.1300000 MHz
WDW EM
SSB 0
LB 0.30 Hz
GB 0
PC 1.00
2.00
1.08
0.90
3.05
1.02
6.68
2.94
3.50
1.03
8.5
8.0
7.5
7.0
6.5
6.0
5.5
5.0
4.5
4.0
3.5
3.0
2.5
2.0
1.5
1.0
0.5
ppm

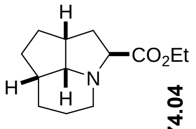

26

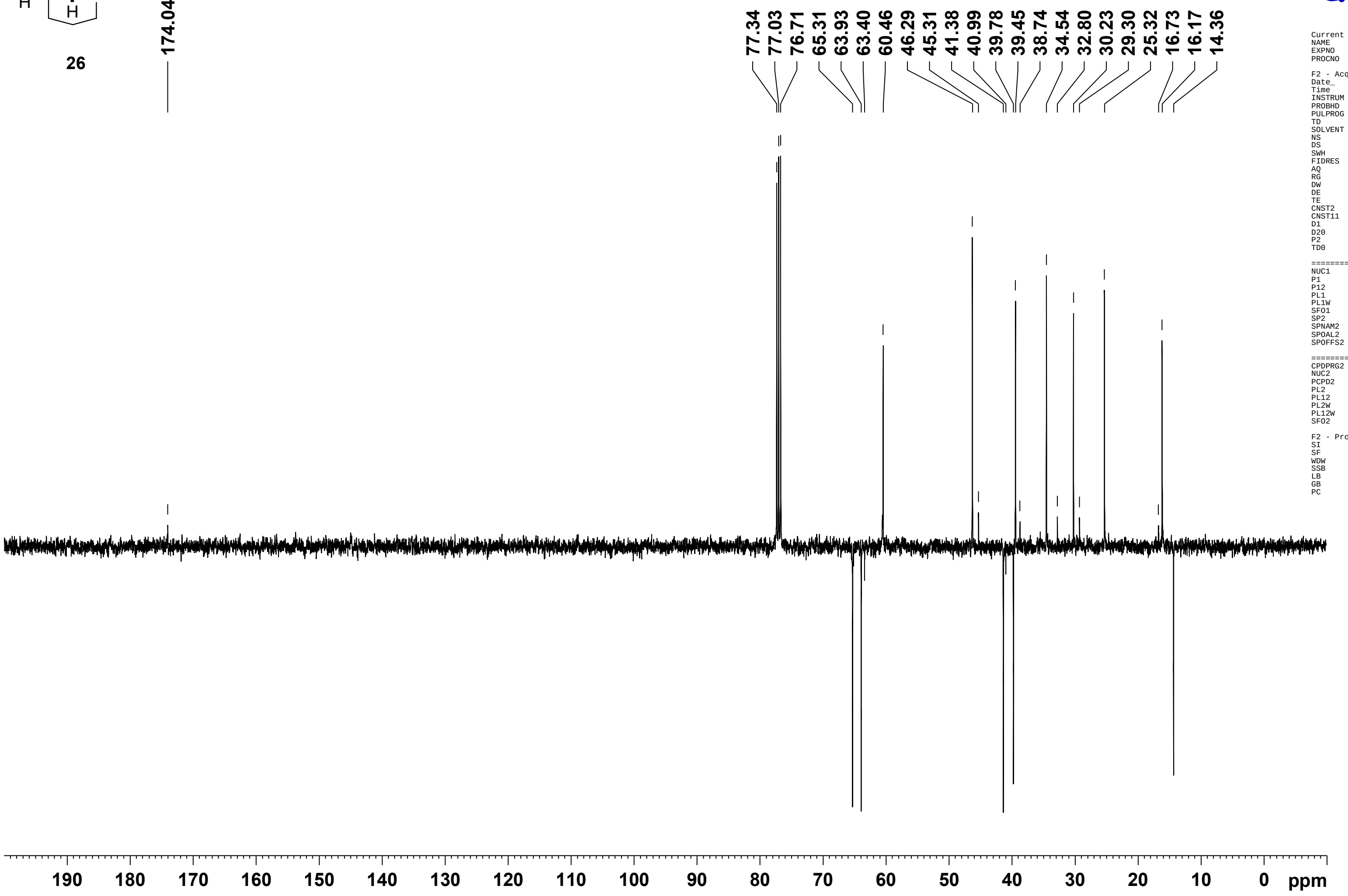


```
Current Data Parameters
NAME                 26
EXPNO                 5
PROCNO                1

F2 - Acquisition Parameters
Date_          20100125
Time              20.52
INSTRUM           AV400
PROBHD   5 mm BBO BB-1H
PULPROG         jmodsp
TD                65536
SOLVENT           CDCl3
NS                  512
DS                    4
SWH           25125.629 Hz
FIDRES         0.383387 Hz
AQ            1.3042164 sec
RG                16384
DW               19.900 usec
DE                 6.50 usec
TE                300.0 K
CNST2       145.0000000
CNST11        1.0000000
D1           4.00000000 sec
D20          0.00689655 sec
P2                16.00 usec
TD0                   1

======== CHANNEL f1 ========
NUC1                13C
P1                 8.00 usec
P12             2000.00 usec
PL1               -3.10 dB
PL1W        58.97905731 W
SFO1        100.6243395 MHz
SP2                7.00 dB
SPNAM2      Crp60comp.4
SPOAL2            0.500
SPOFFS2            0.00 Hz

======== CHANNEL f2 ========
CPDPRG2        waltz16
NUC2                 1H
PCPD2             88.00 usec
PL2               -1.90 dB
PL12              16.99 dB
PL2W        18.32402611 W
PL12W        0.23660338 W
SFO2        400.1316005 MHz

F2 - Processing parameters
SI                32768
SF          100.6127690 MHz
WDW                  EM
SSB                   0
LB                 2.00 Hz
GB                    0
PC                 1.40
```

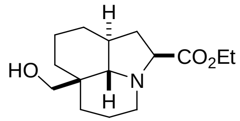
HO
H
CO2Et
N
H

27

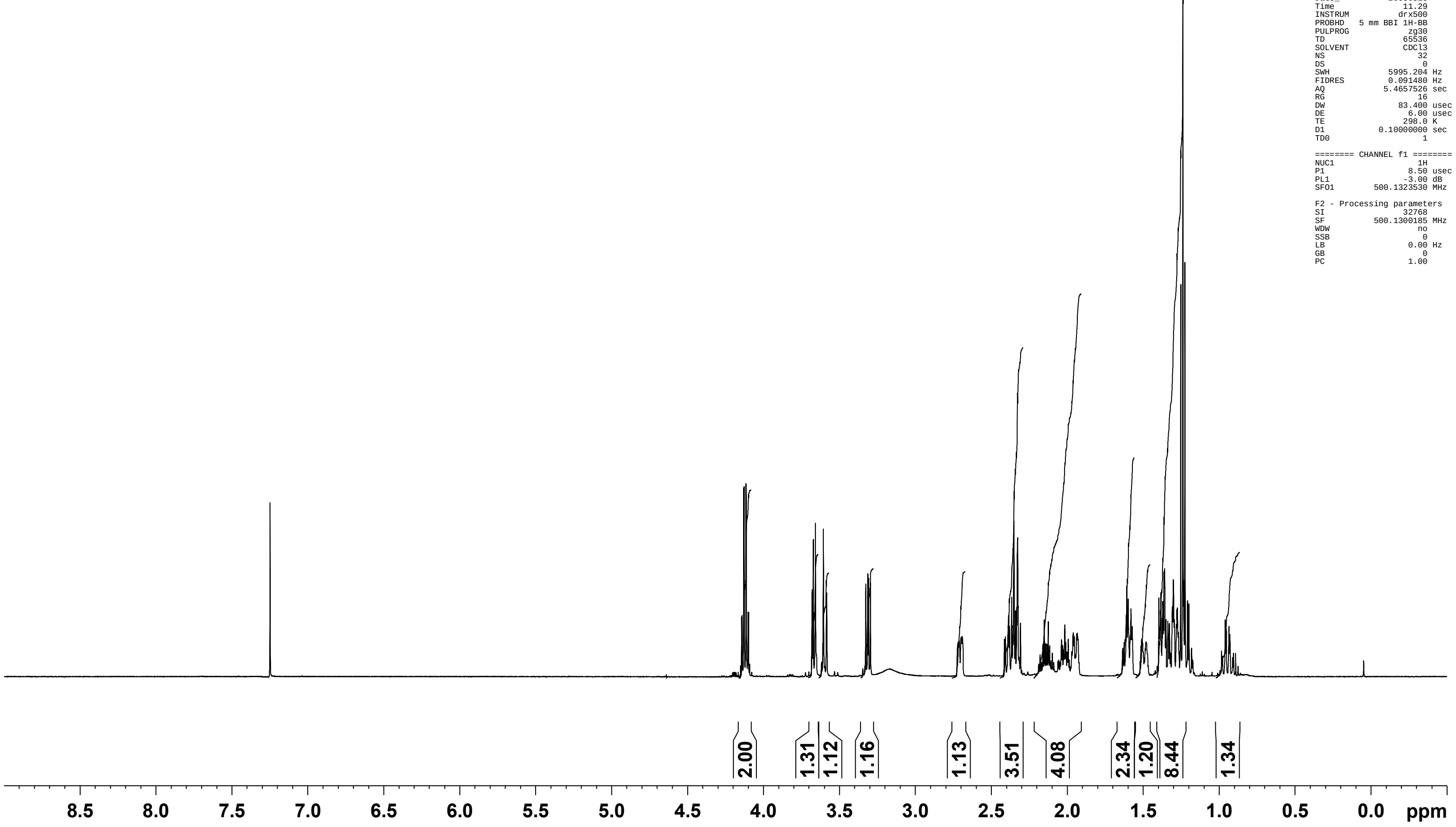
Current Data Parameters
NAME 27
EXPNO 1
PROCNO 1
F2 - Acquisition Parameters
Date_ 20080513
Time 11.29
INSTRUM drx500
PROBHD 5 mm BBI 1H-BB
PULPROG zg30
TD 65536
SOLVENT CDCl3
NS 32
DS 0
SWH 5995.204 Hz
FIDRES 0.091480 Hz
AQ 5.4657526 sec
RG 16
DW 83.400 usec
DE 6.00 usec
TE 298.0 K
D1 0.10000000 sec
TD0 1
======== CHANNEL f1 ========
NUC1 1H
P1 8.50 usec
PL1 -3.00 dB
SFO1 500.1323530 MHz
F2 - Processing parameters
SI 32768
SF 500.1300185 MHz
WDW no
SSB 0
LB 0.00 Hz
GB 0
PC 1.00
2.00
1.31
1.12
1.16
1.13
3.51
4.08
2.34
1.20
8.44
1.34
8.5
8.0
7.5
7.0
6.5
6.0
5.5
5.0
4.5
4.0
3.5
3.0
2.5
2.0
1.5
1.0
0.5
0.0
ppm

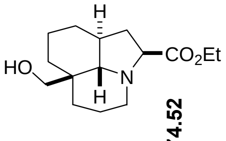

27

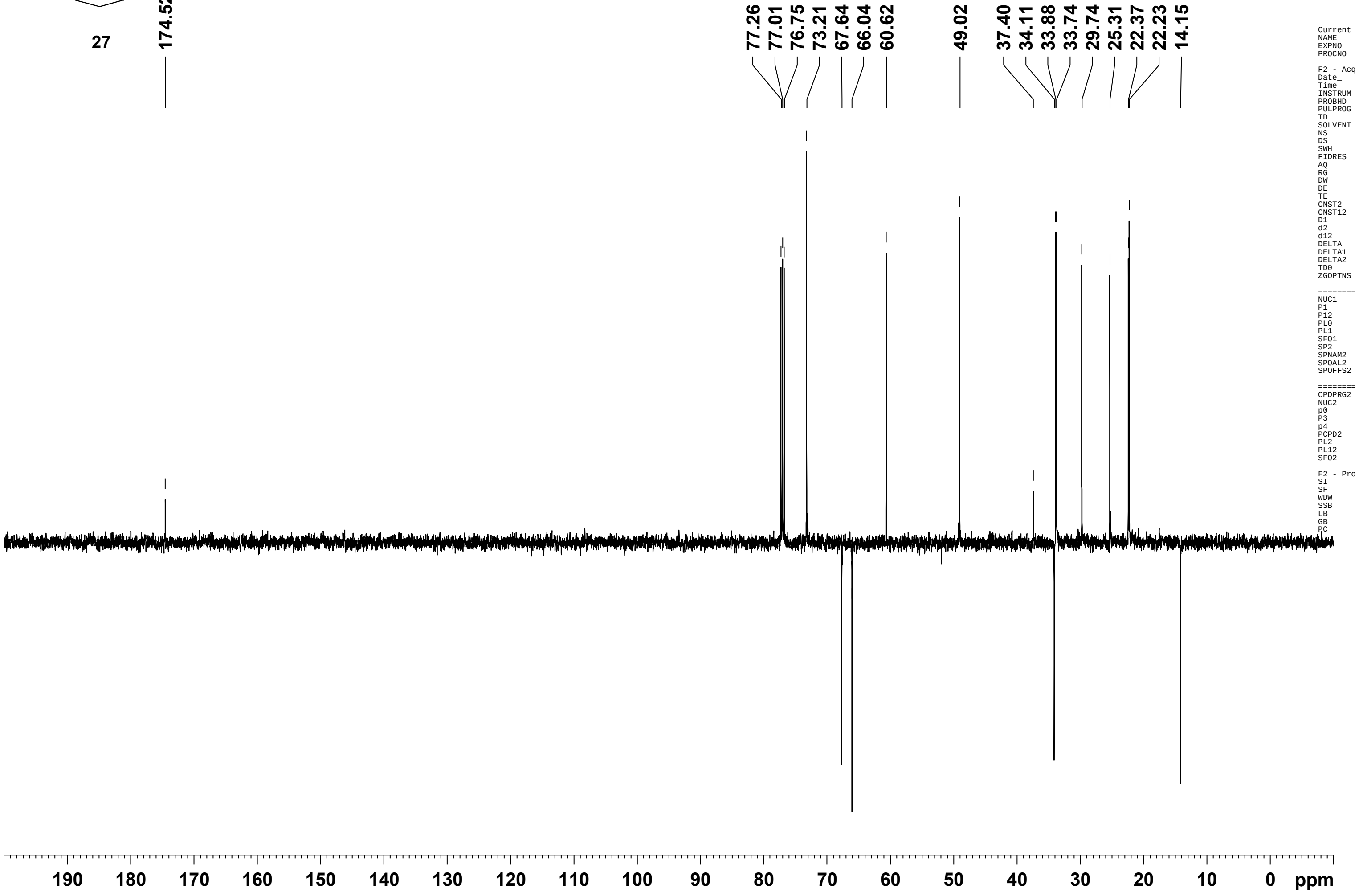


```
Current Data Parameters
NAME                 27
EXPNO                 2
PROCNO                1

F2 - Acquisition Parameters
Date_          20080513
Time              11.32
INSTRUM          drx500
PROBHD   5 mm BBI 1H-BB
PULPROG         deptqsp
TD                65536
SOLVENT           CDCl3
NS                  464
DS                    8
SWH           31446.541 Hz
FIDRES         0.479836 Hz
AQ            1.0420724 sec
RG                16384
DW               15.900 usec
DE                 6.00 usec
TE                298.0 K
CNST2       145.0000000
CNST12        1.5000000
D1           2.00000000 sec
d2           0.00344828 sec
d12          0.00002000 sec
DELTA        0.00001878 sec
DELTA1       0.00348003 sec
DELTA2       0.00346303 sec
TD0                   1
ZGOPTNS

======== CHANNEL f1 ========
NUC1                13C
P1                14.75 usec
P12             2000.00 usec
PL0              120.00 dB
PL1               -3.00 dB
SFO1        125.7722511 MHz
SP2                1.78 dB
SPNAM2      Crp60comp.4
SPOAL2            0.500
SPOFFS2            0.00 Hz

======== CHANNEL f2 ========
CPDPRG2        waltz16
NUC2                 1H
p0                12.75 usec
P3                 8.50 usec
p4                17.00 usec
PCPD2             80.00 usec
PL2               -3.00 dB
PL12              16.47 dB
SFO2        500.1325007 MHz

F2 - Processing parameters
SI                32768
SF          125.7577927 MHz
WDW                  EM
SSB                   0
LB                 2.00 Hz
GB                    0
PC                 1.40
```

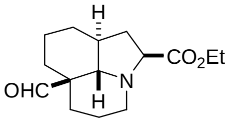
OHC
CO2Et
N
H
H

28

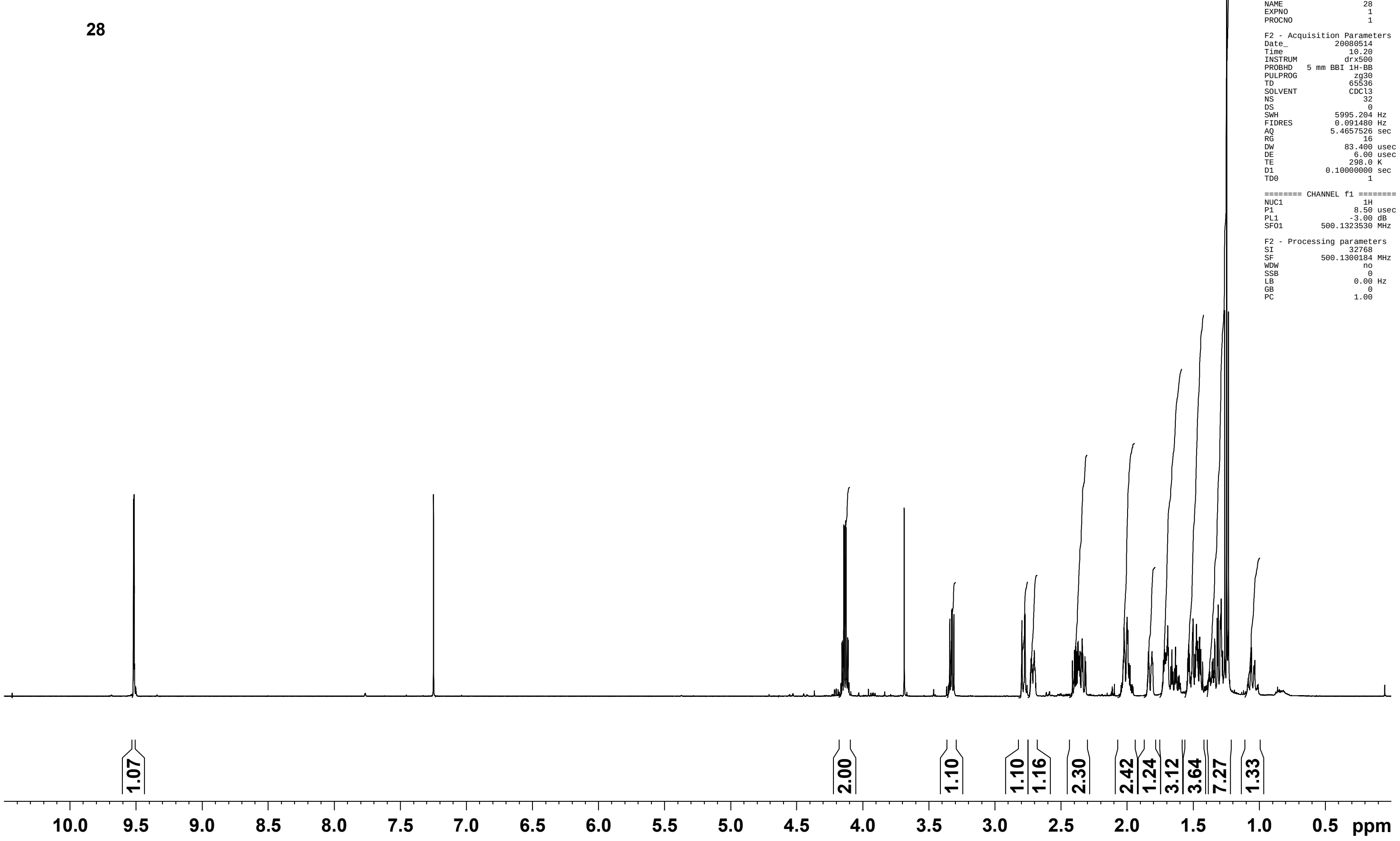
Current Data Parameters
NAME 28
EXPNO 1
PROCNO 1
F2 - Acquisition Parameters
Date_ 20080514
Time 10.20
INSTRUM drx500
PROBHD 5 mm BBI 1H-BB
PULPROG zg30
TD 65536
SOLVENT CDCl3
NS 32
DS 0
SWH 5995.204 Hz
FIDRES 0.091480 Hz
AQ 5.4657526 sec
RG 16
DW 83.400 usec
DE 6.00 usec
TE 298.0 K
D1 0.10000000 sec
TD0 1
======== CHANNEL f1 ========
NUC1 1H
P1 8.50 usec
PL1 -3.00 dB
SFO1 500.1323530 MHz
F2 - Processing parameters
SI 32768
SF 500.1300184 MHz
WDW no
SSB 0
LB 0.00 Hz
GB 0
PC 1.00
1.07
2.00
1.10
1.10
1.16
2.30
2.42
1.24
3.12
3.64
7.27
1.33
10.0 9.5 9.0 8.5 8.0 7.5 7.0 6.5 6.0 5.5 5.0 4.5 4.0 3.5 3.0 2.5 2.0 1.5 1.0 0.5 ppm

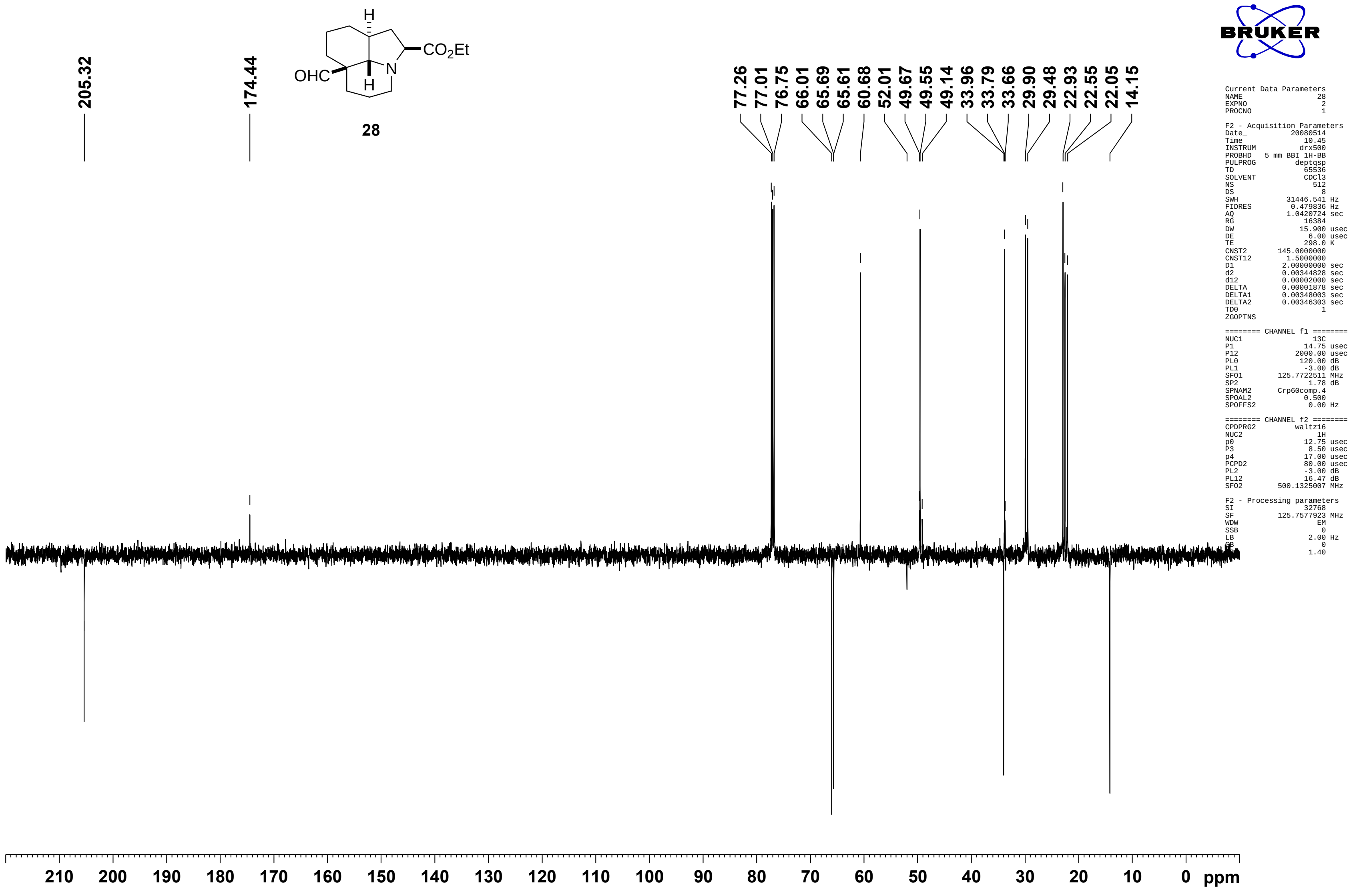
OHC
CO_2Et
H
N
28
205.32
174.44
77.26
77.01
76.75
66.01
65.69
65.61
60.68
52.01
49.67
49.55
49.14
33.96
33.79
33.66
29.90
29.48
22.93
22.55
22.05
14.15
210 200 190 180 170 160 150 140 130 120 110 100 90 80 70 60 50 40 30 20 10 0 ppm
Current Data Parameters
NAME 28
EXPNO 2
PROCNO 1
F2 - Acquisition Parameters
Date_ 20080514
Time 10.45
INSTRUM drx500
PROBHD 5 mm BBI 1H-BB
PULPROG deptqsp
TD 65536
SOLVENT CDCl3
NS 512
DS 8
SWH 31446.541 Hz
FIDRES 0.479836 Hz
AQ 1.0420724 sec
RG 16384
DW 15.900 usec
DE 6.00 usec
TE 298.0 K
CNST2 145.0000000
CNST12 1.5000000
D1 2.00000000 sec
d2 0.00344828 sec
d12 0.00002000 sec
DELTA 0.00001878 sec
DELTA1 0.00348003 sec
DELTA2 0.00346303 sec
TD0 1
ZGOPTNS
======== CHANNEL f1 ========
NUC1 13C
P1 14.75 usec
P12 2000.00 usec
PL0 120.00 dB
PL1 -3.00 dB
SFO1 125.7722511 MHz
SP2 1.78 dB
SPNAM2 Crp60comp.4
SPOAL2 0.500
SPOFFS2 0.00 Hz
======== CHANNEL f2 ========
CPDPRG2 waltz16
NUC2 1H
p0 12.75 usec
P3 8.50 usec
p4 17.00 usec
PCPD2 80.00 usec
PL2 -3.00 dB
PL12 16.47 dB
SFO2 500.1325007 MHz
F2 - Processing parameters
SI 32768
SF 125.7577923 MHz
WDW EM
SSB 0
LB 2.00 Hz
GB 0
1.40

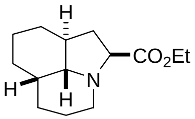
H
H
H
N
CO2Et

29

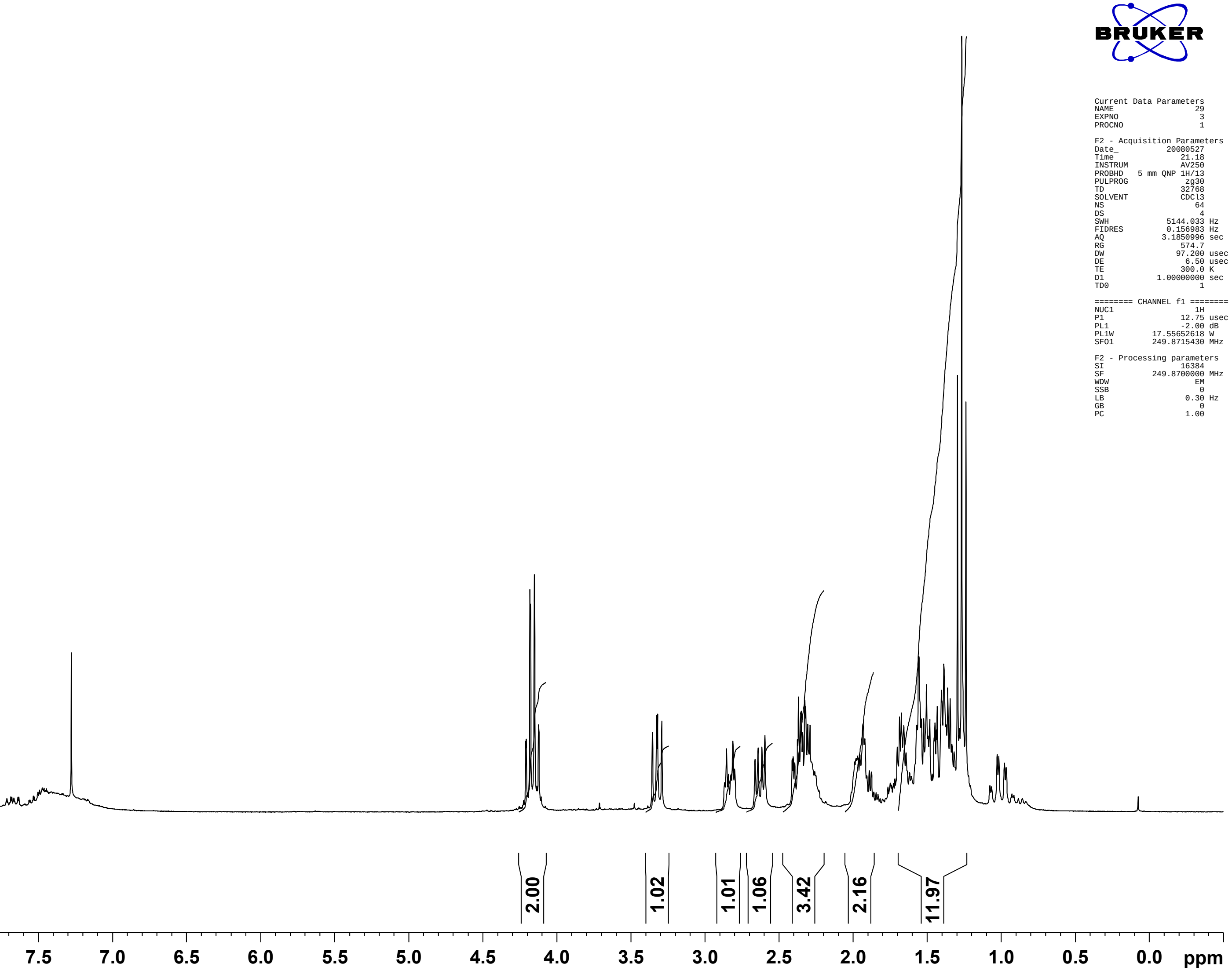
Current Data Parameters
NAME 29
EXPNO 3
PROCNO 1
F2 - Acquisition Parameters
Date_ 20080527
Time 21.18
INSTRUM AV250
PROBHD 5 mm QNP 1H/13
PULPROG zg30
TD 32768
SOLVENT CDCl3
NS 64
DS 4
SWH 5144.033 Hz
FIDRES 0.156983 Hz
AQ 3.1850996 sec
RG 574.7
DW 97.200 usec
DE 6.50 usec
TE 300.0 K
D1 1.00000000 sec
TD0 1
======== CHANNEL f1 ========
NUC1 1H
P1 12.75 usec
PL1 -2.00 dB
PL1W 17.55652618 W
SFO1 249.8715430 MHz
F2 - Processing parameters
SI 16384
SF 249.8700000 MHz
WDW EM
SSB 0
LB 0.30 Hz
GB 0
PC 1.00
2.00
1.02
1.01
1.06
3.42
2.16
11.97
8.5
8.0
7.5
7.0
6.5
6.0
5.5
5.0
4.5
4.0
3.5
3.0
2.5
2.0
1.5
1.0
0.5
0.0
ppm

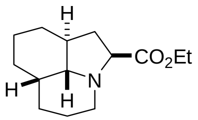

29

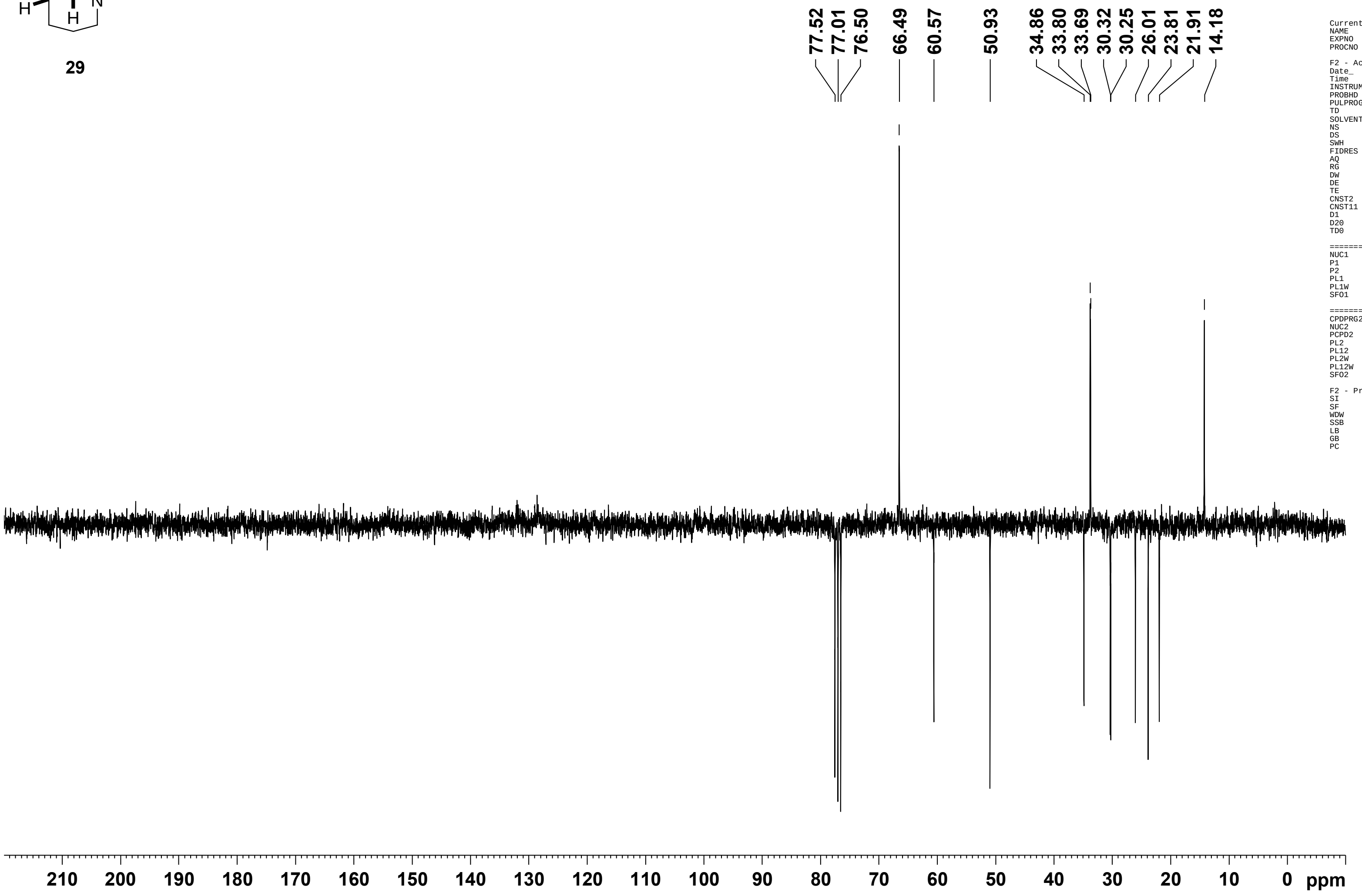


```
Current Data Parameters
NAME                29
EXPNO                4
PROCNO               1

F2 - Acquisition Parameters
Date_         20080527
Time             22.03
INSTRUM          AV250
PROBHD   5 mm QNP 1H/13
PULPROG           jmod
TD               32768
SOLVENT          CDCl3
NS                 512
DS                   4
SWH          15723.271 Hz
FIDRES        0.479836 Hz
AQ           1.0420724 sec
RG              2298.8
DW              31.800 usec
DE                6.50 usec
TE               300.0 K
CNST2      145.0000000
CNST11       1.0000000
D1          4.00000000 sec
D20         0.00689655 sec
TD0                  1

======== CHANNEL f1 ========
NUC1               13C
P1                9.00 usec
P2               18.00 usec
PL1               0.00 dB
PL1W       37.17591858 W
SFO1        62.8370864 MHz

======== CHANNEL f2 ========
CPDPRG2        waltz16
NUC2                1H
PCPD2            80.00 usec
PL2              -2.00 dB
PL12             13.95 dB
PL2W       17.55652618 W
PL12W       0.44610655 W
SFO2       249.8709995 MHz

F2 - Processing parameters
SI               16384
SF          62.8298610 MHz
WDW                 EM
SSB                  0
LB                1.00 Hz
GB                   0
PC                1.40
```
